# Analysis of global trends in acute lymphoblastic leukemia in children aged 0–5 years from 1990 to 2021

**DOI:** 10.3389/fped.2025.1542649

**Published:** 2025-03-13

**Authors:** Fang Ding, Lili Deng, Jingxuan Xiong, Zugen Cheng, Jiaoli Xu

**Affiliations:** ^1^ Department of Cardiovascular Medicine, Kunming Children's Hospital, Kunming City, Yunnan Province, China; ^2^ Department of Respiratory Medicine, Kunming Children's Hospital, Kunming City, Yunnan Province, China

**Keywords:** GBD (global burden of disease), children acute lymphoblastic leukemia, tumor, prevalence, mortality, DALYs—disability-adjusted life years

## Abstract

**Background:**

Acute lymphoblastic leukemia (ALL) is the most common pediatric cancer, with a significant global burden. This study evaluates global, regional, and national trends in the prevalence, mortality, and disability-adjusted life years (DALYs) of childhood ALL from 1990 to 2021, providing insights into disparities and progress across different socio-demographic and geographic contexts.

**Methods:**

Data were sourced from the Global Burden of Disease study. Trends in prevalence, mortality, and DALYs were analyzed by socio-demographic index (SDI) regions, geographic areas, and countries. Estimated annual percentage changes (EAPCs) were used to quantify temporal trends.

**Findings:**

Between 1990 and 2021, the global number of childhood ALL cases increased by 59.06%, reaching 168,879 cases in 2021, while ALL-related deaths and DALYs decreased by 66.71% and 66.13%, respectively. High- and high-middle SDI regions demonstrated significant improvements, driven by advances in healthcare and early diagnosis. In contrast, low-SDI regions faced persistent challenges, with a slight increase in DALYs observed in 2021. Geographic disparities were pronounced, with East Asia achieving the largest reductions in mortality and DALYs, whereas Sub-Saharan Africa and the Caribbean remained heavily burdened.

**Interpretation:**

Despite progress in reducing mortality and DALYs globally, the rising prevalence of ALL and persistent disparities in low-SDI regions highlight the urgent need for equitable access to healthcare, early diagnosis, and effective treatment strategies. Strengthening healthcare infrastructure and fostering global collaboration are critical to further mitigating the burden of childhood ALL and ensuring equitable outcomes worldwide.

## Introduction

Leukemia is a cancer of white blood cells originating in the bone marrow. When leukemia cells develop, they rapidly proliferate in the bone marrow, hindering the formation of healthy blood cells (1, 2). From 2000 to 2016, the incidence of acute leukemia (AL) in children, adolescents, and adults has been continuously increasing (3).

Acute lymphoblastic leukemia (ALL) is one of the most common types of leukemia in children (4), including B-type and T-type acute lymphoblastic leukemia (5). ALL predominantly occurs in children aged 2–5 years, comprising 75%–80% of pediatric leukemia cases (6). Due to the genetic heterogeneity of ALL, newly discovered genetic subtypes have refined risk stratification, increasing the complexity of diagnosis and treatment (7). The diagnosis of ALL requires precise procedures, including morphological, immunophenotypic, and cytogenetic analyses (8).

Although the survival rate of children and adolescents with ALL has significantly improved in high-income countries, approximately 80% of children with ALL in low-income countries still have poor prognoses, mainly due to a lack of diagnostic procedures, chemotherapy drugs, supportive care, and financial assistance (9). These challenges place a heavy burden on patients and their families.

Studies have shown that the peak incidence of ALL in children is mostly under five years old (10). Therefore, understanding the latest epidemiological data of ALL in this age group and analyzing its changing trends are crucial for addressing medical disparities, evaluating prevention and treatment effectiveness, and reducing the disease burden. However, there is currently a lack of research on the long-term global trends of ALL in children under five years old.

To fill this gap, we utilized data from the Global Burden of Disease (GBD) study from 1990 to 2021 to analyze the prevalence, mortality, and disability-adjusted life years (DALYs) of ALL in children under five at global, regional, and national levels, categorized by region, and time. Our study aims to provide a basis for clinicians and epidemiologists to formulate new prevention and treatment strategies, improve the prognosis of children with ALL, and optimize patient care, resource allocation, and public health measures.

## Methods

### Overview and data collection

This study is based purely on data analysis and does not involve identifiable personal information; therefore, informed consent is not required. We collected data for children under five years old with ALL from the GBD database. The data include the number of cases, related deaths, and DALYs at global, regional, and national levels, covering changes in case numbers from 1990 to 2021. While the incidence of ALL peaks around the age of three, this disease can occur at any age in children, and outcomes are often worse in older populations. In this study, we focus on children under five years old because WHO and other global health organizations use under-five mortality rates (U5MR) as a key health indicator (11). This age group is highly affected by infectious diseases and maternal and neonatal health-related issues, which are leading causes of childhood mortality.

All data are publicly available through the Global Health Data Exchange (GHDx) website (https://vizhub.healthdata.org/gbd-results/). We used linear regression to calculate the estimated annual percentage change (EAPC) to evaluate the influence of socioeconomic determinants on the burden of ALL (12), summarizing the burden distribution among children under five years old with ALL.

### Sociodemographic Index (SDI)

The SDI is a critical measure of a country's or region's development level, based on three key factors: fertility rate, education level, and per capita income (13). The SDI ranges from 0 to 1, with higher values indicating a higher degree of socio-economic development (14). SDI is strongly associated with disease mortality rates, serving as a proxy for broader health outcomes (15).

In this study, countries and regions are classified into five levels based on their SDI: high, high-middle, middle, low-middle, and low. This classification allows for an in-depth analysis of the relationship between the burden of ALL in children under 5 years old and socio-economic development. Countries with a higher SDI, which generally reflects better socio-economic conditions and more abundant healthcare resources, tend to have lower rates of ALL mortality. Conversely, countries with lower SDI face greater disease burdens due to limited healthcare infrastructure, public health services, and lower education levels.

By comparing countries across different SDI levels, this study aims to identify significant disparities in disease burden linked to socio-economic factors. Such insights are crucial for understanding the global distribution of ALL in young children and can inform targeted public health interventions.

### Statistical analysis

Previous studies have extensively outlined the methodology and framework of the GBD study (16). This research utilizes data from the GBD database to examine the burden of ALL in children under 5 years of age, covering global, regional, and national levels from 1990 to 2021. The data includes prevalence, mortality, DALYs, and their respective rates, all reported per 100,000 population, with 95% uncertainty intervals estimated using the GBD algorithms. To assess temporal trends in ALL disease burden, we calculated the EAPC and evaluated the time trend through its 95% confidence interval (CI). If the upper limit of the EAPC and its 95% CI are both negative, the corresponding rate shows a declining trend; conversely, if the lower limit and its 95% CI are both positive, the rate exhibits an increasing trend (17). All statistical analyses were performed using RStudio version 3.5.2, with two-sided *P*-values, and a significance threshold set at *P* < 0.05.

## Result

### Acute lymphoblastic leukemia in children: global trends

#### Prevalence

**Figure 1:**
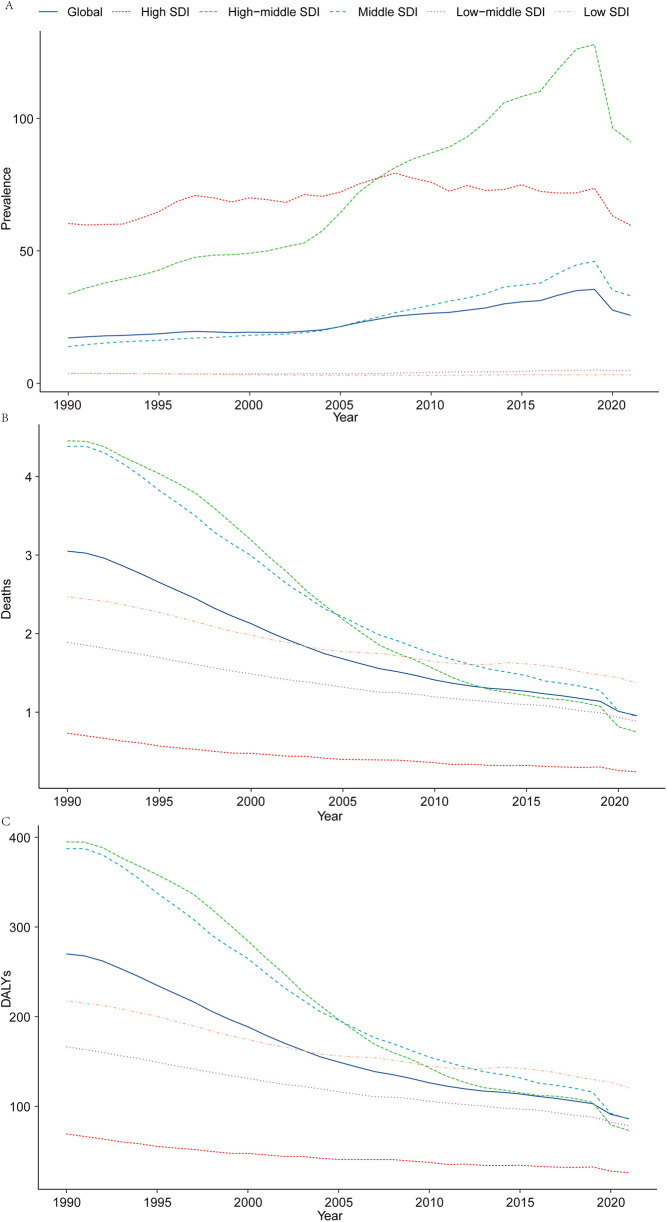
Epidemiologic trends of prevalence, death, and DALYs rates in 5 sociodemographic Index regions of acute lymphoblastic leukemia from 1990 to 2021. **(A)** Prevalence; **(B)** mortality; **(C)** DALYs.

In 2021, the global prevalence of ALL in children was estimated at 168,879.41 cases [95% uncertainty interval (UI): 103,720.68–240,540.11], reflecting a 59.06% increase from 106,172.66 cases in 1990 (95% UI: 88,471.54–133,096.57). The corresponding prevalence rate rose from 17.13 per 100,000 population in 1990 (95% UI: 14.27–21.47) to 25.66 per 100,000 in 2021 (95% UI: 15.76–36.55). The EAPC was 2.26 [95% confidence interval (CI): 1.93–2.60], highlighting a steady upward trend over the three-decade period (Table 1 and Figure 1A).

**Table 1:** Global and regional prevalence of acute lymphoblastic leukemia in children aged 0–5 years from 1990 to 2021.

Location	1990		2021		1990–2021	
	Prevalence cases	Prevalence rate	Prevalence cases	Prevalence rate	Cases change	EAPC^a^
Global	106,172.66 (88,471.54, 133,096.57)	17.13 (14.27, 21.47)	168,879.41 (103,720.68, 240,540.11)	25.66 (15.76, 36.55)	59.06 (−6.12, 146.38)	2.26 (1.93, 2.60)
SDI						
High SDI	37,251.72 (34,051.28, 40,630.55)	60.36 (55.18, 65.84)	32,085.56 (27,412.69, 37,036.98)	59.59 (50.91, 68.78)	−13.87 (−28.56, 1.86)	0.44 (0.14, 0.73)
High-middle SDI	31,305.71 (24,675.70, 39,872.11)	33.70 (26.56, 42.92)	63,899.10 (32,981.32, 100,496.14)	91.23 (47.09, 143.47)	104.11 (2.60, 242.05)	4.37 (3.95, 4.79)
Middle SDI	27,718.95 (19,548.32, 39,426.72)	13.82 (9.75, 19.66)	58,371.70 (33,973.65, 86,137.49)	33.05 (19.24, 48.77)	110.58 (4.56, 274.65)	3.92 (3.53, 4.30)
Low-middle SDI	6,349.48 (3,704.85, 10,886.62)	3.66 (2.14, 6.28)	9,201.46 (5,969.48, 12,075.70)	4.80 (3.12, 6.30)	44.92 (−23.38, 179.26)	1.16 (0.92, 1.40)
Low SDI	3,484.92 (1,605.79, 6,471.03)	3.84 (1.77, 7.13)	5,256.34 (2,822.06, 7,285.09)	3.17 (1.70, 4.40)	50.83 (−22.26, 248.93)	−0.54 (−0.76, −0.32)
Regions						
Andean Latin America	445.43 (301.66, 696.50)	8.43 (5.71, 13.19)	1,614.42 (770.36, 2,707.81)	26.23 (12.51, 43.99)	262.44 (35.65, 658.78)	4.78 (4.37, 5.18)
Australasia	616.37 (466.89, 828.43)	39.96 (30.27, 53.71)	913.15 (655.02, 1,201.32)	50.28 (36.07, 66.15)	48.15 (−3.72, 120.69)	1.69 (1.10, 2.28)
Caribbean	499.60 (363.32, 696.34)	12.09 (8.79, 16.85)	390.67 (280.20, 516.12)	10.10 (7.24, 13.34)	−21.80 (−46.00, 17.07)	0.03 (−0.25, 0.31)
Central Asia	788.42 (606.41, 1,016.13)	8.28 (6.37, 10.67)	954.33 (693.90, 1,360.48)	9.55 (6.94, 13.61)	21.04 (−17.11, 74.75)	1.15 (0.70, 1.59)
Central Europe	1,113.39 (924.56, 1,344.28)	12.19 (10.12, 14.72)	1,200.41 (896.60, 1,607.87)	21.49 (16.05, 28.79)	7.82 (−24.33, 57.78)	2.00 (1.56, 2.45)
Central Latin America	2,409.54 (2,144.00, 2,733.07)	10.47 (9.31, 11.87)	4,235.14 (3,086.43, 5,999.79)	21.08 (15.36, 29.86)	75.77 (25.87, 146.57)	3.03 (2.37, 3.69)
Central Sub-Saharan Africa	212.69 (56.01, 476.55)	2.05 (0.54, 4.59)	275.12 (132.80, 528.05)	1.31 (0.63, 2.51)	29.35 (−26.84, 299.33)	−1.09 (−1.26, −0.92)
East Asia	36,898.42 (24,575.00, 52,986.20)	31.88 (21.23, 45.78)	98,939.00 (48,299.18, 157,655.28)	123.56 (60.32, 196.89)	168.14 (17.71, 400.98)	5.88 (5.32, 6.44)
Eastern Europe	4,393.00 (3,727.96, 5,336.47)	25.48 (21.62, 30.95)	2,759.42 (2,320.39, 3,307.22)	27.27 (22.93, 32.68)	−37.19 (−50.30, −21.84)	0.39 (−0.36, 1.14)
Eastern Sub-Saharan Africa	1,923.40 (944.46, 3,418.75)	5.33 (2.62, 9.47)	2,653.90 (1,341.60, 4,620.51)	4.16 (2.10, 7.24)	37.98 (−37.74, 237.98)	−0.81 (−1.10, −0.50)
High-income Asia Pacific	5,853.87 (4,227.42, 8,140.71)	57.30 (41.38, 79.69)	3,986.94 (3,208.79, 4,860.59)	61.79 (49.73, 75.33)	−31.89 (−54.36, 2.63)	0.56 (0.11, 1.02)
High-income North America	16,645.70 (15,242.70, 18,113.97)	76.77 (70.30, 83.54)	11,292.96 (9,832.11, 12,859.84)	55.09 (47.96, 62.74)	−32.16 (−41.81, −20.00)	−0.54 (−0.84, −0.24)
North Africa and Middle East	4,343.87 (2,561.52, 7,096.63)	8.48 (5.00, 13.85)	9,096.48 (5,145.99, 12,967.42)	14.88 (8.42, 21.21)	109.41 (9.95, 336.46)	2.68 (2.31, 3.05)
Oceania	20.73 (10.06, 38.48)	2.06 (1.00, 3.83)	42.05 (21.91, 77.08)	2.17 (1.13, 3.98)	102.88 (31.47, 216.98)	0.11 (−0.20, 0.43)
South Asia	3,766.42 (1,860.69, 7,167.27)	2.40 (1.18, 4.56)	4,368.77 (2,688.26, 6,315.81)	2.75 (1.70, 3.98)	15.99 (−45.64, 174.76)	0.46 (0.06, 0.86)
Southeast Asia	3,638.20 (1,713.25, 6,779.32)	6.24 (2.94, 11.63)	5,940.13 (4,124.21, 8,025.01)	10.55 (7.33, 14.26)	63.27 (−12.66, 261.65)	1.93 (1.75, 2.12)
Southern Latin America	542.72 (435.33, 677.62)	10.54 (8.46, 13.17)	903.39 (633.65, 1,287.48)	21.11 (14.81, 30.09)	66.46 (8.98, 154.89)	2.66 (2.21, 3.10)
Southern Sub-Saharan Africa	149.72 (80.40, 260.83)	2.00 (1.08, 3.49)	206.82 (134.12, 285.69)	2.58 (1.67, 3.56)	38.14 (−16.90, 160.12)	1.94 (0.80, 3.09)
Tropical Latin America	1,040.50 (875.72, 1,233.38)	6.09 (5.13, 7.22)	1,773.31 (1,332.44, 2,349.97)	10.31 (7.74, 13.66)	70.43 (24.47, 136.14)	2.33 (1.98, 2.67)
Western Europe	19,932.33 (17,683.54, 22,452.47)	86.83 (77.03, 97.80)	15,138.00 (12,915.75, 17,746.86)	71.31 (60.84, 83.60)	−24.05 (−38.16, −9.00)	−0.59 (−0.91, −0.27)
Western Sub-Saharan Africa	938.35 (361.52, 1,618.64)	2.63 (1.01, 4.53)	2,194.99 (575.70, 3,621.95)	2.75 (0.72, 4.53)	133.92 (13.84, 366.53)	0.38 (0.20, 0.57)

EAPC, estimated annual percentage change; SDI, sociodemographic Index; UI, uncertainty interval. EAPC^a^ is expressed as 95% CIs.

#### Mortality

In 2021, ALL-related mortality in children was reported at 6,294.10 deaths (95% UI: 3,933.74–8,350.67), representing a significant 66.71% decline from 18,904.74 deaths in 1990 (95% UI: 12,759.66–28,582.64). The corresponding mortality rate decreased from 3.05 per 100,000 population in 1990 (95% UI: 2.06–4.61) to 0.96 per 100,000 in 2021 (95% UI: 0.60–1.27). The EAPC was −3.63 (95% CI: −3.78 to −3.48), underscoring a remarkable reduction in ALL-related mortality over the study period (Table 2 and Figure 1B).

**Table 2:** Global and regional deaths of acute lymphoblastic leukemia in children aged 0–5 years from 1990 to 2021.

Location	1990		2021		1990–2021	
	Deaths cases	Deaths rate	Deaths cases	Deaths rate	Cases change	EAPC^a^
Global	18,904.74 (12,759.66, 28,582.64)	3.05 (2.06, 4.61)	6,294.10 (3,933.74, 8,350.67)	0.96 (0.60, 1.27)	−66.71 (−80.26, −45.45)	−3.63 (−3.78, −3.48)
SDI						
High SDI	452.07 (413.06, 509.19)	0.73 (0.67, 0.83)	130.40 (110.38, 150.61)	0.24 (0.20, 0.28)	−71.16 (−76.52, −65.60)	−3.07 (−3.24, −2.90)
High-middle SDI	4,136.47 (2,884.53, 5,642.14)	4.45 (3.10, 6.07)	524.30 (319.64, 750.85)	0.75 (0.46, 1.07)	−87.32 (−92.75, −79.24)	−5.70 (−5.98, −5.42)
Middle SDI	8,787.20 (5,995.10, 12,955.47)	4.38 (2.99, 6.46)	1,668.48 (1,099.79, 2,240.00)	0.94 (0.62, 1.27)	−81.01 (−88.87, −69.21)	−4.73 (−4.90, −4.55)
Low-middle SDI	3,274.90 (1,745.20, 5,914.48)	1.89 (1.01, 3.41)	1,696.61 (1,100.55, 2,241.72)	0.89 (0.57, 1.17)	−48.19 (−71.33, 3.90)	−2.23 (−2.30, −2.16)
Low SDI	2,243.13 (1,007.79, 4,259.50)	2.47 (1.11, 4.69)	2,268.02 (1,182.67, 3,211.82)	1.37 (0.71, 1.94)	1.11 (−49.93, 145.07)	−1.77 (−1.88, −1.65)
Regions						
Andean Latin America	197.65 (132.34, 315.99)	3.74 (2.51, 5.98)	91.97 (51.47, 138.68)	1.49 (0.84, 2.25)	−53.47 (−79.48, −8.57)	−2.50 (−2.77, −2.23)
Australasia	9.22 (8.08, 10.50)	0.60 (0.52, 0.68)	3.25 (2.49, 4.04)	0.18 (0.14, 0.22)	−64.76 (−73.47, −54.55)	−3.09 (−3.30, −2.87)
Caribbean	133.91 (69.51, 240.28)	3.24 (1.68, 5.82)	79.97 (35.56, 155.72)	2.07 (0.92, 4.03)	−40.28 (−62.67, −10.71)	−1.02 (−1.25, −0.80)
Central Asia	208.90 (164.34, 260.14)	2.19 (1.73, 2.73)	94.39 (68.97, 136.82)	0.94 (0.69, 1.37)	−54.82 (−69.65, −33.94)	−2.55 (−2.72, −2.37)
Central Europe	119.64 (103.16, 139.06)	1.31 (1.13, 1.52)	16.84 (13.63, 20.96)	0.30 (0.24, 0.38)	−85.92 (−89.58, −81.00)	−4.67 (−4.82, −4.52)
Central Latin America	730.37 (653.45, 824.37)	3.17 (2.84, 3.58)	247.34 (179.94, 342.83)	1.23 (0.90, 1.71)	−66.14 (−75.44, −52.18)	−2.44 (−2.78, −2.11)
Central Sub-Saharan Africa	138.11 (35.68, 316.71)	1.33 (0.34, 3.05)	126.78 (62.29, 243.86)	0.60 (0.30, 1.16)	−8.20 (−47.80, 185.01)	−2.07 (−2.32, −1.81)
East Asia	9,480.52 (6,165.28, 13,943.90)	8.19 (5.33, 12.05)	961.20 (502.95, 1,484.42)	1.20 (0.63, 1.85)	−89.86 (−95.20, −81.73)	−6.04 (−6.38, −5.69)
Eastern Europe	339.32 (300.60, 378.86)	1.97 (1.74, 2.20)	48.61 (41.44, 57.58)	0.48 (0.41, 0.57)	−85.67 (−88.06, −82.97)	−4.94 (−5.42, −4.46)
Eastern Sub-Saharan Africa	1,247.44 (582.43, 2,235.67)	3.46 (1.61, 6.20)	1,127.91 (576.84, 1,897.60)	1.77 (0.90, 2.97)	−9.58 (−59.74, 133.36)	−2.04 (−2.20, −1.88)
High-income Asia Pacific	70.18 (59.01, 82.28)	0.69 (0.58, 0.81)	12.83 (10.38, 15.43)	0.20 (0.16, 0.24)	−81.73 (−85.53, −76.91)	−3.77 (−3.95, −3.60)
High-income North America	132.53 (127.87, 137.05)	0.61 (0.59, 0.63)	48.75 (43.19, 54.88)	0.24 (0.21, 0.27)	−63.21 (−67.66, −58.48)	−2.41 (−2.59, −2.22)
North Africa and Middle East	1,216.67 (666.92, 2,079.66)	2.37 (1.30, 4.06)	540.74 (285.99, 786.67)	0.88 (0.47, 1.29)	−55.56 (−74.75, −14.79)	−2.75 (−2.95, −2.55)
Oceania	9.61 (4.36, 18.09)	0.96 (0.43, 1.80)	16.39 (8.05, 30.59)	0.85 (0.42, 1.58)	70.49 (14.13, 170.47)	−0.30 (−0.64, 0.03)
South Asia	2,171.96 (1,035.64, 4,191.57)	1.38 (0.66, 2.67)	957.06 (643.86, 1,362.94)	0.60 (0.41, 0.86)	−55.94 (−76.45, −5.20)	−2.65 (−2.77, −2.54)
Southeast Asia	1,515.93 (633.97, 3,000.22)	2.60 (1.09, 5.15)	769.97 (524.86, 1,053.47)	1.37 (0.93, 1.87)	−49.21 (−71.10, 13.82)	−1.87 (−1.94, −1.79)
Southern Latin America	67.21 (59.31, 76.03)	1.31 (1.15, 1.48)	22.21 (16.81, 29.35)	0.52 (0.39, 0.69)	−66.95 (−75.29, −55.55)	−2.28 (−2.51, −2.06)
Southern Sub-Saharan Africa	52.50 (28.84, 90.55)	0.70 (0.39, 1.21)	47.73 (29.85, 70.13)	0.59 (0.37, 0.87)	−9.10 (−44.13, 69.49)	0.48 (−0.27, 1.23)
Tropical Latin America	322.10 (268.40, 379.99)	1.89 (1.57, 2.22)	121.30 (88.60, 156.04)	0.70 (0.51, 0.91)	−62.34 (−72.79, −48.58)	−2.42 (−2.78, −2.06)
Western Europe	165.71 (157.48, 173.76)	0.72 (0.69, 0.76)	47.06 (40.84, 54.53)	0.22 (0.19, 0.26)	−71.60 (−75.65, −66.89)	−3.53 (−3.74, −3.32)
Western Sub-Saharan Africa	575.28 (224.23, 1,006.84)	1.61 (0.63, 2.82)	911.79 (244.45, 1,385.28)	1.14 (0.31, 1.73)	58.50 (−18.85, 210.71)	−0.83 (−0.95, −0.71)

EAPC, estimated annual percentage change; SDI, sociodemographic index; UI, uncertainty interval. EAPC^a^ is expressed as 95% CIs.

#### Disability-adjusted life years (DALYs)

In 2021, the global burden of ALL in terms of DALYs for children was estimated at 566,892.36 years (95% UI: 354,405.97–752,818.07), reflecting a 66.13% reduction from 1,673,557.22 years in 1990 (95% UI: 1,134,551.64–2,526,405.44). The corresponding DALY rate decreased from 269.96 per 100,000 population in 1990 (95% UI: 183.01–407.53) to 86.13 per 100,000 in 2021 (95% UI: 53.85–114.38). The EAPC was −3.56 (95% CI: −3.72 to −3.40), reflecting consistent progress in alleviating the burden of ALL globally (Supplementary Table S1 and Figure 1C).

These findings underscore significant advancements in reducing ALL-related mortality and disability worldwide, even as prevalence continues to rise. The results emphasize the importance of sustained efforts in early diagnosis, equitable access to advanced treatment modalities, and the development of targeted public health interventions to mitigate the burden of ALL among children further globally.

### Acute lymphoblastic leukemia in children: regional trends by SDI

#### Prevalence

In 2021, the burden of childhood ALL was highest in High-middle SDI regions, with an estimated 63,899.1 cases (95% UI, 32,981.32–100,496.14). In contrast, Low SDI regions reported the lowest burden, with 5,256.34 cases (95% UI, 2,822.06–7,285.09). Notably, the number of prevalent cases in Low SDI regions increased substantially by 50.83% (95% UI, −22.26% to 248.93%) since 1990. High-middle SDI regions exhibited the highest EAPC in prevalence rates (4.37; 95% CI, 3.95–4.79), whereas Low SDI regions saw the greatest reduction (EAPC, −0.54; 95% CI, −0.76 to −0.32) (Table 1 and Figure 1A).

#### Mortality

Among the five SDI regions, only the Low SDI region demonstrated an increase in childhood ALL-related mortality, rising by 1.11% between 1990 and 2021. In 1990, Middle SDI regions recorded the highest number of deaths (8,787.20; 95% UI, 5,995.10–12,955.47), but by 2021, this shifted to Low SDI regions, with 2,268.02 deaths (95% UI, 1,182.67–3,211.82). Mortality rates in 1990 were highest in High-middle SDI regions (4.45 per 100,000; 95% UI, 3.10–6.07) and lowest in High SDI regions (0.73 per 100,000; 95% UI, 0.67–0.83). By 2021, the highest mortality rate was observed in Low SDI regions (1.37 per 100,000; 95% UI, 0.71–1.94), while High SDI regions maintained the lowest rate (0.24 per 100,000; 95% UI, 0.20–0.28). High-middle SDI regions showed the largest reduction in mortality rates (EAPC, −5.70; 95% CI, −5.98 to −5.42) (Table 2 and Figure 1B).

#### DALYs

In 2021, Low SDI regions bore the highest burden of childhood ALL-related DALYs, with 199,781.1 (95% UI, 104,217.91–282,873.20). This represented a marginal increase of 1.06% (95% UI, −49.89% to 144.49%) compared to 1990 (197,688.00; 95% UI, 89,015.38–375,199.42). High-middle SDI regions exhibited the largest decline in DALY rates (EAPC, −5.38; 95% CI, −5.65 to −5.11) (Supplementary Table S1 and Figure 1C).

These findings underscore substantial regional disparities in the burden of childhood ALL, highlighting an urgent need for targeted strategies to mitigate the rising burden in Low SDI regions and to sustain progress in High SDI regions.

### Acute lymphoblastic leukemia in children: geographic regional trends

#### Prevalence

**Figure 2:**
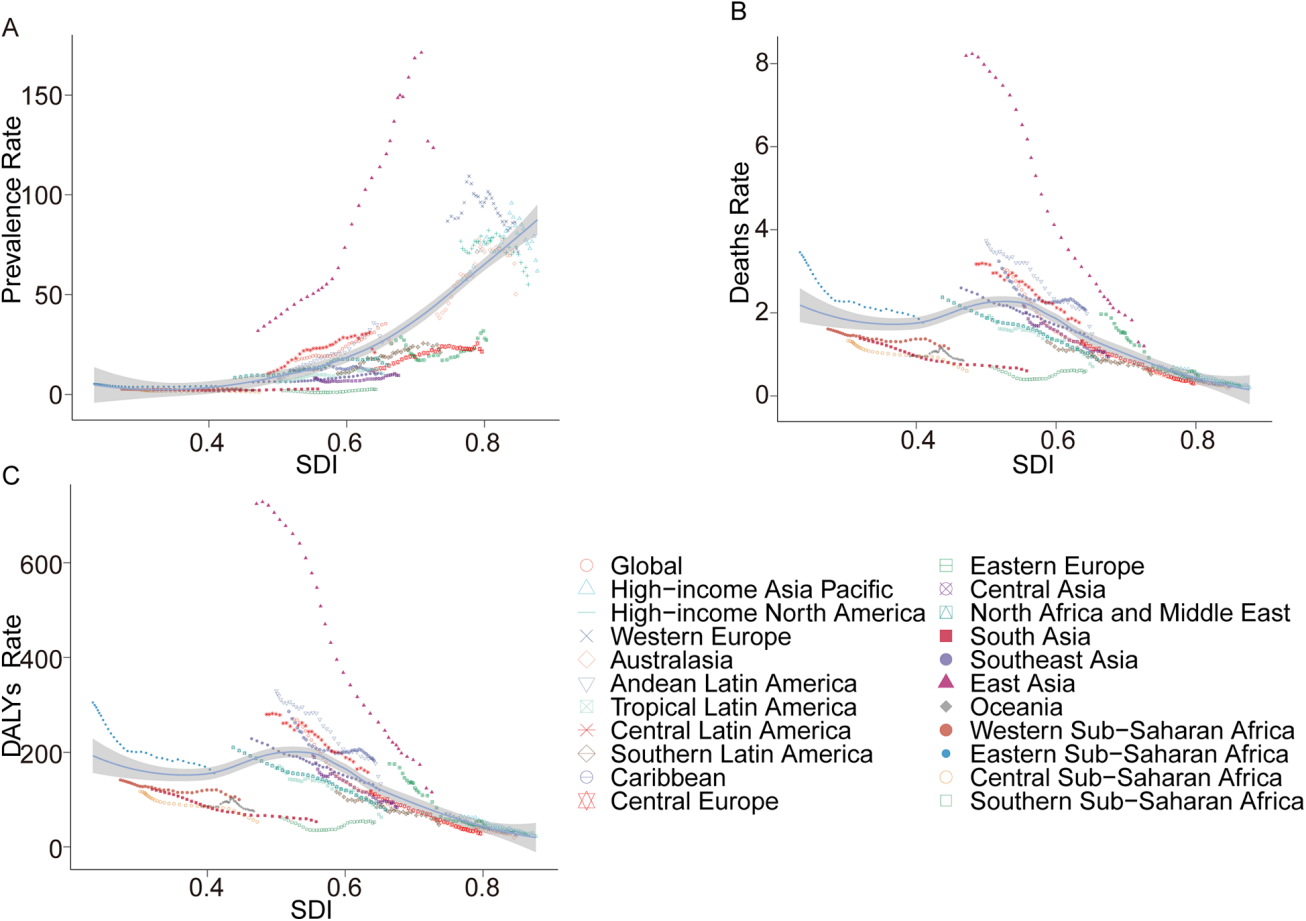
Prevalence, death, and DALYs rates for acute lymphoblastic leukemia from 1990 to 2021. **(A)** Prevalence rate; **(B)** mortality; **(C)** DALYs rate.

In 2021, the prevalence of ALL in children exhibited marked regional disparities across 21 geographical areas. Oceania reported the lowest number of cases, with 42.05 (95% UI 21.91–77.08), while East Asia recorded the highest, reaching 98,939 cases (95% UI 48,299.18–157,655.28). Central Sub-Saharan Africa had the lowest prevalence rate at 1.31 per 100,000 children (95% UI 0.63–2.51), whereas East Asia registered the highest rate at 123.56 per 100,000 children (95% UI 60.32–196.89). Notably, East Asia also experienced the largest annual percentage change in prevalence (EAPC 5.88, 95% CI 5.32–6.44) (Table 1). Globally, the SDI in 2021 was 0.67. Fourteen regions reported prevalence rates below the global average, while seven regions exceeded it (Figure 2A).

#### Mortality

In 2021, Australasia reported the fewest ALL-related deaths among children, with 3.25 deaths (95% UI 2.49–4.04), while Eastern Sub-Saharan Africa recorded the highest number, at 1,127.91 deaths (95% UI 576.84–1,897.60). Similarly, Australasia had the lowest mortality rate, at 0.18 per 100,000 children (95% UI 0.14–0.22), whereas the Caribbean exhibited the highest rate, at 2.07 per 100,000 children (95% UI 0.92–4.03). Between 1990 and 2021, East Asia achieved the most significant reduction in mortality rate, with an EAPC of −6.04 (95% CI −6.38 to −5.69). In contrast, Southern Sub-Saharan Africa demonstrated the largest increase, with an EAPC of 0.48 (95% CI −0.27–1.23) (Table 2). Globally, the SDI in 2021 was 0.67, with 14 regions reporting mortality rates below the global average and seven regions exceeding it (Figure 2B).

#### DALYs

In terms of DALYs, Australasia recorded the lowest burden, with 357.82 DALYs (95% UI 274.67–459.02), whereas Eastern Sub-Saharan Africa reported the highest, at 99,491.21 DALYs (95% UI 50,869.74–167,305.34). The lowest DALY rate was observed in Australasia, at 19.7 per 100,000 children (95% UI 15.12–25.28), while the Caribbean had the highest rate, at 182.58 per 100,000 children (95% UI 81.75–354.61). From 1990 to 2021, East Asia demonstrated the largest reduction in DALY rates, with an EAPC of −5.77 (95% CI −6.10 to −5.44). Conversely, Southern Sub-Saharan Africa exhibited the largest increase, with an EAPC of 0.47 (95% CI −0.26–1.22) (Supplementary Table S1). Globally, the SDI in 2021 was 0.67. Fourteen regions recorded DALY rates below the global average, while seven regions were above it (Figure 2C).

### Acute lymphoblastic leukemia in children: national trends

#### Prevalence

**Figure 3:**
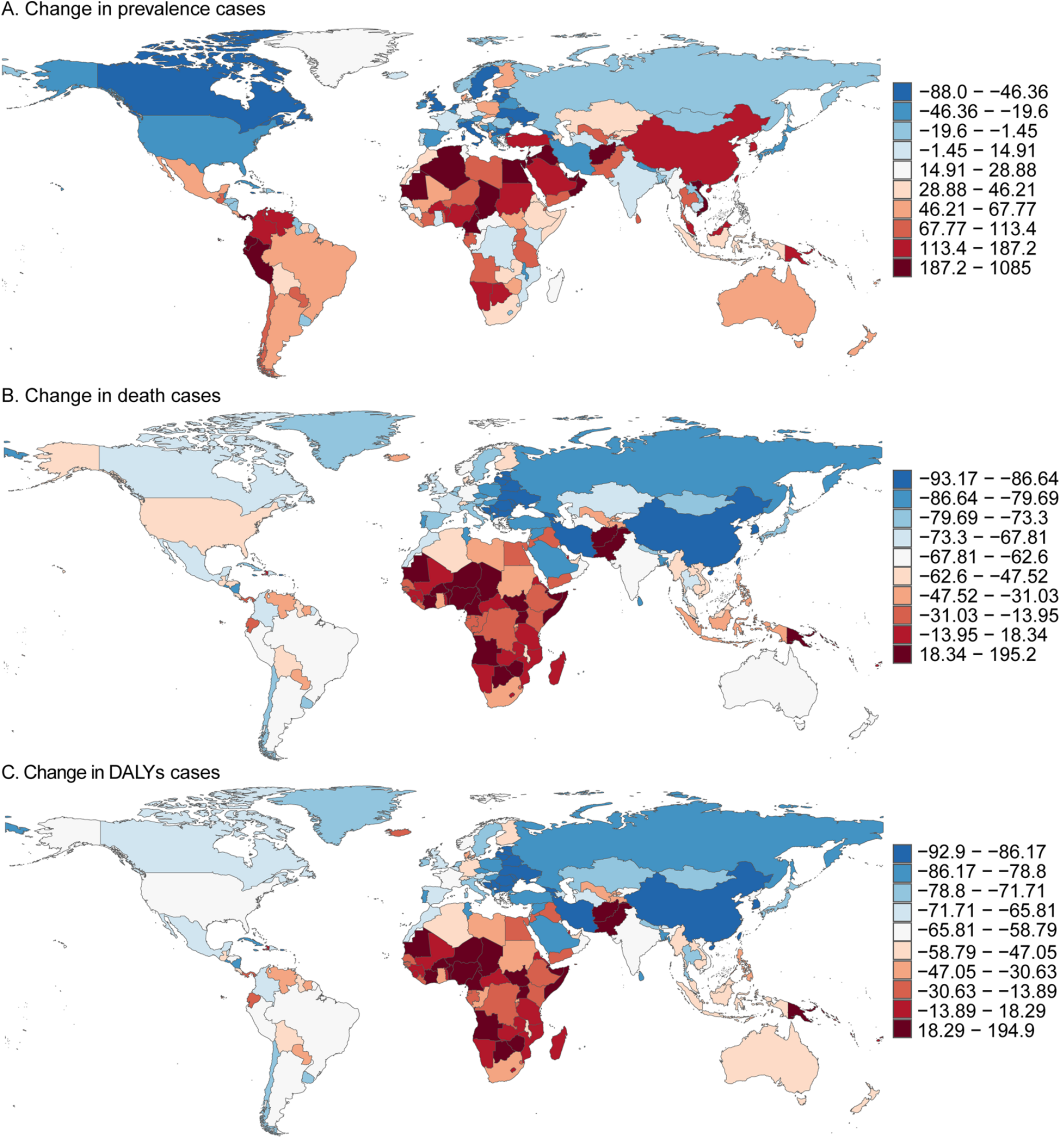
Prevalence, death, and DALYs cases of acute lymphoblastic leukemia in children in 204 countries and territories. **(A)** Change in prevalence cases. **(B)** Change in death cases. **(C)** Change in DALYs cases.

In 2021, among 204 countries, Palau reported the lowest number of childhood ALL cases, with 0.01 cases (95% UI, 0.00–0.05), while China had the highest, with 98,215.59 cases (95% UI, 47,784.03–156,575.12). The lowest prevalence of childhood ALL was observed in Vanuatu, at 0.72 per 100,000 (95% UI, 0.39–1.23), whereas Monaco reported the highest prevalence, at 399.44 per 100,000 (95% UI, 176.41–696.69). The largest decline in prevalence was recorded in Georgia, with an EAPC of −4.20% (95% CI, −4.96 to −3.44), while the Maldives saw the largest increase, with an EAPC of 6.63% (95% CI, 5.88–7.40). The prevalence rates were also closely correlated with the Socio-Demographic Index (SDI). Palau (SDI 0.75) had the lowest number of cases, whereas China (SDI 0.72) reported the highest. Vanuatu (SDI 0.47) exhibited the lowest prevalence rate, while Monaco (SDI 0.91) had the highest (Supplementary Table S3, Supplementary Figure S1A and Figure 3A).

#### Mortality

In 2021, several countries, including Cook Islands, Niue, Northern Mariana Islands, Palau, Tokelau, and Tuvalu, reported no childhood deaths due to ALL. Conversely, China recorded the highest number of childhood ALL-related deaths, with 930.64 (95% UI, 487.57–1,433.88). Oman had the lowest mortality rate, with 0.08 per 100,000 (95% UI, 0.03–0.14), while Haiti reported the highest mortality rate, at 3.87 per 100,000 (95% UI, 0.99–8.87). Serbia showed the largest reduction in mortality, with an EAPC of −6.94% (95% CI, −7.63 to −6.24), while Zimbabwe exhibited the most significant increase, with an EAPC of 2.53% (95% CI, 1.78–3.28). In line with prevalence, the mortality rates were strongly associated with SDI: China (SDI 0.72) reported the highest number of deaths, while Oman (SDI 0.77) had the lowest mortality rate, and Haiti (SDI 0.45) had the highest (Supplementary Table S4, Supplementary Figure S1B and Figure 3B).

#### DALYs

In 2021, Palau had the lowest number of childhood ALL-related DALYs, with 0.08 per 100,000 (95% UI, 0.01–0.40), while China had the highest, with 566,892.36 DALYs (95% UI, 354,405.97–752,818.07). Oman reported the lowest DALY rate, at 7.59 per 100,000 (95% UI, 3.14–13.21), while Haiti had the highest rate, at 340.33 per 100,000 (95% UI, 87.74–780.27). Serbia recorded the largest decrease in the DALY rate, with an EAPC of −6.76% (95% CI, −7.45 to −6.07), whereas Zimbabwe showed the largest increase, with an EAPC of 2.50% (95% UI, 1.77–3.24). Consistent with trends in prevalence and mortality, DALY rates were strongly influenced by SDI. Palau (SDI 0.75) had the lowest number of DALYs, while China (SDI 0.72) had the highest. Oman (SDI 0.77) had the lowest DALY rate, and Haiti (SDI 0.45) had the highest (Supplementary Table S2, Supplementary Figure S1C and Figure 3C).

## Discussion

Childhood ALL arises from the aberrant proliferation of precursor T and B lymphoid cells in hematopoietic tissues such as the bone marrow and thymus (18, 19). It is the most common pediatric malignancy worldwide, accounting for approximately 15% of new cancer cases among individuals under 15 years of age (20). In the United States, the annual incidence of ALL is approximately 40 cases per million population in children aged 0–14 years (21), with around 3,100 new diagnoses reported each year among individuals younger than 20 years (22). Since the 1970s, the incidence of ALL has shown a steady increase, peaking at approximately 81 cases per million in children aged 1–4 years—four times higher than the incidence observed in infants and children aged 10 years or older (21). Although advancements in therapy have markedly improved the overall cure rates of ALL, a substantial proportion of children and adolescents experience relapse after standard treatment, which is associated with significantly reduced survival rates (23). The rising medical and societal costs of ALL have further underscored its status as a pressing global public health challenge, prompting increasing attention to its burden and the development of effective strategies for prevention, treatment, and care.

This study provides the first comprehensive global assessment of temporal trends in the burden of childhood ALL under 5 years of age between 1990 and 2021, establishing a valuable epidemiological foundation for future research. Despite overall reductions in mortality and DALYs observed worldwide, certain regions and countries have experienced a rising disease burden, underscoring the heterogeneous progress and persisting inequities in ALL outcomes. These findings not only enhance our understanding of the evolving global epidemiology of ALL but also offer critical guidance for developing targeted prevention and control strategies. Improving healthcare infrastructure, optimizing resource allocation, and promoting early detection and intervention remain central priorities for clinicians and public health policymakers seeking to reduce the global burden of childhood ALL.

The observed increase in ALL prevalence may reflect a dynamic interplay with socioeconomic development in this study, potentially unfolding through four transitional stages. Underdiagnosis phase: Limited healthcare access and diagnostic capabilities in early development result in substantial undiagnosed and misdiagnosed cases. Healthcare engagement phase: Economic growth and national health policies improve healthcare accessibility, reducing non-attendance at medical facilities and increasing case detection. Diagnostic refinement phase: Advancements in cancer diagnostics (e.g., flow cytometry, molecular testing) decrease misdiagnosis rates, particularly in distinguishing ALL from other hematological malignancies. Outcome improvement phase: improvement in the outcome with advancements in cancer care and supportive care.

The etiology of ALL remains incompletely understood, with potential associations linked to viral infections, immune dysregulation, physical and chemical exposures, and genetic predisposition (24, 25). However, the precise contribution of each factor to the pathogenesis of pediatric ALL remains unclear and likely varies by environmental and geographical contexts. These disparities may underpin the regional differences in ALL incidence and associated mortality rates. The rapid increase in the global burden of malignancies has been attributed, in part, to changing modern environmental patterns, yet the environmental risk factors specifically associated with pediatric hematologic malignancies remain poorly characterized (26). Emerging evidence suggests a protective association between ultraviolet radiation (UVR) exposure and pediatric ALL risk (27), while rare genetic conditions, such as Down syndrome, are established risk factors for leukemia (28, 29). Among environmental exposures, moderate-to-high doses of ionizing radiation are recognized as leukemogenic (30). Conversely, the role of non-ionizing radiation, particularly extremely low-frequency magnetic fields from high-voltage power lines, remains controversial, with no definitive conclusions reached (31, 32). Additionally, benzene, a known carcinogen, continues to be a significant consideration in the context of ALL pathogenesis (22). In summary, effective intervention strategies targeting these risk factors are critical for reducing the burden of ALL and improving outcomes. A deeper understanding of these etiological pathways and their interplay with environmental and genetic factors is essential to inform evidence-based prevention and management strategies.

## Limitations

This study is subject to several limitations. First, the data analyzed were derived from multiple countries and regions, each with varying standards, methodologies, coverage, and quality of data collection. This variability is particularly pronounced in low- and middle-income countries, where data may lack detail or exhibit biases, potentially underrepresenting the health status of children under five years of age. Second, the GBD dataset provides aggregated data, precluding the exploration of individual-level health conditions, environmental factors, and specific risk exposures—a limitation likely magnified during the COVID-19 pandemic, when disparities in data collection and reporting may have been further accentuated.

## Conclusion

This study highlights significant global progress in reducing the burden of childhood ALL over the past three decades, with notable declines in mortality and DALYs despite an increase in prevalence. Advances in diagnostics and treatment have driven improvements, particularly in high- and high-middle-SDI regions, yet low-SDI regions continue to face disproportionately high mortality and DALYs due to inequities in healthcare access and resources. Geographically, regions like East Asia have remarkably reduced mortality and DALYs, while Sub-Saharan Africa and the Caribbean remain heavily burdened. These findings underscore the urgent need to address disparities by strengthening healthcare infrastructure, expanding access to early diagnosis and cost-effective treatments, and prioritizing capacity-building for pediatric oncology in low-resource settings. Global collaboration and policy action are critical to mitigating the growing prevalence of ALL and ensuring equitable outcomes for children worldwide.

## Data Availability

The original contributions presented in the study are included in the article/Supplementary Material, further inquiries can be directed to the corresponding authors.

## Supplementary material

Supplementary Figure S1 Prevalence, deaths, and DALYs rates of acute lymphoblastic leukemia in children in 204 countries by SDI in 2021. **(A)** Prevalence rate; **(B)** deaths rate; **(C)** DALYs rate. DALYs, disability-adjusted life-years; SDI, socio-demographic index.
