# Supplementary figures and images for: Analysis of global trends in acute lymphoblastic leukemia in children aged 0–5 years from 1990 to 2021
Source: Front Pediatr. 2025 Mar 13;13:1542649. doi: 10.3389/fped.2025.1542649 (PMC11966407; doi:10.3389/fped.2025.1542649)

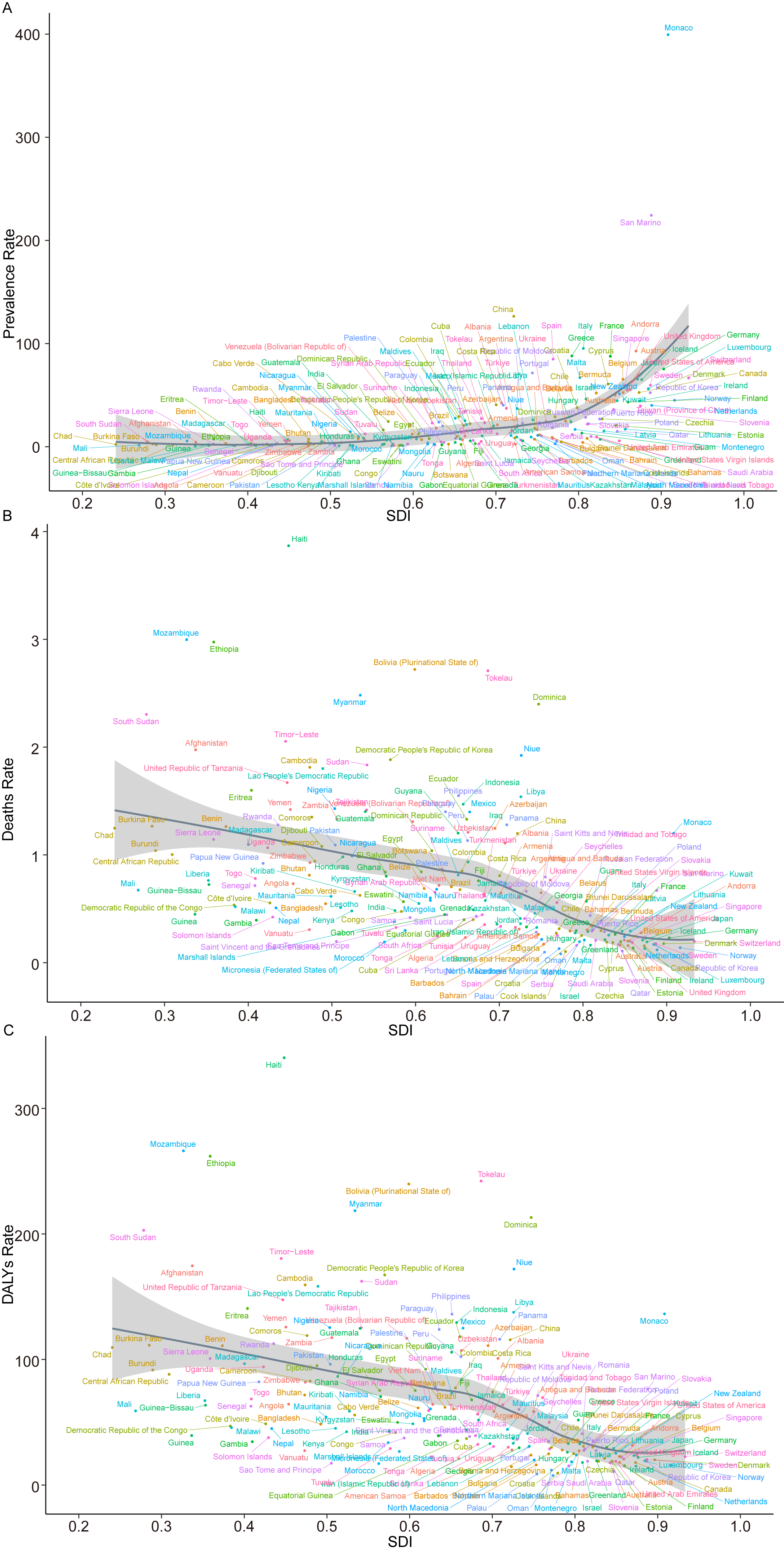

Supplement: Supplementary Figure S1 — Prevalence, deaths, and DALYs rates of acute lymphoblastic leukemia in children in 204 countries by SDI in 2021. (A) Prevalence rate; (B) deaths rate; (C) DALYs rate. DALYs, disability-adjusted life-years; SDI, socio-demographic index. [file Image1.tif]
