# Supplementary material for: Analysis of global trends in acute lymphoblastic leukemia in children aged 0–5 years from 1990 to 2021
Source: Front Pediatr. 2025 Mar 13;13:1542649. doi: 10.3389/fped.2025.1542649 (PMC11966407; doi:10.3389/fped.2025.1542649)
Supplement: Supplementary file 2 [file Table1.docx]

**Table S1.** Global and Regional DALYs of Acute Lymphoblastic Leukemia in Children Aged 0–5 Years from 1990 to 2021.

|  | 1990 |  |  | 2021 |  |  | 1990-2021 |  |
| --- | --- | --- | --- | --- | --- | --- | --- | --- |
| location | DALYs Cases | DALYs Rate |  | DALYs Cases | DALYs Rate |  | Cases change | EAPC^a^ |
| Global | 1673557.22(1134551.64,2526405.44) | 269.96(183.01,407.53) |  | 566892.36(354405.97,752818.07) | 86.13(53.85,114.38) |  | -66.13(-79.97,-44.24) | -3.56(-3.72,-3.40) |
| High SDI | 42764.14(39180.46,47899.49) | 69.30(63.49,77.62) |  | 14002.45(11793.58,16506.13) | 26.00(21.90,30.65) |  | -67.26(-73.28,-60.72) | -2.64(-2.80,-2.48) |
| High-middle SDI | 366899.21(256091.34,500334.32) | 394.93(275.66,538.56) |  | 51212.71(30875.48,74392.50) | 73.11(44.08,106.21) |  | -86.04(-92.20,-76.86) | -5.38(-5.65,-5.11) |
| Middle SDI | 776651.71(529721.89,1144089.69) | 387.29(264.15,570.52) |  | 151405.70(99487.51,204656.47) | 85.73(56.33,115.88) |  | -80.51(-88.63,-68.29) | -4.64(-4.82,-4.46) |
| Low-middle SDI | 288583.55(153977.94,521372.01) | 166.35(88.76,300.53) |  | 149930.58(97159.42,198052.38) | 78.26(50.72,103.38) |  | -48.05(-71.24,4.10) | -2.22(-2.29,-2.15) |
| Low SDI | 197688.00(89015.38,375199.42) | 217.73(98.04,413.24) |  | 199781.09(104217.91,282873.20) | 120.66(62.94,170.84) |  | 1.06(-49.89,144.49) | -1.76(-1.88,-1.65) |
| Regions |  |  |  |  |  |  |  |  |
| Andean Latin America | 17407.68(11662.91,27811.46) | 329.60(220.83,526.58) |  | 8196.91(4600.91,12361.69) | 133.16(74.74,200.81) |  | -52.91(-79.24,-7.38) | -2.46(-2.72,-2.19) |
| Australasia | 860.97(748.05,985.10) | 55.82(48.50,63.87) |  | 357.82(274.67,459.02) | 19.70(15.12,25.28) |  | -58.44(-68.65,-45.98) | -2.50(-2.72,-2.29) |
| Caribbean | 11816.35(6146.34,21171.61) | 286.01(148.77,512.45) |  | 7062.33(3162.28,13717.13) | 182.58(81.75,354.61) |  | -40.23(-62.62,-10.81) | -1.02(-1.24,-0.79) |
| Central Asia | 18477.42(14525.94,22997.85) | 194.00(152.51,241.46) |  | 8377.15(6123.98,12129.21) | 83.80(61.26,121.33) |  | -54.66(-69.54,-33.74) | -2.53(-2.70,-2.36) |
| Central Europe | 10629.37(9171.95,12357.31) | 116.40(100.44,135.33) |  | 1576.35(1270.29,1960.81) | 28.22(22.74,35.10) |  | -85.17(-89.00,-79.98) | -4.50(-4.65,-4.36) |
| Central Latin America | 64438.56(57707.78,72744.03) | 279.95(250.71,316.03) |  | 22140.77(16096.53,30700.31) | 110.21(80.12,152.81) |  | -65.64(-75.07,-51.33) | -2.40(-2.73,-2.06) |
| Central Sub-Saharan Africa | 12169.41(3169.75,27858.92) | 117.19(30.52,268.28) |  | 11147.27(5477.23,21380.32) | 52.91(26.00,101.49) |  | -8.40(-47.90,182.93) | -2.07(-2.32,-1.82) |
| East Asia | 838775.53(546262.31,1232471.60) | 724.65(471.94,1064.78) |  | 92597.73(48151.99,143967.35) | 115.64(60.13,179.79) |  | -88.96(-94.81,-80.01) | -5.77(-6.10,-5.44) |
| Eastern Europe | 30250.67(26812.04,33714.60) | 175.45(155.50,195.54) |  | 4507.12(3853.68,5326.16) | 44.54(38.08,52.64) |  | -85.10(-87.59,-82.30) | -4.82(-5.29,-4.34) |
| Eastern Sub-Saharan Africa | 110047.97(51476.31,197113.22) | 304.96(142.65,546.23) |  | 99491.21(50869.74,167305.34) | 155.95(79.74,262.25) |  | -9.59(-59.72,132.82) | -2.04(-2.20,-1.88) |
| High-income Asia Pacific | 6639.16(5565.67,7782.95) | 64.99(54.48,76.18) |  | 1441.90(1175.00,1776.62) | 22.35(18.21,27.54) |  | -78.28(-82.84,-72.35) | -3.17(-3.33,-3.00) |
| High-income North America | 12985.79(12429.74,13619.53) | 59.89(57.33,62.82) |  | 5181.30(4564.33,5954.37) | 25.28(22.27,29.05) |  | -60.10(-64.72,-54.88) | -2.15(-2.33,-1.97) |
| North Africa and Middle East | 107701.48(59122.64,183898.55) | 210.23(115.41,358.97) |  | 48338.84(25582.42,70182.38) | 79.07(41.84,114.80) |  | -55.12(-74.47,-13.80) | -2.71(-2.91,-2.51) |
| Oceania | 857.07(388.75,1610.23) | 85.35(38.71,160.35) |  | 1461.74(716.95,2720.07) | 75.56(37.06,140.61) |  | 70.55(14.07,170.68) | -0.30(-0.64,0.04) |
| South Asia | 191456.40(91413.53,369123.53) | 121.93(58.22,235.07) |  | 84655.65(57005.22,120495.38) | 53.38(35.94,75.98) |  | -55.78(-76.34,-4.79) | -2.64(-2.75,-2.52) |
| Southeast Asia | 133237.06(55761.36,263349.28) | 228.58(95.66,451.79) |  | 67923.73(46378.74,92743.80) | 120.68(82.40,164.78) |  | -49.02(-70.99,14.13) | -1.85(-1.93,-1.78) |
| Southern Latin America | 5978.80(5282.55,6764.56) | 116.16(102.63,131.43) |  | 2024.42(1535.68,2661.47) | 47.32(35.89,62.21) |  | -66.14(-74.74,-54.77) | -2.21(-2.43,-1.99) |
| Southern Sub-Saharan Africa | 4637.09(2554.85,7988.74) | 62.05(34.19,106.90) |  | 4216.12(2639.50,6183.71) | 52.51(32.87,77.02) |  | -9.08(-44.06,69.06) | 0.47(-0.26,1.22) |
| Tropical Latin America | 28469.14(23729.71,33550.87) | 166.69(138.94,196.44) |  | 10825.82(7905.39,13969.21) | 62.91(45.94,81.18) |  | -61.97(-72.48,-48.18) | -2.39(-2.75,-2.03) |
| Western Europe | 16163.21(15199.90,17176.84) | 70.41(66.21,74.82) |  | 5323.00(4582.95,6281.53) | 25.07(21.59,29.59) |  | -67.07(-71.54,-61.51) | -3.05(-3.22,-2.88) |
| Western Sub-Saharan Africa | 50558.09(19680.17,88497.67) | 141.44(55.06,247.58) |  | 80045.16(21448.77,121468.56) | 100.11(26.83,151.92) |  | 58.32(-18.90,209.98) | -0.83(-0.95,-0.71) |

Abbreviations: EAPC, estimated annual percentage change; SDI, Sociodemographic Index; UI, uncertainty interval. EAPC^a^ is expressed as 95% CIs.
