# Supplementary material for: Analysis of global trends in acute lymphoblastic leukemia in children aged 0–5 years from 1990 to 2021
Source: Front Pediatr. 2025 Mar 13;13:1542649. doi: 10.3389/fped.2025.1542649 (PMC11966407; doi:10.3389/fped.2025.1542649)
Supplement: Supplementary file 3 [file Table2.docx]

**Table S2.** National DALYs of Acute Lymphoblastic Leukemia in Children Aged 0–5 Years from 1990 to 2021.

| location | 1990 | |  | 2021 | |  | 1990-2021 | |
| --- | --- | --- | --- | --- | --- | --- | --- | --- |
|  | DALYs cases | DALYs rate |  | DALYs cases | DALYs rate |  | Cases change | EAPC |
| Afghanistan | 4811.87(688.85,11254.19) | 280.71(40.19,656.54) |  | 9570.97(4403.84,19595.20) | 174.61(80.34,357.48) |  | 98.90(5.98,866.06) | -1.21(-1.48,-0.95) |
| Albania | 690.58(357.43,1092.26) | 171.03(88.52,270.51) |  | 143.77(77.15,243.95) | 100.82(54.10,171.07) |  | -79.18(-89.08,-57.61) | -1.98(-2.44,-1.51) |
| Algeria | 2251.51(1139.87,3760.93) | 60.27(30.51,100.67) |  | 990.58(496.04,1671.00) | 21.06(10.54,35.52) |  | -56.00(-80.34,3.94) | -3.03(-3.15,-2.90) |
| American Samoa | 3.29(2.05,4.84) | 43.14(26.83,63.54) |  | 1.04(0.55,1.90) | 27.94(14.77,51.30) |  | -68.48(-84.51,-26.10) | -1.44(-1.66,-1.21) |
| Andorra | 3.38(1.34,6.76) | 124.82(49.62,249.61) |  | 0.80(0.42,1.26) | 31.59(16.68,50.03) |  | -76.40(-89.37,-26.17) | -3.61(-3.90,-3.32) |
| Angola | 2935.22(471.33,7422.00) | 150.36(24.14,380.19) |  | 3626.24(1643.48,7782.55) | 64.37(29.17,138.16) |  | 23.54(-40.49,450.92) | -2.49(-2.75,-2.22) |
| Antigua and Barbuda | 4.41(3.37,5.58) | 72.72(55.61,92.11) |  | 3.08(2.32,3.84) | 58.48(43.94,72.83) |  | -30.05(-48.34,-8.29) | -0.03(-0.47,0.42) |
| Argentina | 4319.26(3693.33,5154.27) | 125.68(107.46,149.97) |  | 1640.07(1207.09,2185.14) | 54.74(40.29,72.93) |  | -62.03(-73.14,-46.15) | -2.06(-2.29,-1.82) |
| Armenia | 1007.10(852.91,1203.39) | 263.00(222.73,314.26) |  | 109.13(78.07,149.72) | 58.61(41.93,80.40) |  | -89.16(-92.51,-84.41) | -4.45(-4.94,-3.96) |
| Australia | 674.01(569.82,793.11) | 53.38(45.13,62.81) |  | 278.80(206.57,373.41) | 18.54(13.74,24.84) |  | -58.64(-70.64,-42.83) | -2.51(-2.74,-2.28) |
| Austria | 253.07(211.75,301.11) | 56.61(47.37,67.36) |  | 77.85(58.59,105.46) | 18.02(13.56,24.41) |  | -69.24(-77.83,-55.90) | -3.42(-3.67,-3.17) |
| Azerbaijan | 2414.85(1486.89,3457.38) | 267.20(164.52,382.56) |  | 800.35(412.59,1434.78) | 111.00(57.22,198.99) |  | -66.86(-83.78,-37.26) | -2.56(-2.71,-2.41) |
| Bahamas | 17.37(13.59,21.47) | 68.09(53.30,84.21) |  | 5.73(3.63,8.80) | 27.24(17.23,41.78) |  | -66.98(-79.06,-46.92) | -2.32(-2.53,-2.12) |
| Bahrain | 32.36(18.00,54.68) | 52.69(29.30,89.03) |  | 15.03(8.23,27.40) | 16.04(8.78,29.24) |  | -53.55(-73.99,-16.09) | -3.22(-3.57,-2.87) |
| Bangladesh | 34667.75(9718.07,78426.40) | 183.13(51.33,414.28) |  | 6957.37(4097.79,11437.85) | 48.44(28.53,79.63) |  | -79.93(-92.98,-23.54) | -4.19(-4.31,-4.07) |
| Barbados | 11.60(8.81,14.50) | 59.22(44.97,74.02) |  | 3.24(2.14,4.83) | 23.81(15.68,35.48) |  | -72.05(-81.15,-58.96) | -2.97(-3.43,-2.50) |
| Belarus | 1213.71(961.42,1496.45) | 150.63(119.32,185.72) |  | 130.32(81.01,204.19) | 27.88(17.33,43.69) |  | -89.26(-93.41,-81.57) | -5.21(-5.85,-4.56) |
| Belgium | 461.52(386.12,560.42) | 77.37(64.73,93.95) |  | 134.62(97.13,185.13) | 22.74(16.41,31.28) |  | -70.83(-79.82,-56.96) | -3.72(-4.16,-3.28) |
| Belize | 72.49(61.16,84.61) | 245.39(207.04,286.40) |  | 23.42(17.94,30.17) | 61.43(47.05,79.11) |  | -67.69(-76.16,-56.53) | -3.89(-4.18,-3.61) |
| Benin | 1337.98(585.20,2343.01) | 135.60(59.31,237.46) |  | 2598.42(725.99,5138.91) | 110.93(30.99,219.39) |  | 94.20(-25.76,373.43) | -0.22(-0.42,-0.01) |
| Bermuda | 2.01(1.09,3.34) | 46.86(25.48,77.77) |  | 0.77(0.51,1.13) | 30.11(19.93,44.28) |  | -61.84(-78.25,-20.48) | -1.02(-1.45,-0.59) |
| Bhutan | 138.23(46.10,285.49) | 144.80(48.29,299.04) |  | 43.76(23.03,75.40) | 71.70(37.74,123.54) |  | -68.34(-88.86,11.91) | -2.75(-3.14,-2.35) |
| Bolivia (Plurinational State of) | 5633.49(2519.48,10465.72) | 557.80(249.47,1036.26) |  | 2862.93(1609.31,4570.26) | 239.74(134.76,382.71) |  | -49.18(-79.46,54.31) | -2.65(-2.75,-2.54) |
| Bosnia and Herzegovina | 172.67(104.70,255.74) | 49.11(29.78,72.74) |  | 22.02(12.11,35.06) | 14.58(8.02,23.21) |  | -87.25(-93.26,-75.90) | -3.47(-3.97,-2.98) |
| Botswana | 99.20(47.20,176.00) | 46.57(22.16,82.62) |  | 145.77(67.16,284.24) | 61.90(28.52,120.70) |  | 46.94(-22.28,208.01) | 2.08(1.46,2.71) |
| Brazil | 27169.77(22424.59,32154.03) | 165.04(136.22,195.32) |  | 10020.36(7420.23,12813.02) | 60.52(44.81,77.38) |  | -63.12(-72.96,-49.51) | -2.48(-2.84,-2.11) |
| Brunei Darussalam | 16.77(9.57,25.71) | 48.62(27.76,74.56) |  | 9.49(4.81,14.98) | 30.72(15.56,48.47) |  | -43.39(-70.08,11.36) | -1.16(-1.41,-0.92) |
| Bulgaria | 627.73(484.67,788.59) | 116.94(90.29,146.90) |  | 75.15(47.40,111.78) | 24.83(15.66,36.93) |  | -88.03(-92.43,-81.66) | -4.39(-4.71,-4.06) |
| Burkina Faso | 2444.35(1056.42,4612.19) | 130.26(56.30,245.78) |  | 4572.84(1272.26,8465.86) | 111.36(30.98,206.16) |  | 87.08(-20.09,308.62) | 0.06(-0.15,0.27) |
| Burundi | 2297.48(1235.62,3835.95) | 213.70(114.93,356.80) |  | 1978.25(516.94,4492.87) | 91.57(23.93,207.97) |  | -13.89(-75.32,154.22) | -1.70(-2.18,-1.21) |
| Cabo Verde | 85.80(44.87,143.03) | 144.18(75.41,240.37) |  | 24.56(8.34,81.21) | 55.73(18.94,184.32) |  | -71.38(-91.25,-9.92) | -3.05(-3.35,-2.76) |
| Cambodia | 6196.15(1696.03,14190.47) | 339.51(92.93,777.54) |  | 2786.53(1649.70,4368.33) | 159.36(94.35,249.83) |  | -55.03(-80.54,92.11) | -2.75(-2.89,-2.61) |
| Cameroon | 2096.05(962.81,3500.96) | 104.47(47.99,174.49) |  | 4023.17(1241.25,6932.19) | 82.72(25.52,142.53) |  | 91.94(-10.81,300.55) | -0.06(-0.32,0.20) |
| Canada | 1052.26(887.92,1226.66) | 54.66(46.12,63.72) |  | 339.79(259.36,447.47) | 17.88(13.65,23.55) |  | -67.71(-76.24,-56.17) | -2.50(-2.80,-2.21) |
| Central African Republic | 671.37(158.63,1666.22) | 133.29(31.49,330.80) |  | 739.65(236.10,1721.86) | 88.18(28.15,205.27) |  | 10.17(-32.30,147.95) | -1.04(-1.19,-0.89) |
| Chad | 1349.87(529.30,2671.35) | 108.60(42.59,214.93) |  | 3980.71(1294.32,7912.66) | 109.50(35.60,217.65) |  | 194.90(52.21,569.66) | 0.48(0.34,0.63) |
| Chile | 1427.25(1222.34,1667.98) | 99.32(85.06,116.07) |  | 325.84(249.73,434.56) | 29.95(22.95,39.94) |  | -77.17(-82.90,-68.88) | -3.06(-3.30,-2.81) |
| China | 831108.02(540019.45,1222977.70) | 743.35(483.00,1093.84) |  | 89855.83(46552.02,139487.33) | 115.69(59.94,179.59) |  | -89.19(-94.92,-80.29) | -5.87(-6.21,-5.53) |
| Colombia | 9051.11(7511.19,10782.00) | 215.15(178.54,256.29) |  | 2662.27(1778.93,3977.28) | 77.33(51.67,115.53) |  | -70.59(-81.27,-54.28) | -1.02(-1.79,-0.24) |
| Comoros | 149.92(86.05,230.38) | 181.90(104.41,279.53) |  | 96.76(37.43,198.74) | 119.01(46.03,244.43) |  | -35.46(-74.12,43.46) | -1.20(-1.47,-0.94) |
| Congo | 382.88(133.07,836.20) | 97.06(33.73,211.97) |  | 264.20(138.32,473.88) | 41.75(21.86,74.88) |  | -31.00(-68.36,107.58) | -2.63(-2.88,-2.39) |
| Cook Islands | 0.36(0.20,0.57) | 16.10(9.06,25.58) |  | 0.11(0.03,0.26) | 9.50(2.48,23.00) |  | -70.38(-93.71,-6.97) | -4.81(-5.88,-3.72) |
| Costa Rica | 531.19(447.10,624.03) | 130.84(110.13,153.71) |  | 228.67(172.42,301.98) | 74.17(55.93,97.95) |  | -56.95(-67.60,-40.59) | -1.40(-1.59,-1.20) |
| Croatia | 1631.64(582.47,2768.26) | 70.62(25.21,119.81) |  | 2122.02(638.70,4423.59) | 48.73(14.67,101.59) |  | -67.08(-83.62,-35.73) | -0.83(-1.15,-0.51) |
| Cuba | 138.48(90.58,200.96) | 45.96(30.06,66.70) |  | 45.58(25.50,77.78) | 25.01(13.99,42.68) |  | -78.80(-85.89,-69.98) | -2.07(-2.40,-1.74) |
| Cyprus | 949.61(795.90,1110.91) | 106.09(88.92,124.11) |  | 201.36(140.30,277.41) | 36.98(25.77,50.95) |  | -56.69(-84.01,8.60) | -2.32(-2.72,-1.92) |
| Czechia | 36.74(20.44,61.88) | 57.50(31.99,96.84) |  | 15.91(7.45,28.81) | 21.21(9.93,38.39) |  | -81.44(-88.81,-69.63) | -2.98(-3.17,-2.80) |
| C么te d'Ivoire | 571.61(431.92,737.14) | 88.10(66.57,113.61) |  | 106.11(66.15,160.88) | 18.88(11.77,28.62) |  | 30.05(-42.72,196.82) | -4.03(-4.39,-3.66) |
| Democratic People's Republic of Korea | 7182.13(3518.22,11432.54) | 307.41(150.59,489.34) |  | 2532.05(1060.07,5637.89) | 167.34(70.06,372.60) |  | -64.75(-82.73,-31.01) | -1.55(-1.90,-1.19) |
| Democratic Republic of the Congo | 7990.38(2156.35,19063.15) | 109.52(29.56,261.30) |  | 6367.37(2797.61,12710.39) | 46.95(20.63,93.73) |  | -20.31(-56.40,160.68) | -2.08(-2.37,-1.78) |
| Denmark | 109.94(88.95,134.24) | 37.96(30.71,46.35) |  | 62.26(43.03,85.95) | 20.04(13.85,27.66) |  | -43.37(-62.11,-17.48) | -2.10(-2.53,-1.65) |
| Djibouti | 115.70(51.32,210.54) | 179.02(79.41,325.77) |  | 138.54(46.34,330.52) | 95.09(31.81,226.85) |  | 19.74(-47.60,147.90) | -1.65(-2.08,-1.23) |
| Dominica | 11.74(8.05,16.33) | 135.58(92.90,188.54) |  | 7.41(4.49,12.01) | 213.09(129.10,345.23) |  | -36.88(-62.72,10.64) | 1.83(1.48,2.19) |
| Dominican Republic | 3214.80(1761.33,4655.47) | 322.82(176.87,467.49) |  | 952.79(389.97,2048.24) | 92.22(37.75,198.26) |  | -70.36(-87.88,-25.94) | -3.64(-3.91,-3.36) |
| Ecuador | 2416.44(1993.28,2925.01) | 179.59(148.14,217.38) |  | 1962.69(1276.89,2864.92) | 118.15(76.87,172.47) |  | -18.78(-48.96,20.94) | -0.98(-1.41,-0.55) |
| Egypt | 12769.41(7100.13,26775.57) | 149.65(83.21,313.79) |  | 9814.63(4461.89,15698.82) | 75.28(34.22,120.41) |  | -23.14(-76.73,63.70) | -1.09(-1.56,-0.62) |
| El Salvador | 2415.81(1676.69,3471.38) | 313.71(217.73,450.78) |  | 452.28(216.00,792.91) | 75.29(35.96,131.99) |  | -81.28(-92.10,-59.21) | -4.36(-4.45,-4.27) |
| Equatorial Guinea | 88.72(21.98,216.89) | 107.86(26.72,263.67) |  | 66.03(23.68,133.60) | 35.25(12.64,71.31) |  | -25.57(-72.76,240.07) | -4.29(-4.61,-3.97) |
| Eritrea | 1131.04(524.38,2092.89) | 181.40(84.10,335.67) |  | 1290.88(534.42,2600.71) | 140.61(58.21,283.29) |  | 14.13(-60.55,236.84) | -0.75(-0.98,-0.53) |
| Estonia | 151.65(123.74,178.21) | 125.74(102.60,147.76) |  | 13.02(9.28,17.60) | 18.82(13.42,25.45) |  | -91.41(-94.11,-87.64) | -5.72(-6.15,-5.29) |
| Eswatini | 89.55(34.70,187.90) | 61.58(23.86,129.22) |  | 70.07(41.04,116.26) | 49.91(29.23,82.80) |  | -21.75(-58.79,104.30) | -0.27(-0.79,0.25) |
| Ethiopia | 52181.50(8037.15,114152.85) | 541.34(83.38,1184.24) |  | 41811.63(20098.90,93109.61) | 261.89(125.89,583.21) |  | -19.87(-62.64,266.35) | -2.57(-2.87,-2.27) |
| Fiji | 57.71(15.95,115.79) | 61.24(16.92,122.87) |  | 61.72(16.48,125.67) | 67.79(18.10,138.03) |  | 6.94(-44.58,89.71) | 0.46(0.26,0.67) |
| Finland | 79.14(65.28,96.75) | 25.31(20.88,30.94) |  | 37.28(26.99,51.27) | 15.35(11.11,21.12) |  | -52.90(-67.59,-31.89) | -1.46(-1.79,-1.13) |
| France | 2758.38(2388.68,3176.18) | 70.92(61.42,81.66) |  | 1059.18(807.51,1346.96) | 29.97(22.85,38.12) |  | -61.60(-71.41,-49.12) | -2.19(-2.61,-1.77) |
| Gabon | 100.84(42.75,197.71) | 64.56(27.37,126.59) |  | 83.77(37.12,148.02) | 39.23(17.39,69.32) |  | -16.93(-61.97,118.18) | -0.83(-1.17,-0.48) |
| Gambia | 122.90(48.35,205.88) | 66.19(26.04,110.87) |  | 123.38(41.32,290.28) | 34.50(11.55,81.18) |  | 0.38(-64.06,133.56) | -2.27(-2.59,-1.95) |
| Georgia | 852.45(627.53,1093.90) | 181.93(133.93,233.46) |  | 72.15(49.18,101.70) | 29.65(20.21,41.80) |  | -91.54(-94.31,-87.28) | -5.81(-6.48,-5.14) |
| Germany | 2551.60(2178.95,2966.00) | 56.97(48.65,66.22) |  | 1056.63(813.20,1397.95) | 26.13(20.11,34.57) |  | -58.59(-69.11,-43.42) | -2.76(-3.29,-2.23) |
| Ghana | 5286.90(1556.14,9938.31) | 200.72(59.08,377.31) |  | 3268.06(1216.47,7261.83) | 70.43(26.22,156.50) |  | -38.19(-80.18,80.05) | -3.86(-4.48,-3.24) |
| Greece | 1673557.22(1134551.64,2526405.44) | 269.96(183.01,407.53) |  | 566892.36(354405.97,752818.07) | 86.13(53.85,114.38) |  | -66.13(-79.97,-44.24) | -3.56(-3.72,-3.40) |
| Greenland | 363.40(317.26,415.13) | 65.48(57.17,74.80) |  | 139.67(112.43,175.79) | 33.05(26.60,41.60) |  | -61.57(-69.50,-51.11) | -1.94(-2.31,-1.56) |
| Grenada | 2.91(1.42,4.71) | 52.91(25.79,85.53) |  | 0.78(0.39,1.36) | 19.27(9.70,33.76) |  | -73.27(-87.83,-45.08) | -3.05(-3.19,-2.90) |
| Guam | 12.80(9.21,16.99) | 106.55(76.73,141.51) |  | 3.01(2.16,4.09) | 43.86(31.47,59.51) |  | -76.46(-83.74,-65.49) | -1.92(-2.18,-1.65) |
| Guatemala | 7.90(5.76,10.79) | 48.85(35.59,66.70) |  | 6.61(4.01,9.81) | 51.73(31.41,76.76) |  | -16.32(-50.01,27.83) | 1.39(0.83,1.95) |
| Guinea | 3847.90(3218.04,4588.91) | 250.84(209.78,299.14) |  | 1945.26(1430.39,2587.36) | 124.75(91.74,165.93) |  | -49.45(-65.24,-28.47) | -1.83(-2.00,-1.67) |
| Guinea-Bissau | 930.71(327.89,1582.90) | 80.20(28.25,136.39) |  | 886.64(193.47,2261.23) | 39.40(8.60,100.48) |  | -4.74(-63.82,164.23) | -1.70(-1.89,-1.50) |
| Guyana | 260.06(101.14,535.94) | 138.27(53.78,284.96) |  | 211.45(57.98,403.06) | 63.57(17.43,121.16) |  | -18.69(-71.00,146.64) | -1.84(-2.32,-1.36) |
| Haiti | 169.91(125.69,222.87) | 150.63(111.43,197.57) |  | 78.84(52.82,115.93) | 105.80(70.89,155.59) |  | -53.60(-70.48,-29.37) | 0.17(-0.41,0.75) |
| Honduras | 5993.48(940.87,14143.36) | 565.05(88.70,1333.40) |  | 5342.63(1377.38,12248.99) | 340.33(87.74,780.27) |  | -10.86(-51.93,116.69) | -1.27(-1.48,-1.06) |
| Hungary | 2328.00(1549.54,3357.54) | 282.87(188.28,407.96) |  | 947.75(365.73,2124.36) | 86.51(33.38,193.91) |  | -59.29(-85.47,-6.76) | -3.69(-3.78,-3.60) |
| Iceland | 610.54(513.76,713.61) | 98.61(82.97,115.25) |  | 118.76(83.40,167.19) | 26.14(18.35,36.79) |  | -80.55(-86.52,-71.93) | -3.91(-4.18,-3.64) |
| India | 6.93(5.29,8.84) | 32.78(25.04,41.83) |  | 4.83(3.29,6.91) | 21.98(14.98,31.44) |  | -30.25(-53.81,6.20) | -1.19(-1.74,-0.65) |
| Indonesia | 127549.67(63536.03,242062.69) | 109.73(54.66,208.24) |  | 47653.47(30806.92,70879.64) | 42.80(27.67,63.66) |  | -62.64(-82.05,-13.93) | -3.24(-3.36,-3.12) |
| Iran (Islamic Republic of) | 53545.14(18574.65,116079.53) | 239.89(83.22,520.05) |  | 28350.91(16787.92,43470.78) | 129.42(76.63,198.44) |  | -47.05(-69.91,24.62) | -1.87(-1.96,-1.78) |
| Iraq | 27122.09(16101.17,43885.10) | 309.19(183.55,500.28) |  | 2029.72(884.87,3707.50) | 32.98(14.38,60.23) |  | -92.52(-97.16,-80.25) | -5.14(-5.84,-4.43) |
| Ireland | 4690.51(2587.94,8152.71) | 149.25(82.35,259.41) |  | 3601.60(1734.33,6232.63) | 83.89(40.39,145.17) |  | -23.22(-66.77,96.55) | -1.74(-1.99,-1.49) |
| Israel | 174.76(145.75,206.69) | 60.48(50.43,71.53) |  | 44.79(34.12,61.92) | 15.01(11.43,20.75) |  | -74.37(-81.22,-63.73) | -3.79(-4.09,-3.49) |
| Italy | 481.88(394.05,583.27) | 93.32(76.31,112.96) |  | 164.77(126.38,225.38) | 17.94(13.76,24.54) |  | -65.81(-75.91,-51.57) | -4.26(-4.61,-3.91) |
| Jamaica | 2675.73(2438.38,2922.13) | 97.45(88.81,106.42) |  | 710.45(560.32,895.45) | 32.74(25.82,41.26) |  | -73.45(-79.10,-66.73) | -3.45(-3.64,-3.27) |
| Japan | 458.28(355.84,576.06) | 164.25(127.54,206.47) |  | 88.57(60.40,129.21) | 51.74(35.28,75.48) |  | -80.67(-86.85,-71.72) | -2.98(-3.32,-2.64) |
| Jordan | 4043.82(3774.39,4344.28) | 60.73(56.69,65.25) |  | 1077.16(929.53,1258.32) | 23.49(20.27,27.44) |  | -73.36(-77.29,-69.20) | -2.99(-3.22,-2.75) |
| Kazakhstan | 491.85(281.34,732.23) | 81.95(46.87,122.00) |  | 364.69(165.12,652.67) | 33.23(15.05,59.48) |  | -25.85(-68.27,54.47) | -3.09(-3.32,-2.87) |
| Kenya | 2628.31(1939.83,3277.95) | 139.59(103.02,174.09) |  | 719.28(532.33,947.52) | 36.92(27.32,48.63) |  | -72.63(-80.89,-61.85) | -3.29(-3.81,-2.76) |
| Kiribati | 3387.59(1697.36,5742.58) | 78.91(39.54,133.77) |  | 2524.42(1135.00,5907.56) | 42.43(19.08,99.28) |  | -25.48(-66.76,69.79) | -1.05(-1.47,-0.63) |
| Kuwait | 11.86(6.34,19.42) | 100.69(53.81,164.93) |  | 8.40(4.17,16.88) | 58.77(29.17,118.13) |  | -29.16(-67.19,85.97) | -1.71(-1.97,-1.46) |
| Kyrgyzstan | 188.50(141.93,239.26) | 92.20(69.42,117.03) |  | 77.41(53.50,108.83) | 29.21(20.19,41.07) |  | -58.94(-73.84,-34.40) | -3.13(-3.55,-2.70) |
| Lao People's Democratic Republic | 1117.47(856.91,1434.30) | 173.86(133.32,223.15) |  | 386.94(269.39,545.85) | 48.74(33.93,68.76) |  | -65.37(-77.67,-45.34) | -4.14(-4.54,-3.74) |
| Latvia | 2523.93(406.43,6279.35) | 355.01(57.17,883.23) |  | 1312.93(669.00,2193.49) | 158.27(80.65,264.42) |  | -47.98(-75.62,205.30) | -2.60(-2.74,-2.47) |
| Lebanon | 248.22(197.38,301.65) | 122.49(97.41,148.86) |  | 22.20(15.60,31.25) | 23.69(16.65,33.35) |  | -91.06(-94.06,-85.88) | -5.00(-5.36,-4.64) |
| Lesotho | 307.88(157.24,527.94) | 79.02(40.36,135.50) |  | 125.47(41.75,262.47) | 30.91(10.29,64.66) |  | -59.25(-87.99,24.49) | -3.39(-3.79,-2.98) |
| Liberia | 104.58(52.28,188.93) | 42.46(21.22,76.71) |  | 91.94(47.55,158.55) | 45.13(23.34,77.83) |  | -12.09(-52.05,72.87) | 0.94(0.43,1.46) |
| Libya | 633.42(228.86,1349.75) | 136.39(49.28,290.63) |  | 514.99(137.06,1054.60) | 67.20(17.88,137.61) |  | -18.70(-75.93,255.76) | -1.98(-2.69,-1.27) |
| Lithuania | 1027.63(535.48,1633.47) | 161.34(84.07,256.46) |  | 582.10(262.99,1053.66) | 137.59(62.16,249.05) |  | -43.35(-75.35,5.15) | -0.06(-0.26,0.13) |
| Luxembourg | 398.49(327.87,487.35) | 137.85(113.42,168.59) |  | 35.92(24.81,50.46) | 27.29(18.85,38.34) |  | -90.99(-94.17,-86.40) | -4.82(-5.40,-4.25) |
| Madagascar | 15.99(13.18,19.32) | 69.85(57.56,84.37) |  | 6.39(4.62,8.76) | 19.32(13.97,26.49) |  | -60.07(-72.47,-44.30) | -4.29(-4.71,-3.87) |
| Malawi | 3994.97(2294.22,6312.33) | 185.33(106.43,292.83) |  | 3950.32(1637.83,7642.41) | 96.63(40.06,186.94) |  | -1.12(-61.80,125.06) | -1.64(-1.86,-1.41) |
| Malaysia | 2374.83(1254.43,3584.05) | 125.07(66.07,188.76) |  | 1244.52(338.39,3092.10) | 45.69(12.42,113.51) |  | -47.60(-84.28,35.77) | -3.17(-3.42,-2.93) |
| Maldives | 2380.43(1129.67,4126.06) | 99.92(47.42,173.19) |  | 1007.06(563.69,1561.79) | 40.96(22.93,63.53) |  | -57.69(-81.15,11.37) | -2.11(-2.73,-1.48) |
| Mali | 87.23(22.06,196.86) | 208.78(52.80,471.17) |  | 23.25(12.42,43.07) | 73.21(39.12,135.62) |  | -73.35(-91.51,42.57) | -3.22(-3.38,-3.07) |
| Malta | 2383.01(856.71,4014.73) | 137.81(49.54,232.18) |  | 2703.67(682.97,6566.11) | 59.03(14.91,143.35) |  | 13.46(-52.25,168.92) | -2.33(-2.51,-2.15) |
| Marshall Islands | 15.14(10.93,19.22) | 53.60(38.69,68.05) |  | 5.24(3.47,7.36) | 23.83(15.78,33.43) |  | -65.36(-76.71,-47.75) | -1.98(-2.25,-1.71) |
| Mauritania | 2.68(1.58,4.32) | 36.09(21.18,58.13) |  | 1.91(0.96,3.25) | 33.74(16.96,57.24) |  | -28.68(-62.28,32.78) | -0.39(-0.97,0.20) |
| Mauritius | 295.81(130.50,493.84) | 79.74(35.18,133.13) |  | 356.02(101.89,800.87) | 54.19(15.51,121.90) |  | 20.35(-58.00,155.07) | -1.20(-1.57,-0.84) |
| Mexico | 106.68(92.74,124.30) | 101.15(87.93,117.85) |  | 28.48(22.65,34.54) | 44.29(35.23,53.73) |  | -73.30(-79.92,-65.69) | -1.23(-3.18,0.77) |
| Micronesia (Federated States of) | 38726.79(34004.92,44604.75) | 328.25(288.23,378.07) |  | 12342.83(8667.43,17340.42) | 124.96(87.75,175.56) |  | -68.13(-77.55,-53.82) | -2.81(-3.24,-2.37) |
| Monaco | 9.73(5.14,15.97) | 62.12(32.82,101.96) |  | 2.53(1.31,4.61) | 26.72(13.80,48.72) |  | -73.99(-88.19,-34.81) | -2.62(-2.82,-2.42) |
| Mongolia | 2.58(1.20,4.58) | 220.09(102.14,390.98) |  | 2.21(1.01,3.74) | 136.30(62.26,230.89) |  | -14.42(-59.80,81.88) | -3.37(-4.19,-2.54) |
| Montenegro | 799.20(426.30,1451.29) | 235.39(125.56,427.44) |  | 199.76(99.79,335.38) | 51.13(25.54,85.84) |  | -75.01(-90.38,-40.65) | -4.87(-5.23,-4.51) |
| Morocco | 50.86(30.33,79.69) | 96.94(57.82,151.89) |  | 6.71(3.17,11.33) | 18.53(8.76,31.30) |  | -86.82(-94.23,-72.75) | -3.91(-4.38,-3.44) |
| Mozambique | 1736.85(801.70,3465.87) | 48.78(22.52,97.35) |  | 554.45(203.16,1165.78) | 17.06(6.25,35.86) |  | -68.08(-88.80,4.80) | -2.92(-3.16,-2.69) |
| Myanmar | 15178.59(7775.78,25238.79) | 630.08(322.78,1047.69) |  | 13787.40(3792.07,33774.29) | 266.21(73.22,652.11) |  | -9.17(-74.49,164.62) | -2.46(-2.64,-2.27) |
| Namibia | 23673.08(4009.84,57052.05) | 469.50(79.53,1131.49) |  | 11419.33(5656.17,20144.98) | 218.49(108.22,385.45) |  | -51.76(-77.59,149.89) | -2.62(-2.77,-2.46) |
| Nauru | 127.14(57.34,241.59) | 56.17(25.33,106.73) |  | 147.41(72.23,254.63) | 52.94(25.94,91.44) |  | 15.94(-48.09,197.40) | 0.78(0.08,1.49) |
| Nepal | 1.21(0.70,1.97) | 74.15(43.11,120.36) |  | 0.85(0.49,1.34) | 60.66(34.79,95.63) |  | -30.02(-60.50,19.31) | -0.75(-1.38,-0.12) |
| Netherlands | 5246.49(1488.59,12271.67) | 159.30(45.20,372.62) |  | 1396.40(731.07,2234.75) | 44.95(23.53,71.94) |  | -73.38(-90.90,7.70) | -3.83(-4.00,-3.66) |
| New Zealand | 478.18(397.72,571.74) | 51.07(42.48,61.06) |  | 117.38(86.24,161.87) | 13.63(10.01,18.80) |  | -75.45(-82.55,-65.06) | -4.04(-4.34,-3.74) |
| Nicaragua | 186.96(163.72,213.70) | 66.86(58.55,76.42) |  | 79.02(63.69,95.99) | 25.28(20.37,30.71) |  | -57.73(-66.41,-45.83) | -2.46(-2.92,-2.00) |
| Niger | 2812.44(1847.94,4032.25) | 423.87(278.51,607.72) |  | 577.25(296.86,1021.98) | 88.73(45.63,157.10) |  | -79.48(-90.80,-55.87) | -4.64(-4.84,-4.44) |
| Nigeria | 2867.87(1155.57,6045.57) | 170.00(68.50,358.36) |  | 4517.08(1181.31,8656.05) | 88.63(23.18,169.85) |  | 57.51(-44.65,403.50) | -1.85(-2.07,-1.64) |
| Niue | 24976.11(8623.47,49724.72) | 157.13(54.25,312.83) |  | 46554.03(11066.38,73519.40) | 125.42(29.81,198.07) |  | 86.39(-5.69,346.71) | -0.46(-0.57,-0.35) |
| North Macedonia | 0.12(0.07,0.20) | 47.17(27.23,77.29) |  | 0.20(0.12,0.36) | 172.00(101.85,302.33) |  | 67.91(0.53,195.33) | 0.87(-0.29,2.04) |
| Northern Mariana Islands | 282.23(176.23,420.84) | 165.69(103.46,247.06) |  | 20.03(8.44,38.23) | 19.92(8.40,38.03) |  | -92.90(-97.53,-82.76) | -4.68(-5.27,-4.08) |
| Norway | 1.06(0.58,1.72) | 22.23(12.23,36.20) |  | 0.38(0.21,0.58) | 11.82(6.60,18.15) |  | -64.11(-77.95,-42.56) | -1.64(-1.96,-1.33) |
| Oman | 108.58(96.51,119.93) | 39.36(34.99,43.48) |  | 44.34(35.99,56.44) | 15.77(12.80,20.08) |  | -59.16(-67.42,-48.44) | -2.46(-2.73,-2.19) |
| Pakistan | 89.00(34.71,174.03) | 27.16(10.59,53.11) |  | 32.20(13.31,56.00) | 7.59(3.14,13.21) |  | -63.82(-86.65,8.47) | -2.97(-3.83,-2.10) |
| Palau | 23854.26(9921.74,47185.46) | 129.21(53.74,255.59) |  | 28604.66(15016.32,46776.76) | 96.23(50.52,157.36) |  | 19.91(-29.06,137.59) | -0.20(-0.54,0.15) |
| Palestine | 0.24(0.02,1.19) | 16.16(1.28,80.47) |  | 0.08(0.01,0.40) | 8.74(0.77,42.55) |  | -65.34(-88.52,8.88) | -1.74(-1.99,-1.48) |
| Panama | 767.16(348.26,1355.94) | 196.71(89.30,347.68) |  | 478.00(290.46,737.01) | 78.01(47.40,120.28) |  | -37.69(-71.04,49.28) | -2.49(-2.79,-2.18) |
| Papua New Guinea | 533.85(426.47,657.48) | 186.95(149.35,230.25) |  | 430.95(325.54,567.79) | 116.09(87.69,152.95) |  | -19.27(-42.71,15.07) | -1.38(-1.54,-1.22) |
| Paraguay | 636.50(219.23,1314.44) | 98.27(33.85,202.94) |  | 1250.01(514.57,2445.43) | 82.17(33.82,160.74) |  | 96.39(24.49,264.68) | -0.50(-0.86,-0.14) |
| Peru | 1299.37(888.36,1847.66) | 210.59(143.98,299.45) |  | 805.46(458.41,1418.20) | 123.90(70.52,218.16) |  | -38.01(-67.41,16.40) | -0.93(-1.31,-0.54) |
| Philippines | 9357.75(5951.52,15768.39) | 319.82(203.40,538.91) |  | 3371.29(1372.64,5967.60) | 102.14(41.59,180.81) |  | -63.97(-89.31,-18.26) | -2.98(-3.34,-2.62) |
| Poland | 22535.55(11592.26,39943.80) | 243.60(125.31,431.77) |  | 15263.09(9671.55,21019.01) | 136.11(86.25,187.45) |  | -32.27(-66.07,56.53) | -1.02(-1.32,-0.72) |
| Portugal | 3031.84(2164.04,3822.59) | 103.87(74.14,130.96) |  | 537.78(420.04,674.24) | 28.57(22.32,35.82) |  | -82.26(-87.90,-72.07) | -4.64(-5.02,-4.26) |
| Puerto Rico | 665.33(556.96,793.89) | 115.03(96.29,137.25) |  | 129.43(96.63,173.56) | 30.43(22.72,40.81) |  | -80.55(-86.13,-73.50) | -4.17(-4.91,-3.42) |
| Qatar | 259.61(208.95,316.15) | 81.33(65.46,99.05) |  | 28.83(21.53,38.45) | 27.42(20.48,36.58) |  | -88.90(-92.44,-83.41) | -2.54(-2.88,-2.21) |
| Republic of Korea | 18.00(10.07,30.79) | 35.42(19.82,60.60) |  | 19.79(9.11,41.38) | 10.74(4.94,22.45) |  | 9.95(-56.60,136.68) | -3.61(-3.78,-3.45) |
| Republic of Moldova | 2402.38(1418.15,3474.05) | 72.38(42.73,104.67) |  | 291.80(101.92,547.53) | 18.83(6.58,35.33) |  | -87.85(-95.66,-72.51) | -3.55(-3.77,-3.34) |
| Romania | 1302.81(1059.78,1572.44) | 302.38(245.97,364.96) |  | 92.81(66.23,126.81) | 60.18(42.95,82.22) |  | -92.88(-95.13,-89.50) | -4.68(-5.16,-4.20) |
| Russian Federation | 3206.73(2677.26,3845.63) | 181.22(151.30,217.32) |  | 325.21(253.44,423.51) | 34.68(27.02,45.16) |  | -89.86(-92.50,-85.86) | -5.00(-5.31,-4.68) |
| Rwanda | 17837.89(16574.66,19032.17) | 153.45(142.59,163.73) |  | 3105.04(2633.23,3621.52) | 40.80(34.60,47.58) |  | -82.59(-84.98,-79.98) | -5.14(-5.88,-4.39) |
| Saint Kitts and Nevis | 2999.13(1671.60,5011.36) | 222.51(124.02,371.80) |  | 1968.45(705.37,4013.23) | 112.58(40.34,229.53) |  | -34.37(-79.65,87.54) | -2.39(-2.78,-1.99) |
| Saint Lucia | 7.54(6.09,9.20) | 160.99(129.89,196.32) |  | 2.17(1.48,3.07) | 71.20(48.49,100.55) |  | -71.20(-80.68,-56.97) | -1.74(-2.02,-1.46) |
| Saint Vincent and the Grenadines | 20.61(15.58,26.33) | 116.74(88.23,149.13) |  | 3.41(2.23,5.19) | 38.61(25.29,58.77) |  | -83.45(-89.98,-72.85) | -2.70(-2.94,-2.46) |
| Samoa | 14.04(10.76,18.68) | 109.85(84.24,146.21) |  | 2.85(2.01,4.01) | 39.61(27.98,55.76) |  | -79.69(-86.84,-69.82) | -2.94(-3.27,-2.60) |
| San Marino | 17.48(10.15,29.46) | 68.59(39.83,115.58) |  | 10.94(4.92,22.97) | 37.42(16.84,78.54) |  | -37.41(-70.01,46.70) | -1.85(-1.97,-1.72) |
| Sao Tome and Principe | 3.00(1.70,4.83) | 253.09(143.66,406.64) |  | 0.93(0.49,1.58) | 76.50(40.21,131.02) |  | -69.20(-85.82,-28.97) | -3.12(-3.32,-2.91) |
| Saudi Arabia | 16.43(8.31,28.04) | 80.23(40.59,136.91) |  | 4.33(0.85,12.33) | 17.37(3.41,49.42) |  | -73.64(-94.98,-18.70) | -4.71(-5.39,-4.02) |
| Senegal | 2728.43(1562.03,4479.05) | 112.85(64.61,185.26) |  | 461.64(205.96,940.13) | 18.97(8.46,38.64) |  | -83.08(-93.43,-57.21) | -5.71(-5.83,-5.59) |
| Serbia | 1951.62(844.20,3404.65) | 133.21(57.62,232.39) |  | 1426.00(375.96,3428.01) | 62.78(16.55,150.92) |  | -26.93(-78.13,124.22) | -1.90(-2.27,-1.54) |
| Seychelles | 736.73(384.08,1217.65) | 108.23(56.42,178.88) |  | 56.17(26.42,93.34) | 15.24(7.17,25.32) |  | -92.38(-96.97,-81.09) | -6.76(-7.45,-6.07) |
| Sierra Leone | 4.25(2.39,7.18) | 52.70(29.68,89.14) |  | 2.87(1.57,4.70) | 36.42(19.91,59.68) |  | -32.47(-65.87,26.24) | -0.65(-0.83,-0.46) |
| Singapore | 1198.05(460.35,2431.70) | 154.83(59.49,314.26) |  | 1353.21(359.35,2569.19) | 100.76(26.76,191.31) |  | 12.95(-56.27,250.99) | -1.11(-1.31,-0.92) |
| Slovakia | 176.20(140.87,216.25) | 86.35(69.04,105.98) |  | 63.45(48.11,81.39) | 22.19(16.82,28.47) |  | -63.99(-74.29,-49.53) | -3.75(-4.31,-3.18) |
| Slovenia | 263.82(176.27,366.69) | 64.48(43.08,89.62) |  | 78.83(40.80,133.09) | 27.55(14.26,46.51) |  | -70.12(-85.52,-42.96) | -2.46(-2.60,-2.32) |
| Solomon Islands | 75.56(61.04,90.56) | 61.43(49.63,73.63) |  | 17.29(12.07,24.25) | 17.66(12.33,24.77) |  | -77.11(-83.95,-66.19) | -4.28(-4.65,-3.92) |
| Somalia | 35.61(19.10,59.54) | 59.70(32.01,99.81) |  | 36.00(18.35,67.60) | 37.76(19.25,70.91) |  | 1.09(-47.11,137.80) | -1.43(-1.76,-1.10) |
| South Africa | 2958.04(1221.90,6003.73) | 191.45(79.09,388.58) |  | 4077.47(1893.73,7443.36) | 98.75(45.86,180.27) |  | 37.84(-34.19,214.35) | -1.40(-1.89,-0.91) |
| South Sudan | 3130.80(1407.08,6060.91) | 64.18(28.84,124.24) |  | 1950.95(1290.05,2738.17) | 39.32(26.00,55.19) |  | -37.69(-64.63,43.51) | -0.56(-1.43,0.32) |
| Spain | 2515.09(1186.63,4837.46) | 247.11(116.59,475.28) |  | 3169.72(1569.50,5928.60) | 202.88(100.46,379.47) |  | 26.03(-23.78,124.18) | 0.07(-0.55,0.69) |
| Sri Lanka | 1773.45(1512.27,2066.51) | 85.15(72.61,99.22) |  | 529.72(404.83,694.93) | 28.77(21.99,37.74) |  | -70.13(-78.53,-59.72) | -3.58(-3.76,-3.39) |
| Sudan | 2318.15(1580.07,3372.37) | 131.13(89.38,190.76) |  | 480.59(237.17,883.27) | 30.70(15.15,56.42) |  | -79.27(-90.20,-57.37) | -4.38(-4.91,-3.84) |
| Suriname | 13789.10(2946.72,33655.40) | 396.14(84.65,966.86) |  | 9151.96(3813.49,17515.88) | 162.30(67.63,310.62) |  | -33.63(-69.23,185.23) | -2.51(-2.75,-2.26) |
| Sweden | 53.62(26.58,75.75) | 122.40(60.69,172.94) |  | 33.69(19.50,51.96) | 75.65(43.79,116.67) |  | -37.16(-63.63,21.43) | -1.58(-1.73,-1.42) |
| Switzerland | 443.49(378.79,533.67) | 78.76(67.27,94.78) |  | 118.36(93.87,151.65) | 20.30(16.10,26.00) |  | -73.31(-79.89,-64.68) | -4.36(-5.12,-3.59) |
| Syrian Arab Republic | 232.91(187.62,282.17) | 58.47(47.10,70.84) |  | 99.86(71.52,137.99) | 22.59(16.18,31.22) |  | -57.13(-71.43,-37.38) | -3.51(-4.00,-3.02) |
| Taiwan (Province of China) | 4115.38(1935.19,7356.77) | 190.80(89.72,341.07) |  | 595.61(293.06,1058.67) | 59.26(29.16,105.33) |  | -85.53(-91.72,-74.06) | -3.27(-3.87,-2.66) |
| Tajikistan | 485.38(318.77,694.10) | 30.21(19.84,43.21) |  | 209.86(156.45,279.84) | 23.52(17.53,31.36) |  | -56.76(-74.08,-25.70) | 0.00(-0.50,0.51) |
| Thailand | 2769.36(1391.57,4116.04) | 292.89(147.17,435.31) |  | 1676.16(820.40,3344.72) | 125.23(61.29,249.89) |  | -39.47(-71.51,22.77) | -2.71(-2.99,-2.43) |
| Timor-Leste | 5737.33(3283.82,9455.59) | 110.33(63.15,181.83) |  | 1594.10(910.72,2409.44) | 56.39(32.21,85.23) |  | -72.22(-86.21,-48.18) | -1.95(-2.14,-1.76) |
| Togo | 523.59(92.44,1238.24) | 373.86(66.00,884.14) |  | 333.50(163.82,593.86) | 180.42(88.62,321.27) |  | -36.30(-69.16,195.37) | -2.67(-3.00,-2.34) |
| Tokelau | 687.84(286.35,1184.84) | 100.22(41.72,172.64) |  | 803.64(194.28,1672.66) | 68.54(16.57,142.66) |  | 16.84(-56.42,189.47) | -0.93(-1.08,-0.78) |
| Tonga | 0.11(0.06,0.20) | 59.04(29.46,101.40) |  | 0.24(0.07,0.60) | 242.14(72.69,609.84) |  | 108.79(-38.99,470.13) | -0.33(-2.26,1.64) |
| Trinidad and Tobago | 4.56(2.53,7.94) | 29.85(16.54,51.98) |  | 3.19(1.75,5.81) | 22.13(12.11,40.34) |  | -30.07(-68.73,54.08) | -1.06(-1.40,-0.72) |
| Tunisia | 135.62(111.68,164.28) | 101.73(83.77,123.23) |  | 40.14(26.82,56.74) | 49.86(33.31,70.47) |  | -70.40(-80.96,-55.06) | -2.44(-2.92,-1.96) |
| Turkey | 1438.42(743.21,2389.83) | 134.79(69.64,223.94) |  | 289.13(105.22,565.50) | 32.41(11.80,63.39) |  | -79.90(-92.78,-48.22) | -4.28(-4.38,-4.18) |
| Turkmenistan | 21772.73(10875.54,37718.62) | 313.05(156.37,542.32) |  | 3548.50(2075.00,5529.56) | 63.92(37.37,99.60) |  | -83.70(-93.15,-60.43) | -5.03(-5.42,-4.62) |
| Tuvalu | 931.09(626.21,1377.74) | 159.00(106.94,235.27) |  | 305.53(219.07,441.80) | 56.61(40.59,81.86) |  | -67.19(-78.17,-46.79) | -3.10(-3.41,-2.79) |
| Uganda | 1.25(0.37,2.56) | 83.05(24.62,169.79) |  | 0.37(0.22,0.58) | 29.08(17.27,44.97) |  | -70.22(-86.81,14.60) | -3.14(-3.27,-3.00) |
| Ukraine | 5284.45(2742.43,8476.17) | 147.14(76.36,236.01) |  | 6884.61(2225.34,14673.32) | 94.08(30.41,200.52) |  | 30.28(-49.40,188.80) | -1.02(-1.28,-0.76) |
| United Arab Emirates | 9097.90(6909.96,11484.82) | 241.39(183.34,304.72) |  | 1107.82(796.22,1604.10) | 69.58(50.01,100.75) |  | -87.82(-91.87,-81.48) | -3.67(-4.05,-3.28) |
| United Kingdom | 158.73(85.38,287.92) | 69.63(37.46,126.31) |  | 81.64(33.25,149.18) | 18.87(7.69,34.48) |  | -48.57(-81.46,35.69) | -2.77(-3.26,-2.27) |
| United Republic of Tanzania | 2454.80(2251.38,2674.68) | 63.90(58.61,69.63) |  | 755.41(656.22,867.27) | 20.67(17.96,23.73) |  | -69.23(-73.59,-64.26) | -2.94(-3.33,-2.55) |
| United States of America | 11441.15(6867.57,17262.59) | 237.96(142.84,359.04) |  | 13047.75(5331.50,24609.70) | 147.50(60.27,278.20) |  | 14.04(-55.85,161.30) | -0.84(-1.11,-0.56) |
| United States Virgin Islands | 11930.32(11410.89,12528.63) | 60.40(57.77,63.43) |  | 4840.65(4272.97,5540.95) | 26.03(22.98,29.80) |  | -59.43(-64.21,-53.91) | -2.12(-2.30,-1.94) |
| Uruguay | 6.70(3.69,10.28) | 60.87(33.56,93.38) |  | 1.37(0.51,2.83) | 34.87(12.96,72.21) |  | -79.60(-92.04,-52.72) | -1.34(-1.49,-1.20) |
| Uzbekistan | 232.00(193.57,277.17) | 85.03(70.95,101.59) |  | 58.41(41.69,82.89) | 30.11(21.49,42.72) |  | -74.82(-82.45,-63.16) | -3.23(-3.49,-2.97) |
| Vanuatu | 5957.57(3929.14,8803.98) | 176.63(116.49,261.01) |  | 4107.85(2704.64,6170.82) | 107.11(70.52,160.90) |  | -31.05(-60.25,15.64) | -1.41(-1.75,-1.08) |
| Venezuela (Bolivarian Republic of) | 10.71(5.82,17.18) | 39.62(21.51,63.55) |  | 11.52(6.58,18.89) | 27.34(15.60,44.81) |  | 7.57(-37.85,104.88) | -1.04(-1.62,-0.44) |
| Viet Nam | 4191.48(3650.37,4731.12) | 165.62(144.24,186.94) |  | 2553.52(1433.01,3882.68) | 116.87(65.59,177.70) |  | -39.08(-66.18,-6.77) | -0.72(-1.13,-0.31) |
| Yemen | 13412.82(7033.40,24001.45) | 142.55(74.75,255.08) |  | 5226.34(2826.25,8487.68) | 64.19(34.71,104.25) |  | -61.03(-81.46,-15.02) | -2.46(-2.62,-2.30) |
| Zambia | 7335.14(2028.92,17927.24) | 264.18(73.07,645.66) |  | 5908.62(2489.02,10591.81) | 125.79(52.99,225.49) |  | -19.45(-60.12,203.76) | -2.22(-2.37,-2.06) |
| Zimbabwe | 3959.84(2207.90,6689.89) | 262.39(146.30,443.28) |  | 3433.89(1392.79,6997.41) | 117.40(47.62,239.22) |  | -13.28(-69.93,139.67) | -2.18(-2.37,-1.99) |
