# Supplementary material for: Analysis of global trends in acute lymphoblastic leukemia in children aged 0–5 years from 1990 to 2021
Source: Front Pediatr. 2025 Mar 13;13:1542649. doi: 10.3389/fped.2025.1542649 (PMC11966407; doi:10.3389/fped.2025.1542649)
Supplement: Supplementary file 4 [file Table3.docx]

**Table S3.** National Prevalence of Acute Lymphoblastic Leukemia in Children Aged 0–5 Years from 1990 to 2021.

| location | 1990 | |  | 2021 | |  | 1990-2021 | |
| --- | --- | --- | --- | --- | --- | --- | --- | --- |
|  | Prevalence case | Prevalence rate |  | Prevalence case | Prevalence rate |  | Cases change | EAPC |
| Afghanistan | 82.28(11.92,191.78) | 4.80(0.70,11.19) |  | 236.96(104.98,491.18) | 4.32(1.92,8.96) |  | 188.01(46.86,1313.99) | -0.09(-0.38,0.21) |
| Albania | 36.62(15.61,65.02) | 9.07(3.87,16.10) |  | 60.44(29.91,105.84) | 42.39(20.98,74.22) |  | 65.04(-27.77,309.57) | 5.67(5.17,6.17) |
| Algeria | 98.64(46.21,181.75) | 2.64(1.24,4.86) |  | 315.76(146.00,555.23) | 6.71(3.10,11.80) |  | 220.11(22.88,735.74) | 3.50(3.35,3.65) |
| American Samoa | 0.17(0.09,0.29) | 2.18(1.17,3.86) |  | 0.09(0.04,0.18) | 2.46(1.09,4.90) |  | -45.07(-78.91,41.38) | 0.03(-0.33,0.38) |
| Andorra | 5.87(2.08,12.59) | 217.03(76.66,464.98) |  | 2.34(1.26,3.72) | 92.76(49.72,147.52) |  | -60.14(-83.85,31.98) | -2.28(-2.82,-1.74) |
| Angola | 49.52(8.21,125.90) | 2.54(0.42,6.45) |  | 94.28(44.58,191.22) | 1.67(0.79,3.39) |  | 90.39(-8.16,739.49) | -1.22(-1.33,-1.12) |
| Antigua and Barbuda | 0.42(0.28,0.61) | 6.89(4.57,10.06) |  | 0.89(0.55,1.33) | 16.82(10.46,25.19) |  | 112.27(22.01,260.51) | 3.60(2.94,4.26) |
| Argentina | 354.93(262.62,489.87) | 10.33(7.64,14.25) |  | 557.80(356.70,865.92) | 18.62(11.90,28.90) |  | 57.16(-6.67,173.21) | 2.15(1.72,2.59) |
| Armenia | 79.11(55.87,107.31) | 20.66(14.59,28.02) |  | 39.56(23.83,64.55) | 21.24(12.80,34.67) |  | -49.99(-71.85,-13.32) | 0.89(0.32,1.46) |
| Australia | 508.32(362.41,716.08) | 40.26(28.70,56.71) |  | 752.89(511.80,1025.64) | 50.08(34.04,68.22) |  | 48.11(-10.58,140.51) | 1.65(1.00,2.30) |
| Austria | 296.18(195.65,432.54) | 66.25(43.77,96.76) |  | 227.04(166.43,302.93) | 52.54(38.52,70.10) |  | -23.34(-53.57,29.15) | -0.76(-1.15,-0.37) |
| Azerbaijan | 88.86(49.25,140.39) | 9.83(5.45,15.53) |  | 129.53(60.65,248.42) | 17.96(8.41,34.45) |  | 45.77(-35.01,215.70) | 2.75(2.18,3.33) |
| Bahamas | 1.06(0.72,1.50) | 4.17(2.83,5.87) |  | 0.88(0.50,1.45) | 4.19(2.37,6.88) |  | -17.05(-55.91,43.18) | 1.08(0.46,1.70) |
| Bahrain | 2.51(1.32,4.56) | 4.08(2.15,7.42) |  | 10.77(5.42,19.83) | 11.50(5.78,21.17) |  | 329.83(108.25,804.62) | 4.58(4.13,5.03) |
| Bangladesh | 651.55(183.52,1440.44) | 3.44(0.97,7.61) |  | 576.17(312.66,983.89) | 4.01(2.18,6.85) |  | -11.57(-71.29,256.61) | 0.59(0.12,1.06) |
| Barbados | 1.21(0.78,1.71) | 6.15(4.01,8.74) |  | 0.99(0.58,1.68) | 7.29(4.23,12.32) |  | -17.65(-57.71,55.60) | 0.25(-0.36,0.86) |
| Belarus | 200.46(133.73,289.54) | 24.88(16.60,35.93) |  | 115.74(60.20,212.34) | 24.76(12.88,45.43) |  | -42.26(-71.56,12.37) | 0.65(-0.24,1.54) |
| Belgium | 578.25(367.92,878.60) | 96.94(61.68,147.29) |  | 387.12(273.09,530.22) | 65.40(46.13,89.57) |  | -33.05(-60.16,11.48) | -1.18(-1.56,-0.81) |
| Belize | 3.30(2.43,4.52) | 11.18(8.22,15.29) |  | 2.61(1.72,3.79) | 6.86(4.52,9.93) |  | -20.81(-52.73,28.85) | -0.78(-1.17,-0.39) |
| Benin | 25.06(11.30,43.08) | 2.54(1.15,4.37) |  | 71.14(19.69,135.07) | 3.04(0.84,5.77) |  | 183.91(9.96,571.29) | 0.90(0.71,1.09) |
| Bermuda | 0.49(0.23,0.89) | 11.31(5.45,20.73) |  | 1.33(0.62,2.53) | 51.99(24.26,99.25) |  | 172.90(2.56,662.72) | 5.40(4.84,5.97) |
| Bhutan | 2.62(0.83,5.48) | 2.74(0.87,5.74) |  | 2.94(1.52,5.31) | 4.82(2.49,8.71) |  | 12.33(-61.72,293.62) | 1.36(1.02,1.69) |
| Bolivia (Plurinational State of) | 105.19(47.49,193.70) | 10.41(4.70,19.18) |  | 146.21(75.28,241.24) | 12.24(6.30,20.20) |  | 39.00(-45.94,311.02) | 0.64(0.51,0.77) |
| Bosnia and Herzegovina | 17.14(8.81,28.22) | 4.87(2.51,8.03) |  | 12.97(5.86,24.91) | 8.59(3.88,16.49) |  | -24.29(-68.77,67.75) | 3.41(2.40,4.43) |
| Botswana | 2.22(1.04,4.14) | 1.04(0.49,1.95) |  | 6.08(2.81,11.92) | 2.58(1.19,5.06) |  | 174.31(41.21,477.06) | 4.14(3.20,5.07) |
| Brazil | 979.21(807.72,1162.39) | 5.95(4.91,7.06) |  | 1642.72(1226.18,2145.99) | 9.92(7.41,12.96) |  | 67.76(21.84,129.10) | 2.28(1.92,2.64) |
| Brunei Darussalam | 1.22(0.63,2.04) | 3.55(1.83,5.91) |  | 2.52(1.24,4.29) | 8.17(4.01,13.90) |  | 106.22(-8.71,364.14) | 2.87(2.63,3.10) |
| Bulgaria | 80.98(52.18,117.69) | 15.08(9.72,21.92) |  | 27.35(15.25,45.77) | 9.04(5.04,15.12) |  | -66.23(-83.16,-33.49) | -1.12(-1.65,-0.59) |
| Burkina Faso | 46.12(19.69,87.47) | 2.46(1.05,4.66) |  | 108.45(31.12,203.97) | 2.64(0.76,4.97) |  | 135.16(3.10,428.44) | 0.80(0.60,0.99) |
| Burundi | 39.11(21.67,64.85) | 3.64(2.02,6.03) |  | 40.70(10.50,92.12) | 1.88(0.49,4.26) |  | 4.07(-70.16,199.02) | -1.06(-1.54,-0.58) |
| Cabo Verde | 2.91(1.49,5.17) | 4.89(2.50,8.68) |  | 3.46(1.00,11.98) | 7.86(2.27,27.19) |  | 19.06(-68.91,285.04) | 2.15(1.58,2.73) |
| Cambodia | 111.21(31.01,247.81) | 6.09(1.70,13.58) |  | 125.94(73.49,205.88) | 7.20(4.20,11.77) |  | 13.25(-49.82,391.19) | 0.27(-0.04,0.59) |
| Cameroon | 41.89(18.64,69.45) | 2.09(0.93,3.46) |  | 124.90(38.78,231.91) | 2.57(0.80,4.77) |  | 198.14(35.48,521.79) | 1.13(0.83,1.44) |
| Canada | 1874.37(1196.16,2703.18) | 97.37(62.14,140.42) |  | 999.11(778.60,1283.41) | 52.58(40.98,67.54) |  | -46.70(-66.43,-12.80) | -1.10(-1.61,-0.58) |
| Central African Republic | 10.90(2.60,27.03) | 2.16(0.52,5.37) |  | 12.61(4.11,29.86) | 1.50(0.49,3.56) |  | 15.64(-31.36,162.62) | -0.91(-1.06,-0.76) |
| Chad | 24.04(9.24,46.00) | 1.93(0.74,3.70) |  | 81.09(26.15,163.39) | 2.23(0.72,4.49) |  | 237.36(71.16,666.70) | 0.91(0.75,1.06) |
| Chile | 154.16(112.23,212.07) | 10.73(7.81,14.76) |  | 315.24(197.15,512.51) | 28.98(18.12,47.11) |  | 104.49(16.09,260.21) | 4.17(3.59,4.75) |
| China | 36414.67(24135.77,52451.79) | 32.57(21.59,46.91) |  | 98215.59(47784.03,156575.12) | 126.46(61.52,201.59) |  | 169.71(17.57,404.73) | 5.87(5.31,6.43) |
| Colombia | 399.09(300.04,536.49) | 9.49(7.13,12.75) |  | 1008.95(590.72,1642.94) | 29.31(17.16,47.72) |  | 152.81(43.83,347.39) | 6.20(5.21,7.20) |
| Comoros | 2.80(1.57,4.25) | 3.40(1.91,5.16) |  | 2.65(1.04,5.27) | 3.26(1.28,6.48) |  | -5.33(-60.82,106.56) | 0.07(-0.31,0.46) |
| Congo | 6.89(2.42,14.99) | 1.75(0.61,3.80) |  | 8.12(4.08,14.44) | 1.28(0.64,2.28) |  | 17.86(-44.97,270.82) | -0.96(-1.13,-0.79) |
| Cook Islands | 0.03(0.02,0.06) | 1.47(0.73,2.54) |  | 0.05(0.01,0.14) | 4.75(1.07,12.45) |  | 61.75(-70.34,477.20) | 0.47(-0.77,1.72) |
| Costa Rica | 84.97(59.28,117.53) | 20.93(14.60,28.95) |  | 125.19(79.98,189.92) | 40.61(25.94,61.60) |  | 47.33(-14.89,149.99) | 2.72(2.52,2.92) |
| Croatia | 32.27(11.38,54.18) | 1.40(0.49,2.35) |  | 68.88(19.36,163.97) | 1.58(0.44,3.77) |  | 36.44(-42.24,218.77) | 0.61(0.19,1.03) |
| Cuba | 50.51(27.69,87.31) | 16.76(9.19,28.98) |  | 68.91(32.36,131.65) | 37.81(17.76,72.23) |  | -30.92(-63.32,22.27) | 2.98(2.58,3.39) |
| Cyprus | 176.11(123.28,242.31) | 19.68(13.77,27.07) |  | 121.65(72.47,194.27) | 22.34(13.31,35.68) |  | 116.56(-26.91,490.04) | 1.23(0.75,1.72) |
| Czechia | 21.04(10.56,38.64) | 32.93(16.52,60.46) |  | 45.57(21.97,81.09) | 60.72(29.28,108.06) |  | 8.41(-48.13,120.32) | 2.94(2.08,3.81) |
| C么te d'Ivoire | 140.03(89.01,208.38) | 21.58(13.72,32.12) |  | 151.81(78.06,270.39) | 27.01(13.89,48.11) |  | 113.45(-10.13,394.32) | 1.16(0.62,1.71) |
| Democratic People's Republic of Korea | 317.14(156.57,525.23) | 13.57(6.70,22.48) |  | 370.37(147.41,820.20) | 24.48(9.74,54.21) |  | 16.79(-49.55,155.59) | 2.79(2.33,3.25) |
| Democratic Republic of the Congo | 141.85(38.70,339.36) | 1.94(0.53,4.65) |  | 151.85(66.88,306.58) | 1.12(0.49,2.26) |  | 7.05(-42.69,248.55) | -1.27(-1.50,-1.03) |
| Denmark | 110.17(73.50,157.14) | 38.04(25.38,54.26) |  | 177.75(120.73,243.94) | 57.21(38.86,78.51) |  | 61.34(-1.85,164.34) | 1.59(1.25,1.94) |
| Djibouti | 2.29(1.03,4.16) | 3.54(1.59,6.44) |  | 4.26(1.37,10.48) | 2.93(0.94,7.19) |  | 86.49(-17.69,274.94) | -0.38(-0.68,-0.07) |
| Dominica | 0.52(0.33,0.76) | 5.96(3.79,8.83) |  | 0.61(0.35,0.99) | 17.66(9.99,28.45) |  | 19.03(-39.87,119.60) | 3.62(3.26,3.98) |
| Dominican Republic | 92.50(51.13,147.49) | 9.29(5.13,14.81) |  | 81.36(31.72,182.61) | 7.88(3.07,17.68) |  | -12.04(-69.08,120.97) | -0.55(-0.89,-0.21) |
| Ecuador | 69.24(54.66,90.20) | 5.15(4.06,6.70) |  | 340.08(200.90,545.78) | 20.47(12.09,32.86) |  | 391.18(161.80,741.03) | 4.78(4.01,5.56) |
| Egypt | 348.58(189.45,737.52) | 4.09(2.22,8.64) |  | 1649.67(779.14,2762.27) | 12.65(5.98,21.19) |  | 373.25(48.12,938.42) | 4.36(3.97,4.74) |
| El Salvador | 68.87(44.46,100.32) | 8.94(5.77,13.03) |  | 98.00(44.03,179.10) | 16.31(7.33,29.81) |  | 42.31(-45.57,244.97) | 2.36(1.76,2.96) |
| Equatorial Guinea | 1.47(0.38,3.56) | 1.79(0.46,4.33) |  | 4.13(1.39,8.15) | 2.20(0.74,4.35) |  | 180.63(3.55,1226.72) | 0.40(0.02,0.79) |
| Eritrea | 19.14(8.99,35.79) | 3.07(1.44,5.74) |  | 27.39(11.00,56.58) | 2.98(1.20,6.16) |  | 43.14(-52.01,331.91) | -0.12(-0.35,0.11) |
| Estonia | 27.15(18.15,38.83) | 22.51(15.05,32.20) |  | 14.30(8.75,22.78) | 20.68(12.65,32.93) |  | -47.32(-72.44,-5.50) | 0.95(0.17,1.75) |
| Eswatini | 2.00(0.74,4.23) | 1.37(0.51,2.91) |  | 2.11(1.21,3.49) | 1.50(0.86,2.49) |  | 5.70(-48.20,187.65) | 0.48(-0.34,1.31) |
| Ethiopia | 846.55(129.49,1863.80) | 8.78(1.34,19.34) |  | 1182.01(549.68,2678.09) | 7.40(3.44,16.77) |  | 39.63(-38.62,527.70) | -0.92(-1.43,-0.40) |
| Fiji | 1.70(0.46,3.47) | 1.81(0.49,3.68) |  | 2.81(0.71,5.87) | 3.08(0.78,6.45) |  | 64.81(-19.48,226.13) | 1.59(1.42,1.75) |
| Finland | 65.06(43.88,93.23) | 20.81(14.03,29.82) |  | 105.10(73.72,145.60) | 43.29(30.36,59.97) |  | 61.55(-4.01,165.54) | 2.78(2.08,3.49) |
| France | 2697.36(1766.76,4020.19) | 69.35(45.43,103.36) |  | 3099.49(2340.02,3939.71) | 87.71(66.22,111.48) |  | 14.91(-29.99,85.95) | 1.46(1.19,1.73) |
| Gabon | 2.06(0.86,3.99) | 1.32(0.55,2.55) |  | 4.13(1.72,7.69) | 1.93(0.80,3.60) |  | 100.48(-7.95,424.42) | 1.87(1.45,2.29) |
| Gambia | 2.64(1.02,4.38) | 1.42(0.55,2.36) |  | 4.09(1.32,9.78) | 1.14(0.37,2.74) |  | 54.85(-49.49,269.76) | -0.96(-1.30,-0.61) |
| Georgia | 78.67(50.24,113.67) | 16.79(10.72,24.26) |  | 14.17(8.40,22.03) | 5.82(3.45,9.06) |  | -81.99(-90.25,-66.68) | -4.20(-4.96,-3.44) |
| Germany | 2443.07(1578.92,3548.44) | 54.54(35.25,79.22) |  | 3045.97(2219.85,4005.98) | 75.33(54.90,99.07) |  | 24.68(-22.75,113.60) | 0.11(-0.51,0.72) |
| Ghana | 111.44(33.25,211.07) | 4.23(1.26,8.01) |  | 124.45(43.08,276.62) | 2.68(0.93,5.96) |  | 11.67(-66.54,231.56) | -2.36(-3.13,-1.57) |
| Greece | 106172.66(88471.54,133096.57) | 17.13(14.27,21.47) |  | 168879.41(103720.68,240540.11) | 25.66(15.76,36.55) |  | 59.06(-6.12,146.38) | 2.26(1.93,2.60) |
| Greenland | 624.15(406.47,906.89) | 112.46(73.24,163.41) |  | 371.99(257.24,490.77) | 88.02(60.87,116.13) |  | -40.40(-63.95,-2.36) | -0.78(-1.14,-0.42) |
| Grenada | 0.24(0.12,0.41) | 4.40(2.09,7.46) |  | 0.29(0.15,0.54) | 7.23(3.61,13.48) |  | 20.70(-49.59,188.11) | 2.39(2.17,2.60) |
| Guam | 0.49(0.33,0.72) | 4.05(2.74,5.97) |  | 0.40(0.23,0.62) | 5.77(3.42,9.05) |  | -18.60(-54.77,42.29) | 1.44(0.98,1.91) |
| Guatemala | 1.28(0.79,1.95) | 7.94(4.90,12.07) |  | 1.69(0.93,2.83) | 13.24(7.25,22.14) |  | 31.83(-33.19,150.08) | 3.23(2.17,4.30) |
| Guinea | 76.00(62.37,92.35) | 4.95(4.07,6.02) |  | 128.04(82.64,190.11) | 8.21(5.30,12.19) |  | 68.47(2.21,161.48) | 2.11(1.95,2.26) |
| Guinea-Bissau | 16.64(6.08,28.40) | 1.43(0.52,2.45) |  | 19.81(4.69,50.52) | 0.88(0.21,2.25) |  | 19.04(-54.84,218.98) | -1.14(-1.30,-0.98) |
| Guyana | 4.36(1.75,9.05) | 2.32(0.93,4.81) |  | 4.28(1.19,8.06) | 1.29(0.36,2.42) |  | -1.87(-65.71,181.22) | -1.30(-1.72,-0.87) |
| Haiti | 3.70(2.68,5.00) | 3.28(2.37,4.43) |  | 3.14(2.01,4.67) | 4.21(2.70,6.27) |  | -15.16(-49.17,38.17) | 1.67(1.12,2.22) |
| Honduras | 99.33(15.76,236.00) | 9.36(1.49,22.25) |  | 107.09(27.85,245.33) | 6.82(1.77,15.63) |  | 7.82(-39.75,158.01) | -0.68(-0.89,-0.47) |
| Hungary | 50.65(33.52,73.53) | 6.15(4.07,8.93) |  | 43.79(16.20,105.05) | 4.00(1.48,9.59) |  | -13.54(-70.64,97.42) | -1.41(-1.48,-1.34) |
| Iceland | 90.53(62.84,126.96) | 14.62(10.15,20.51) |  | 110.10(62.45,186.65) | 24.23(13.74,41.08) |  | 21.61(-36.17,128.53) | 1.47(0.89,2.04) |
| India | 14.00(8.77,19.95) | 66.29(41.52,94.43) |  | 14.28(9.99,20.12) | 65.00(45.47,91.58) |  | 1.99(-36.26,79.99) | -0.33(-0.78,0.12) |
| Indonesia | 2555.33(1326.39,4657.33) | 2.20(1.14,4.01) |  | 2826.28(1522.25,4450.68) | 2.54(1.37,4.00) |  | 10.60(-53.55,183.61) | 0.28(-0.11,0.68) |
| Iran (Islamic Republic of) | 1229.13(442.05,2639.53) | 5.51(1.98,11.83) |  | 1655.76(1007.52,2473.29) | 7.56(4.60,11.29) |  | 34.71(-23.86,234.13) | 0.93(0.67,1.18) |
| Iraq | 1541.45(822.37,2805.83) | 17.57(9.37,31.99) |  | 1239.77(471.28,2627.02) | 20.14(7.66,42.68) |  | -19.57(-73.90,153.58) | 2.20(1.46,2.95) |
| Ireland | 208.48(109.56,365.92) | 6.63(3.49,11.64) |  | 1083.85(479.96,1991.42) | 25.24(11.18,46.38) |  | 419.88(112.07,1287.66) | 5.28(4.78,5.79) |
| Israel | 181.43(114.39,267.11) | 62.78(39.59,92.43) |  | 132.17(99.85,182.49) | 44.29(33.46,61.16) |  | -27.15(-53.82,26.26) | -0.16(-0.83,0.52) |
| Italy | 342.78(221.64,507.82) | 66.38(42.92,98.35) |  | 424.48(289.08,619.73) | 46.23(31.48,67.49) |  | 23.83(-29.14,112.96) | 0.10(-0.43,0.64) |
| Jamaica | 4130.87(2869.61,5637.32) | 150.45(104.51,205.31) |  | 2068.17(1618.04,2558.66) | 95.30(74.56,117.90) |  | -49.93(-66.43,-21.80) | -1.77(-2.17,-1.37) |
| Japan | 40.72(27.02,58.70) | 14.59(9.68,21.04) |  | 17.98(10.57,27.95) | 10.50(6.17,16.33) |  | -55.84(-75.66,-19.77) | -0.45(-0.67,-0.23) |
| Jordan | 5410.12(3771.98,7591.93) | 81.25(56.65,114.02) |  | 3002.09(2460.22,3472.85) | 65.47(53.65,75.74) |  | -44.51(-62.46,-16.75) | -0.82(-1.22,-0.41) |
| Kazakhstan | 34.99(18.39,58.54) | 5.83(3.06,9.75) |  | 208.22(86.47,401.68) | 18.98(7.88,36.61) |  | 495.10(110.52,1283.72) | 4.80(4.30,5.29) |
| Kenya | 139.91(88.74,204.13) | 7.43(4.71,10.84) |  | 185.73(122.06,274.47) | 9.53(6.26,14.09) |  | 32.75(-26.33,126.87) | 3.10(1.99,4.22) |
| Kiribati | 87.25(44.49,147.09) | 2.03(1.04,3.43) |  | 86.64(36.33,217.72) | 1.46(0.61,3.66) |  | -0.70(-58.06,139.51) | -0.61(-1.14,-0.07) |
| Kuwait | 0.20(0.11,0.33) | 1.73(0.92,2.82) |  | 0.17(0.08,0.35) | 1.20(0.58,2.46) |  | -16.11(-62.64,120.06) | -1.23(-1.49,-0.97) |
| Kyrgyzstan | 69.26(43.79,102.05) | 33.88(21.42,49.91) |  | 118.18(65.88,197.27) | 44.60(24.86,74.44) |  | 70.64(-11.24,238.43) | 1.86(1.45,2.27) |
| Lao People's Democratic Republic | 42.24(30.41,59.28) | 6.57(4.73,9.22) |  | 59.89(36.08,90.93) | 7.54(4.54,11.45) |  | 41.77(-21.56,148.36) | 0.68(-0.01,1.37) |
| Latvia | 42.31(6.92,103.10) | 5.95(0.97,14.50) |  | 41.04(19.78,74.80) | 4.95(2.38,9.02) |  | -2.99(-54.69,450.02) | -0.65(-0.81,-0.49) |
| Lebanon | 42.87(28.63,64.57) | 21.16(14.13,31.87) |  | 14.36(8.58,23.46) | 15.33(9.15,25.03) |  | -66.50(-82.99,-38.09) | -1.02(-1.56,-0.47) |
| Lesotho | 27.94(13.53,51.64) | 7.17(3.47,13.26) |  | 105.52(33.04,229.17) | 26.00(8.14,56.46) |  | 277.64(-5.52,1189.12) | 4.23(3.51,4.94) |
| Liberia | 2.14(1.06,3.99) | 0.87(0.43,1.62) |  | 1.99(1.05,3.46) | 0.98(0.52,1.70) |  | -6.95(-50.35,88.47) | 0.95(0.33,1.57) |
| Libya | 11.42(4.19,24.09) | 2.46(0.90,5.19) |  | 17.01(4.44,33.24) | 2.22(0.58,4.34) |  | 48.97(-57.20,501.91) | -0.01(-0.78,0.77) |
| Lithuania | 74.62(36.86,133.90) | 11.72(5.79,21.02) |  | 156.68(62.45,299.26) | 37.03(14.76,70.74) |  | 109.97(-14.34,354.38) | 4.87(4.22,5.51) |
| Luxembourg | 81.88(55.00,119.71) | 28.32(19.03,41.41) |  | 22.37(13.26,35.57) | 17.00(10.08,27.03) |  | -72.68(-85.01,-50.83) | -1.80(-2.50,-1.10) |
| Madagascar | 14.44(9.68,21.20) | 63.07(42.26,92.60) |  | 18.59(13.35,25.92) | 56.21(40.37,78.40) |  | 28.70(-21.26,107.15) | -0.68(-1.20,-0.16) |
| Malawi | 72.86(41.49,113.77) | 3.38(1.92,5.28) |  | 93.85(38.75,182.11) | 2.30(0.95,4.45) |  | 28.82(-49.38,199.65) | -0.89(-1.12,-0.67) |
| Malaysia | 42.56(22.15,65.26) | 2.24(1.17,3.44) |  | 32.07(8.25,80.69) | 1.18(0.30,2.96) |  | -24.66(-77.44,99.97) | -2.11(-2.42,-1.80) |
| Maldives | 99.99(44.99,188.16) | 4.20(1.89,7.90) |  | 232.17(115.16,397.02) | 9.44(4.68,16.15) |  | 132.20(-6.38,532.86) | 3.84(3.29,4.40) |
| Mali | 2.30(0.59,5.15) | 5.49(1.41,12.32) |  | 8.77(4.24,16.68) | 27.61(13.36,52.54) |  | 281.91(17.96,2084.98) | 6.63(5.88,7.40) |
| Malta | 43.56(15.69,73.51) | 2.52(0.91,4.25) |  | 68.97(16.83,169.06) | 1.51(0.37,3.69) |  | 58.33(-33.86,290.72) | -1.34(-1.50,-1.18) |
| Marshall Islands | 13.68(8.50,20.28) | 48.44(30.08,71.79) |  | 14.75(9.68,21.20) | 67.03(43.98,96.37) |  | 7.81(-39.30,83.72) | 1.70(1.31,2.10) |
| Mauritania | 0.06(0.03,0.09) | 0.75(0.43,1.18) |  | 0.05(0.03,0.10) | 0.96(0.48,1.71) |  | -2.16(-51.22,90.36) | 0.62(0.20,1.04) |
| Mauritius | 5.74(2.58,9.70) | 1.55(0.70,2.61) |  | 20.68(5.91,46.00) | 3.15(0.90,7.00) |  | 260.55(20.60,672.68) | 2.05(1.83,2.28) |
| Mexico | 7.93(5.56,10.75) | 7.52(5.27,10.19) |  | 7.06(4.91,9.97) | 10.98(7.64,15.50) |  | -10.93(-44.44,43.81) | 2.04(0.25,3.87) |
| Micronesia (Federated States of) | 1410.25(1229.01,1641.19) | 11.95(10.42,13.91) |  | 2126.17(1508.59,2982.57) | 21.53(15.27,30.20) |  | 50.77(3.50,116.92) | 1.93(1.10,2.76) |
| Monaco | 0.20(0.11,0.32) | 1.25(0.67,2.06) |  | 0.09(0.04,0.18) | 0.96(0.47,1.85) |  | -53.48(-80.17,19.88) | -0.67(-0.85,-0.48) |
| Mongolia | 5.38(2.20,10.13) | 459.26(187.47,865.45) |  | 6.46(2.85,11.27) | 399.44(176.41,696.69) |  | 20.19(-48.18,195.36) | -2.35(-3.14,-1.56) |
| Montenegro | 15.63(8.17,28.18) | 4.60(2.41,8.30) |  | 14.56(6.82,26.49) | 3.73(1.75,6.78) |  | -6.83(-65.23,135.50) | -0.16(-0.65,0.34) |
| Morocco | 20.70(11.22,35.88) | 39.45(21.38,68.38) |  | 6.07(2.48,11.61) | 16.77(6.86,32.08) |  | -70.67(-88.08,-25.96) | -0.37(-1.03,0.29) |
| Mozambique | 48.02(22.16,94.68) | 1.35(0.62,2.66) |  | 69.85(26.06,152.08) | 2.15(0.80,4.68) |  | 45.46(-50.64,414.32) | 1.85(1.61,2.09) |
| Myanmar | 263.60(138.87,437.61) | 10.94(5.76,18.17) |  | 289.38(82.70,707.68) | 5.59(1.60,13.66) |  | 9.78(-69.82,225.87) | -2.01(-2.19,-1.83) |
| Namibia | 429.78(74.40,1024.74) | 8.52(1.48,20.32) |  | 510.84(242.53,928.90) | 9.77(4.64,17.77) |  | 18.86(-48.45,500.41) | 0.18(-0.10,0.47) |
| Nauru | 2.73(1.25,5.34) | 1.21(0.55,2.36) |  | 7.26(3.30,13.30) | 2.61(1.19,4.77) |  | 165.85(6.09,537.45) | 3.41(2.40,4.44) |
| Nepal | 0.03(0.02,0.05) | 1.78(1.02,2.96) |  | 0.03(0.02,0.06) | 2.38(1.25,4.07) |  | 14.30(-41.37,123.87) | 0.55(0.33,0.78) |
| Netherlands | 96.38(27.75,222.21) | 2.93(0.84,6.75) |  | 70.11(34.57,124.96) | 2.26(1.11,4.02) |  | -27.25(-76.88,199.54) | -0.75(-0.99,-0.51) |
| New Zealand | 845.12(559.36,1237.08) | 90.26(59.74,132.12) |  | 345.28(254.66,458.74) | 40.09(29.57,53.27) |  | -59.14(-75.42,-29.22) | -2.44(-2.99,-1.89) |
| Nicaragua | 108.05(75.43,152.38) | 38.64(26.97,54.49) |  | 160.26(110.64,214.07) | 51.27(35.39,68.48) |  | 48.32(-8.20,132.52) | 1.85(1.24,2.45) |
| Niger | 97.98(60.89,151.96) | 14.77(9.18,22.90) |  | 94.31(45.38,180.84) | 14.50(6.98,27.80) |  | -3.75(-61.08,115.68) | 0.68(0.43,0.93) |
| Nigeria | 49.82(20.05,104.25) | 2.95(1.19,6.18) |  | 97.81(26.09,187.78) | 1.92(0.51,3.68) |  | 96.35(-29.87,547.78) | -1.06(-1.25,-0.86) |
| Niue | 444.76(156.42,857.12) | 2.80(0.98,5.39) |  | 1273.83(297.29,2278.19) | 3.43(0.80,6.14) |  | 186.41(26.54,605.64) | 0.98(0.77,1.19) |
| North Macedonia | 0.01(0.00,0.01) | 1.98(1.09,3.59) |  | 0.02(0.01,0.04) | 20.37(9.93,35.54) |  | 373.99(131.80,854.15) | 4.33(3.13,5.54) |
| Northern Mariana Islands | 21.19(11.60,35.17) | 12.44(6.81,20.65) |  | 11.17(4.18,25.45) | 11.12(4.16,25.32) |  | -47.27(-83.47,64.68) | 2.28(1.44,3.14) |
| Norway | 0.14(0.07,0.24) | 2.93(1.43,5.04) |  | 0.09(0.04,0.17) | 2.85(1.39,5.40) |  | -34.33(-70.21,35.90) | -0.69(-1.35,-0.02) |
| Oman | 142.42(96.76,191.13) | 51.63(35.08,69.29) |  | 126.76(99.44,160.22) | 45.09(35.37,56.99) |  | -11.00(-37.57,41.71) | -0.20(-0.65,0.26) |
| Pakistan | 6.91(2.28,14.68) | 2.11(0.70,4.48) |  | 22.80(7.72,48.08) | 5.38(1.82,11.34) |  | 229.78(-11.59,1163.36) | 3.71(2.74,4.68) |
| Palau | 460.55(192.60,904.66) | 2.49(1.04,4.90) |  | 893.26(446.63,1558.04) | 3.00(1.50,5.24) |  | 93.96(10.76,283.46) | 1.25(0.75,1.75) |
| Palestine | 0.01(0.00,0.06) | 0.81(0.06,4.22) |  | 0.01(0.00,0.05) | 1.05(0.08,5.29) |  | -17.38(-76.18,175.21) | 0.76(0.47,1.04) |
| Panama | 42.87(18.10,80.47) | 10.99(4.64,20.63) |  | 146.13(77.06,241.47) | 23.85(12.58,39.41) |  | 240.88(31.34,791.02) | 2.95(2.75,3.14) |
| Papua New Guinea | 39.87(26.88,55.39) | 13.96(9.41,19.40) |  | 149.62(95.69,224.17) | 40.30(25.78,60.38) |  | 275.23(118.42,571.10) | 3.08(2.67,3.49) |
| Paraguay | 13.88(4.64,28.89) | 2.14(0.72,4.46) |  | 32.71(13.20,66.23) | 2.15(0.87,4.35) |  | 135.65(35.20,376.49) | -0.11(-0.40,0.18) |
| Peru | 61.29(37.36,96.23) | 9.93(6.06,15.60) |  | 130.59(70.89,249.39) | 20.09(10.90,38.36) |  | 113.07(3.77,341.96) | 2.97(2.67,3.27) |
| Philippines | 271.01(168.52,464.04) | 9.26(5.76,15.86) |  | 1128.13(435.27,2063.69) | 34.18(13.19,62.53) |  | 316.28(18.72,883.66) | 5.73(5.26,6.21) |
| Poland | 720.49(367.71,1288.65) | 7.79(3.97,13.93) |  | 917.62(534.59,1350.43) | 8.18(4.77,12.04) |  | 27.36(-43.02,212.38) | 0.59(0.26,0.92) |
| Portugal | 275.43(173.96,430.22) | 9.44(5.96,14.74) |  | 419.49(271.51,618.19) | 22.29(14.43,32.85) |  | 52.31(-20.33,191.87) | 2.24(1.51,2.97) |
| Puerto Rico | 269.03(178.65,399.30) | 46.51(30.89,69.03) |  | 304.17(199.14,440.22) | 71.52(46.82,103.50) |  | 13.06(-38.03,92.35) | 1.59(1.12,2.07) |
| Qatar | 52.87(35.37,76.65) | 16.57(11.08,24.01) |  | 28.27(16.68,46.60) | 26.89(15.87,44.33) |  | -46.53(-70.92,-3.25) | 2.89(2.50,3.29) |
| Republic of Korea | 2.33(1.22,4.19) | 4.58(2.40,8.25) |  | 27.59(11.79,63.95) | 14.97(6.40,34.70) |  | 1085.00(311.82,2706.38) | 4.70(4.39,5.02) |
| Republic of Moldova | 375.88(208.91,588.34) | 11.32(6.29,17.73) |  | 812.02(254.63,1555.02) | 52.39(16.43,100.33) |  | 116.03(-27.40,436.35) | 6.20(4.99,7.42) |
| Romania | 106.23(71.77,148.21) | 24.66(16.66,34.40) |  | 35.10(21.62,55.89) | 22.76(14.02,36.24) |  | -66.95(-82.05,-41.13) | -0.06(-1.03,0.92) |
| Russian Federation | 211.54(149.38,298.21) | 11.95(8.44,16.85) |  | 172.80(114.39,266.58) | 18.42(12.20,28.42) |  | -18.32(-53.29,49.69) | 1.92(1.24,2.61) |
| Rwanda | 2384.32(2169.63,2583.74) | 20.51(18.66,22.23) |  | 2167.44(1816.49,2599.97) | 28.48(23.87,34.16) |  | -9.10(-23.84,8.88) | 0.97(0.02,1.93) |
| Saint Kitts and Nevis | 50.00(28.10,85.43) | 3.71(2.09,6.34) |  | 56.63(20.69,118.84) | 3.24(1.18,6.80) |  | 13.26(-64.69,225.34) | -0.56(-0.92,-0.19) |
| Saint Lucia | 0.21(0.16,0.29) | 4.58(3.37,6.16) |  | 0.33(0.19,0.55) | 10.70(6.28,17.96) |  | 52.18(-13.96,161.44) | 3.87(3.08,4.68) |
| Saint Vincent and the Grenadines | 0.86(0.59,1.23) | 4.87(3.37,6.98) |  | 0.55(0.31,0.97) | 6.19(3.56,10.96) |  | -36.49(-65.82,20.79) | 1.49(0.89,2.10) |
| Samoa | 0.61(0.40,0.87) | 4.76(3.15,6.80) |  | 0.30(0.18,0.49) | 4.14(2.53,6.81) |  | -51.02(-73.41,-10.53) | -0.23(-0.63,0.18) |
| San Marino | 0.56(0.31,1.00) | 2.20(1.21,3.91) |  | 0.79(0.32,1.76) | 2.70(1.09,6.03) |  | 41.17(-39.79,258.00) | 0.49(0.25,0.73) |
| Sao Tome and Principe | 6.87(3.42,11.83) | 579.32(287.87,997.09) |  | 2.71(1.41,4.50) | 224.30(116.30,371.95) |  | -60.54(-82.78,0.30) | -2.50(-2.77,-2.24) |
| Saudi Arabia | 0.37(0.18,0.63) | 1.83(0.88,3.08) |  | 0.26(0.05,0.79) | 1.05(0.19,3.19) |  | -30.14(-88.12,139.16) | -1.79(-2.60,-0.98) |
| Senegal | 108.33(56.89,191.32) | 4.48(2.35,7.91) |  | 242.71(103.34,539.16) | 9.98(4.25,22.16) |  | 124.05(-24.90,545.94) | 3.20(2.89,3.51) |
| Serbia | 38.61(16.28,68.96) | 2.64(1.11,4.71) |  | 45.48(12.27,109.18) | 2.00(0.54,4.81) |  | 17.78(-64.78,286.65) | -0.65(-0.97,-0.32) |
| Seychelles | 81.29(37.85,155.11) | 11.94(5.56,22.79) |  | 43.75(17.67,89.10) | 11.87(4.79,24.17) |  | -46.18(-81.95,59.56) | -0.14(-0.64,0.37) |
| Sierra Leone | 0.20(0.10,0.36) | 2.44(1.25,4.47) |  | 0.51(0.25,0.90) | 6.46(3.16,11.46) |  | 158.84(15.85,480.21) | 3.68(3.30,4.07) |
| Singapore | 22.66(8.50,48.01) | 2.93(1.10,6.20) |  | 35.78(9.61,71.00) | 2.66(0.72,5.29) |  | 57.87(-38.10,390.68) | -0.20(-0.41,0.02) |
| Slovakia | 66.65(45.86,98.58) | 32.66(22.47,48.31) |  | 170.31(114.47,230.12) | 59.56(40.03,80.48) |  | 155.53(42.81,320.81) | 2.84(2.43,3.26) |
| Slovenia | 40.71(21.88,66.02) | 9.95(5.35,16.13) |  | 62.74(28.56,120.18) | 21.93(9.98,42.00) |  | 54.12(-37.65,271.55) | 2.93(2.68,3.17) |
| Solomon Islands | 28.93(19.50,41.54) | 23.52(15.86,33.78) |  | 35.35(22.02,53.00) | 36.10(22.50,54.14) |  | 22.18(-32.37,115.78) | 1.79(1.21,2.38) |
| Somalia | 0.68(0.37,1.12) | 1.14(0.63,1.88) |  | 0.92(0.46,1.78) | 0.96(0.48,1.87) |  | 34.87(-31.94,223.74) | -0.61(-0.93,-0.29) |
| South Africa | 49.30(20.91,98.98) | 3.19(1.35,6.41) |  | 71.84(33.02,131.89) | 1.74(0.80,3.19) |  | 45.73(-29.87,242.46) | -1.23(-1.71,-0.75) |
| South Sudan | 112.20(51.24,212.50) | 2.30(1.05,4.36) |  | 146.28(96.52,211.90) | 2.95(1.95,4.27) |  | 30.38(-28.56,205.79) | 2.08(0.67,3.50) |
| Spain | 46.68(21.95,88.59) | 4.59(2.16,8.70) |  | 75.32(36.87,137.36) | 4.82(2.36,8.79) |  | 61.36(-2.83,190.53) | 0.86(0.19,1.53) |
| Sri Lanka | 2055.46(1347.40,3170.55) | 98.69(64.69,152.23) |  | 1564.63(1198.82,2026.09) | 84.98(65.11,110.04) |  | -23.88(-53.95,20.84) | -0.95(-1.50,-0.39) |
| Sudan | 114.59(69.76,180.24) | 6.48(3.95,10.20) |  | 196.45(86.55,382.33) | 12.55(5.53,24.42) |  | 71.43(-29.99,307.02) | 2.91(2.43,3.39) |
| Suriname | 294.74(62.54,723.69) | 8.47(1.80,20.79) |  | 701.05(289.38,1354.19) | 12.43(5.13,24.01) |  | 137.85(3.52,1072.67) | 1.41(1.26,1.56) |
| Sweden | 1.55(0.77,2.34) | 3.54(1.76,5.33) |  | 2.09(1.18,3.45) | 4.69(2.64,7.74) |  | 34.55(-27.18,176.00) | 1.02(0.82,1.23) |
| Switzerland | 800.98(547.54,1124.33) | 142.26(97.24,199.68) |  | 347.76(275.77,441.46) | 59.63(47.29,75.70) |  | -56.58(-71.00,-31.42) | -3.13(-3.80,-2.46) |
| Syrian Arab Republic | 486.77(326.38,675.68) | 122.20(81.94,169.63) |  | 294.68(211.60,406.68) | 66.67(47.87,92.01) |  | -39.46(-62.91,3.40) | -2.70(-3.20,-2.21) |
| Taiwan (Province of China) | 186.82(83.62,343.46) | 8.66(3.88,15.92) |  | 230.73(100.79,435.71) | 22.96(10.03,43.35) |  | 23.50(-37.01,151.39) | 3.92(3.53,4.31) |
| Tajikistan | 166.61(95.85,264.62) | 10.37(5.97,16.47) |  | 353.04(212.72,539.01) | 39.57(23.84,60.41) |  | 111.89(5.18,347.32) | 5.29(4.74,5.84) |
| Thailand | 79.70(37.01,128.46) | 8.43(3.91,13.59) |  | 87.06(38.05,180.92) | 6.50(2.84,13.52) |  | 9.23(-54.88,132.86) | -1.06(-1.65,-0.47) |
| Timor-Leste | 378.02(199.49,671.91) | 7.27(3.84,12.92) |  | 770.43(400.09,1273.24) | 27.25(14.15,45.04) |  | 103.81(-13.81,345.30) | 4.83(4.53,5.13) |
| Togo | 9.93(1.81,23.90) | 7.09(1.29,17.07) |  | 11.48(5.29,22.04) | 6.21(2.86,11.93) |  | 15.62(-44.26,426.94) | -0.45(-0.85,-0.06) |
| Tokelau | 14.01(5.95,24.25) | 2.04(0.87,3.53) |  | 24.58(5.75,54.20) | 2.10(0.49,4.62) |  | 75.49(-33.70,347.07) | 0.18(-0.07,0.43) |
| Tonga | 0.00(0.00,0.01) | 1.61(0.78,2.72) |  | 0.02(0.01,0.06) | 23.22(5.85,62.49) |  | 634.16(86.38,1940.00) | 3.77(1.67,5.92) |
| Trinidad and Tobago | 0.19(0.10,0.35) | 1.27(0.65,2.30) |  | 0.28(0.14,0.55) | 1.97(0.94,3.79) |  | 46.21(-35.10,260.60) | 1.03(0.81,1.26) |
| Tunisia | 6.12(4.45,8.34) | 4.59(3.34,6.25) |  | 6.61(3.92,10.44) | 8.21(4.87,12.96) |  | 8.11(-41.77,97.77) | 2.39(1.97,2.82) |
| Turkey | 122.95(61.09,219.19) | 11.52(5.72,20.54) |  | 155.27(52.01,318.84) | 17.41(5.83,35.74) |  | 26.28(-60.18,269.13) | 1.55(1.35,1.76) |
| Turkmenistan | 873.17(412.68,1650.65) | 12.55(5.93,23.73) |  | 2068.46(1113.10,3555.47) | 37.26(20.05,64.04) |  | 136.89(-12.56,560.32) | 4.42(3.61,5.25) |
| Tuvalu | 28.47(18.11,46.45) | 4.86(3.09,7.93) |  | 28.17(17.70,44.06) | 5.22(3.28,8.16) |  | -1.04(-43.95,68.31) | 0.55(0.17,0.92) |
| Uganda | 0.02(0.01,0.05) | 1.63(0.48,3.35) |  | 0.02(0.01,0.03) | 1.26(0.69,2.15) |  | -34.31(-72.52,174.05) | -0.78(-0.92,-0.64) |
| Ukraine | 102.71(54.53,169.63) | 2.86(1.52,4.72) |  | 200.94(68.31,425.18) | 2.75(0.93,5.81) |  | 95.64(-22.23,340.95) | 0.22(-0.13,0.57) |
| United Arab Emirates | 1550.08(1060.21,2326.18) | 41.13(28.13,61.72) |  | 390.10(243.94,620.47) | 24.50(15.32,38.97) |  | -74.83(-86.47,-54.78) | -0.77(-1.34,-0.21) |
| United Kingdom | 8.14(4.32,14.98) | 3.57(1.89,6.57) |  | 24.77(9.95,47.70) | 5.72(2.30,11.03) |  | 204.37(2.09,782.74) | 2.89(2.19,3.59) |
| United Republic of Tanzania | 3765.57(3292.89,4305.05) | 98.02(85.72,112.07) |  | 1997.39(1733.56,2283.97) | 54.65(47.43,62.50) |  | -46.96(-56.84,-35.80) | -1.15(-1.68,-0.61) |
| United States of America | 226.25(136.47,347.02) | 4.71(2.84,7.22) |  | 392.26(153.96,761.30) | 4.43(1.74,8.61) |  | 73.37(-35.08,305.74) | 0.29(0.02,0.57) |
| United States Virgin Islands | 14770.71(13613.51,16110.52) | 74.79(68.93,81.57) |  | 10293.38(8971.10,11909.11) | 55.36(48.25,64.05) |  | -30.31(-40.31,-18.30) | -0.48(-0.77,-0.19) |
| Uruguay | 0.63(0.33,1.03) | 5.70(3.03,9.32) |  | 0.37(0.13,0.82) | 9.51(3.31,21.00) |  | -40.57(-81.29,57.74) | 1.66(1.41,1.91) |
| Uzbekistan | 33.60(23.30,46.01) | 12.32(8.54,16.86) |  | 30.29(18.41,49.27) | 15.62(9.49,25.40) |  | -9.85(-50.09,62.59) | 0.66(0.14,1.19) |
| Vanuatu | 235.83(146.60,378.46) | 6.99(4.35,11.22) |  | 395.66(234.94,650.46) | 10.32(6.13,16.96) |  | 67.77(-15.65,213.71) | 1.84(1.21,2.48) |
| Venezuela (Bolivarian Republic of) | 0.24(0.13,0.39) | 0.87(0.47,1.44) |  | 0.30(0.16,0.52) | 0.72(0.39,1.23) |  | 28.95(-34.29,150.08) | -0.54(-1.08,-0.01) |
| Viet Nam | 181.85(138.21,236.34) | 7.19(5.46,9.34) |  | 461.07(235.26,833.17) | 21.10(10.77,38.13) |  | 153.54(25.52,350.38) | 4.27(3.73,4.81) |
| Yemen | 487.06(252.12,912.31) | 5.18(2.68,9.70) |  | 1453.78(737.02,2612.56) | 17.86(9.05,32.09) |  | 198.48(30.14,569.37) | 4.37(4.15,4.59) |
| Zambia | 158.47(43.47,391.30) | 5.71(1.57,14.09) |  | 273.26(111.36,498.13) | 5.82(2.37,10.60) |  | 72.44(-19.59,533.03) | 0.62(0.32,0.91) |
| Zimbabwe | 70.94(39.42,119.50) | 4.70(2.61,7.92) |  | 95.63(39.93,197.86) | 3.27(1.37,6.76) |  | 34.81(-53.41,299.24) | -0.88(-1.13,-0.62) |
