# Supplementary material for: Analysis of global trends in acute lymphoblastic leukemia in children aged 0–5 years from 1990 to 2021
Source: Front Pediatr. 2025 Mar 13;13:1542649. doi: 10.3389/fped.2025.1542649 (PMC11966407; doi:10.3389/fped.2025.1542649)
Supplement: Supplementary file 5 [file Table4.docx]

**Table S4.** National Mortality of Acute Lymphoblastic Leukemia in Children Aged 0–5 Years from 1990 to 2021.

| location | 1990 | |  | 2021 | |  | 1990-2021 | |
| --- | --- | --- | --- | --- | --- | --- | --- | --- |
|  | Death case | Death rate |  | Death case | Death rate |  | Cases change | EAPC |
| Afghanistan | 54.45(7.75,127.17) | 3.18(0.45,7.42) |  | 108.23(49.79,221.57) | 1.97(0.91,4.04) |  | 98.79(5.65,866.98) | -1.21(-1.48,-0.95) |
| Albania | 7.79(4.04,12.33) | 1.93(1.00,3.05) |  | 1.58(0.85,2.68) | 1.11(0.60,1.88) |  | -79.69(-89.48,-58.11) | -2.07(-2.54,-1.60) |
| Algeria | 25.44(12.88,42.53) | 0.68(0.34,1.14) |  | 10.98(5.52,18.51) | 0.23(0.12,0.39) |  | -56.82(-80.65,2.44) | -3.09(-3.21,-2.96) |
| American Samoa | 0.04(0.02,0.05) | 0.48(0.30,0.71) |  | 0.01(0.01,0.02) | 0.31(0.16,0.57) |  | -68.51(-84.54,-26.24) | -1.43(-1.66,-1.21) |
| Andorra | 0.03(0.01,0.06) | 1.23(0.48,2.37) |  | 0.01(0.00,0.01) | 0.28(0.15,0.44) |  | -79.02(-90.79,-35.57) | -3.91(-4.20,-3.62) |
| Angola | 33.37(5.32,84.51) | 1.71(0.27,4.33) |  | 41.27(18.59,88.96) | 0.73(0.33,1.58) |  | 23.67(-40.45,455.95) | -2.48(-2.75,-2.22) |
| Antigua and Barbuda | 0.05(0.04,0.06) | 0.82(0.63,1.04) |  | 0.03(0.03,0.04) | 0.65(0.49,0.81) |  | -31.08(-49.08,-9.46) | -0.07(-0.52,0.38) |
| Argentina | 48.59(41.47,57.94) | 1.41(1.21,1.69) |  | 18.15(13.28,24.29) | 0.61(0.44,0.81) |  | -62.65(-73.42,-46.99) | -2.10(-2.34,-1.87) |
| Armenia | 11.33(9.58,13.55) | 2.96(2.50,3.54) |  | 1.20(0.86,1.65) | 0.65(0.46,0.89) |  | -89.36(-92.66,-84.56) | -4.51(-5.01,-4.02) |
| Australia | 7.19(6.08,8.49) | 0.57(0.48,0.67) |  | 2.49(1.88,3.20) | 0.17(0.13,0.21) |  | -65.33(-75.19,-52.35) | -3.13(-3.37,-2.89) |
| Austria | 2.61(2.21,3.03) | 0.58(0.49,0.68) |  | 0.68(0.52,0.90) | 0.16(0.12,0.21) |  | -73.80(-81.31,-62.92) | -3.92(-4.24,-3.61) |
| Azerbaijan | 27.25(16.79,39.06) | 3.02(1.86,4.32) |  | 8.98(4.64,16.08) | 1.25(0.64,2.23) |  | -67.03(-83.84,-37.43) | -2.58(-2.74,-2.43) |
| Bahamas | 0.20(0.15,0.24) | 0.77(0.60,0.95) |  | 0.06(0.04,0.10) | 0.30(0.19,0.47) |  | -67.36(-79.33,-47.54) | -2.35(-2.56,-2.15) |
| Bahrain | 0.36(0.20,0.62) | 0.59(0.33,1.00) |  | 0.16(0.09,0.30) | 0.17(0.09,0.31) |  | -55.92(-75.33,-20.57) | -3.40(-3.73,-3.06) |
| Bangladesh | 393.56(109.91,892.95) | 2.08(0.58,4.72) |  | 78.58(46.15,129.42) | 0.55(0.32,0.90) |  | -80.03(-93.03,-23.41) | -4.21(-4.33,-4.09) |
| Barbados | 0.13(0.10,0.16) | 0.67(0.51,0.84) |  | 0.04(0.02,0.05) | 0.26(0.17,0.39) |  | -72.62(-81.57,-59.97) | -3.04(-3.50,-2.57) |
| Belarus | 13.60(10.74,16.78) | 1.69(1.33,2.08) |  | 1.37(0.86,2.14) | 0.29(0.18,0.46) |  | -89.92(-93.75,-82.94) | -5.40(-6.04,-4.76) |
| Belgium | 4.72(3.97,5.73) | 0.79(0.67,0.96) |  | 1.19(0.84,1.65) | 0.20(0.14,0.28) |  | -74.86(-82.52,-63.03) | -4.21(-4.66,-3.75) |
| Belize | 0.82(0.69,0.96) | 2.78(2.34,3.25) |  | 0.26(0.20,0.34) | 0.69(0.53,0.89) |  | -67.99(-76.40,-56.95) | -3.92(-4.21,-3.63) |
| Benin | 15.22(6.66,26.66) | 1.54(0.67,2.70) |  | 29.59(8.27,58.69) | 1.26(0.35,2.51) |  | 94.39(-25.74,374.88) | -0.21(-0.42,-0.01) |
| Bermuda | 0.02(0.01,0.04) | 0.52(0.28,0.86) |  | 0.01(0.01,0.01) | 0.30(0.20,0.44) |  | -66.23(-80.20,-30.02) | -1.45(-1.87,-1.02) |
| Bhutan | 1.57(0.52,3.25) | 1.64(0.55,3.40) |  | 0.50(0.26,0.86) | 0.81(0.43,1.40) |  | -68.43(-88.91,11.78) | -2.76(-3.15,-2.36) |
| Bolivia (Plurinational State of) | 63.98(28.56,118.96) | 6.33(2.83,11.78) |  | 32.49(18.22,51.85) | 2.72(1.53,4.34) |  | -49.22(-79.51,55.21) | -2.65(-2.76,-2.54) |
| Bosnia and Herzegovina | 1.95(1.18,2.89) | 0.55(0.34,0.82) |  | 0.24(0.13,0.38) | 0.16(0.09,0.25) |  | -87.84(-93.52,-77.07) | -3.63(-4.13,-3.13) |
| Botswana | 1.13(0.53,2.00) | 0.53(0.25,0.94) |  | 1.66(0.76,3.25) | 0.70(0.32,1.38) |  | 47.07(-22.43,208.24) | 2.09(1.46,2.72) |
| Brazil | 307.36(253.45,364.08) | 1.87(1.54,2.21) |  | 112.20(83.11,143.26) | 0.68(0.50,0.87) |  | -63.50(-73.25,-50.13) | -2.51(-2.87,-2.14) |
| Brunei Darussalam | 0.19(0.11,0.29) | 0.55(0.31,0.84) |  | 0.10(0.05,0.17) | 0.34(0.17,0.53) |  | -44.52(-70.58,9.70) | -1.23(-1.48,-0.98) |
| Bulgaria | 7.06(5.46,8.86) | 1.32(1.02,1.65) |  | 0.83(0.52,1.23) | 0.27(0.17,0.41) |  | -88.25(-92.56,-82.17) | -4.44(-4.77,-4.11) |
| Burkina Faso | 27.79(11.99,52.45) | 1.48(0.64,2.79) |  | 51.99(14.44,96.46) | 1.27(0.35,2.35) |  | 87.07(-20.15,309.33) | 0.06(-0.15,0.27) |
| Burundi | 26.09(13.99,43.67) | 2.43(1.30,4.06) |  | 22.45(5.85,51.08) | 1.04(0.27,2.36) |  | -13.95(-75.43,155.39) | -1.70(-2.19,-1.21) |
| Cabo Verde | 0.97(0.50,1.61) | 1.62(0.84,2.70) |  | 0.28(0.09,0.91) | 0.62(0.21,2.07) |  | -71.50(-91.30,-10.23) | -3.07(-3.37,-2.77) |
| Cambodia | 70.50(19.24,161.51) | 3.86(1.05,8.85) |  | 31.69(18.79,49.75) | 1.81(1.07,2.85) |  | -55.04(-80.59,93.22) | -2.76(-2.89,-2.62) |
| Cameroon | 23.85(10.96,39.95) | 1.19(0.55,1.99) |  | 45.78(14.10,79.15) | 0.94(0.29,1.63) |  | 91.96(-11.00,300.98) | -0.06(-0.32,0.20) |
| Canada | 10.28(8.80,11.82) | 0.53(0.46,0.61) |  | 2.97(2.31,3.78) | 0.16(0.12,0.20) |  | -71.10(-78.21,-61.56) | -2.83(-3.11,-2.54) |
| Central African Republic | 7.62(1.79,18.94) | 1.51(0.35,3.76) |  | 8.41(2.67,19.64) | 1.00(0.32,2.34) |  | 10.44(-32.18,150.03) | -1.03(-1.18,-0.88) |
| Chad | 15.37(6.02,30.43) | 1.24(0.48,2.45) |  | 45.37(14.79,90.07) | 1.25(0.41,2.48) |  | 195.20(52.24,571.04) | 0.49(0.34,0.63) |
| Chile | 16.02(13.71,18.69) | 1.11(0.95,1.30) |  | 3.42(2.65,4.55) | 0.31(0.24,0.42) |  | -78.62(-83.92,-70.76) | -3.28(-3.53,-3.03) |
| China | 9394.00(6094.96,13836.63) | 8.40(5.45,12.38) |  | 930.64(487.57,1433.88) | 1.20(0.63,1.85) |  | -90.09(-95.31,-82.07) | -6.14(-6.50,-5.77) |
| Colombia | 102.54(85.07,122.22) | 2.44(2.02,2.91) |  | 29.29(19.45,43.88) | 0.85(0.57,1.27) |  | -71.43(-81.81,-55.22) | -1.09(-1.87,-0.30) |
| Comoros | 1.70(0.97,2.61) | 2.06(1.18,3.17) |  | 1.10(0.42,2.26) | 1.35(0.52,2.78) |  | -35.32(-74.14,43.91) | -1.20(-1.46,-0.93) |
| Congo | 4.35(1.50,9.53) | 1.10(0.38,2.42) |  | 3.00(1.57,5.39) | 0.47(0.25,0.85) |  | -30.93(-68.40,108.54) | -2.63(-2.87,-2.39) |
| Cook Islands | 0.00(0.00,0.01) | 0.18(0.10,0.29) |  | 0.00(0.00,0.00) | 0.10(0.03,0.25) |  | -71.24(-93.86,-9.71) | -4.90(-5.97,-3.82) |
| Costa Rica | 5.95(5.03,7.00) | 1.47(1.24,1.72) |  | 2.49(1.87,3.26) | 0.81(0.61,1.06) |  | -58.21(-68.62,-42.08) | -1.49(-1.69,-1.30) |
| Croatia | 18.54(6.63,31.49) | 0.80(0.29,1.36) |  | 24.12(7.26,50.43) | 0.55(0.17,1.16) |  | -69.95(-85.44,-41.84) | -0.83(-1.15,-0.51) |
| Cuba | 1.53(0.99,2.20) | 0.51(0.33,0.73) |  | 0.46(0.25,0.78) | 0.25(0.14,0.43) |  | -79.58(-86.31,-70.87) | -2.39(-2.72,-2.05) |
| Cyprus | 10.64(8.93,12.44) | 1.19(1.00,1.39) |  | 2.17(1.51,3.01) | 0.40(0.28,0.55) |  | -64.81(-86.93,-11.47) | -2.43(-2.83,-2.02) |
| Czechia | 0.40(0.22,0.67) | 0.62(0.34,1.04) |  | 0.14(0.07,0.25) | 0.19(0.09,0.33) |  | -83.16(-90.00,-72.31) | -3.80(-3.91,-3.69) |
| C么te d'Ivoire | 6.37(4.82,8.23) | 0.98(0.74,1.27) |  | 1.07(0.68,1.67) | 0.19(0.12,0.30) |  | 30.11(-42.84,197.22) | -4.34(-4.72,-3.96) |
| Democratic People's Republic of Korea | 81.16(39.70,129.24) | 3.47(1.70,5.53) |  | 28.50(11.90,63.71) | 1.88(0.79,4.21) |  | -64.89(-82.79,-31.25) | -1.57(-1.92,-1.21) |
| Democratic Republic of the Congo | 90.62(24.24,217.03) | 1.24(0.33,2.97) |  | 72.40(31.75,145.16) | 0.53(0.23,1.07) |  | -20.11(-56.35,162.12) | -2.07(-2.36,-1.78) |
| Denmark | 1.15(0.92,1.41) | 0.40(0.32,0.49) |  | 0.55(0.38,0.75) | 0.18(0.12,0.24) |  | -52.10(-67.86,-30.76) | -2.72(-3.19,-2.25) |
| Djibouti | 1.31(0.58,2.39) | 2.03(0.90,3.70) |  | 1.57(0.52,3.75) | 1.08(0.36,2.57) |  | 19.82(-47.63,149.38) | -1.65(-2.08,-1.23) |
| Dominica | 0.13(0.09,0.18) | 1.53(1.05,2.14) |  | 0.08(0.05,0.14) | 2.40(1.46,3.90) |  | -37.16(-62.89,10.64) | 1.82(1.46,2.17) |
| Dominican Republic | 36.44(19.93,52.74) | 3.66(2.00,5.30) |  | 10.73(4.39,23.12) | 1.04(0.43,2.24) |  | -70.56(-87.98,-26.26) | -3.66(-3.94,-3.38) |
| Ecuador | 27.44(22.61,33.23) | 2.04(1.68,2.47) |  | 22.10(14.33,32.36) | 1.33(0.86,1.95) |  | -19.46(-49.49,20.46) | -1.01(-1.44,-0.57) |
| Egypt | 144.65(80.35,303.64) | 1.70(0.94,3.56) |  | 109.87(50.03,175.52) | 0.84(0.38,1.35) |  | -24.04(-77.09,62.73) | -1.12(-1.60,-0.65) |
| El Salvador | 27.34(18.96,39.30) | 3.55(2.46,5.10) |  | 5.05(2.41,8.86) | 0.84(0.40,1.47) |  | -81.53(-92.21,-59.69) | -4.41(-4.50,-4.32) |
| Equatorial Guinea | 1.01(0.25,2.47) | 1.23(0.30,3.00) |  | 0.75(0.27,1.51) | 0.40(0.14,0.81) |  | -25.91(-72.89,239.81) | -4.31(-4.63,-3.99) |
| Eritrea | 12.85(5.93,23.83) | 2.06(0.95,3.82) |  | 14.68(6.07,29.59) | 1.60(0.66,3.22) |  | 14.22(-60.61,237.26) | -0.75(-0.98,-0.53) |
| Estonia | 1.69(1.38,1.99) | 1.41(1.14,1.65) |  | 0.14(0.10,0.18) | 0.20(0.14,0.27) |  | -92.02(-94.54,-88.59) | -5.99(-6.40,-5.58) |
| Eswatini | 1.02(0.39,2.14) | 0.70(0.27,1.47) |  | 0.79(0.46,1.32) | 0.57(0.33,0.94) |  | -21.85(-59.04,104.39) | -0.28(-0.81,0.25) |
| Ethiopia | 592.30(91.02,1297.20) | 6.14(0.94,13.46) |  | 474.95(228.23,1059.09) | 2.97(1.43,6.63) |  | -19.81(-62.63,267.68) | -2.57(-2.87,-2.27) |
| Fiji | 0.65(0.18,1.30) | 0.69(0.19,1.38) |  | 0.69(0.18,1.41) | 0.76(0.20,1.55) |  | 6.88(-44.54,90.09) | 0.46(0.26,0.66) |
| Finland | 0.84(0.69,1.02) | 0.27(0.22,0.33) |  | 0.33(0.24,0.46) | 0.14(0.10,0.19) |  | -60.64(-72.52,-43.54) | -2.12(-2.45,-1.80) |
| France | 28.85(25.34,32.36) | 0.74(0.65,0.83) |  | 9.29(7.22,11.65) | 0.26(0.20,0.33) |  | -67.81(-76.13,-57.57) | -2.83(-3.29,-2.37) |
| Gabon | 1.14(0.48,2.25) | 0.73(0.31,1.44) |  | 0.95(0.42,1.68) | 0.45(0.20,0.79) |  | -16.86(-61.98,119.82) | -0.83(-1.17,-0.48) |
| Gambia | 1.40(0.55,2.35) | 0.75(0.30,1.27) |  | 1.41(0.47,3.32) | 0.39(0.13,0.93) |  | 0.46(-64.09,134.05) | -2.27(-2.59,-1.95) |
| Georgia | 9.62(7.07,12.34) | 2.05(1.51,2.63) |  | 0.81(0.55,1.13) | 0.33(0.23,0.47) |  | -91.62(-94.36,-87.44) | -5.84(-6.51,-5.17) |
| Germany | 26.75(23.05,31.03) | 0.60(0.51,0.69) |  | 9.31(7.32,12.17) | 0.23(0.18,0.30) |  | -65.20(-73.53,-53.56) | -3.28(-3.87,-2.69) |
| Ghana | 60.21(17.74,113.40) | 2.29(0.67,4.31) |  | 37.22(13.86,82.97) | 0.80(0.30,1.79) |  | -38.18(-80.25,80.43) | -3.86(-4.49,-3.24) |
| Greece | 18904.74(12759.66,28582.64) | 3.05(2.06,4.61) |  | 6294.10(3933.74,8350.67) | 0.96(0.60,1.27) |  | -66.71(-80.26,-45.45) | -3.63(-3.78,-3.48) |
| Greenland | 3.57(3.19,3.97) | 0.64(0.58,0.72) |  | 1.26(1.03,1.54) | 0.30(0.24,0.36) |  | -64.83(-71.90,-55.76) | -2.18(-2.59,-1.77) |
| Grenada | 0.03(0.02,0.05) | 0.60(0.29,0.96) |  | 0.01(0.00,0.02) | 0.21(0.11,0.37) |  | -73.71(-88.00,-45.93) | -3.11(-3.26,-2.97) |
| Guam | 0.14(0.10,0.19) | 1.21(0.87,1.60) |  | 0.03(0.02,0.05) | 0.49(0.35,0.67) |  | -76.74(-83.90,-65.86) | -1.94(-2.21,-1.68) |
| Guatemala | 0.09(0.06,0.12) | 0.54(0.40,0.74) |  | 0.07(0.04,0.11) | 0.57(0.35,0.84) |  | -16.79(-50.42,26.42) | 1.37(0.81,1.93) |
| Guinea | 43.59(36.43,52.03) | 2.84(2.38,3.39) |  | 21.86(16.06,29.06) | 1.40(1.03,1.86) |  | -49.84(-65.50,-29.06) | -1.86(-2.03,-1.69) |
| Guinea-Bissau | 10.60(3.74,18.07) | 0.91(0.32,1.56) |  | 10.10(2.20,25.84) | 0.45(0.10,1.15) |  | -4.71(-63.93,164.83) | -1.70(-1.89,-1.51) |
| Guyana | 2.96(1.15,6.11) | 1.58(0.61,3.25) |  | 2.41(0.66,4.59) | 0.72(0.20,1.38) |  | -18.63(-70.98,147.77) | -1.84(-2.32,-1.36) |
| Haiti | 1.93(1.42,2.54) | 1.71(1.26,2.25) |  | 0.90(0.60,1.32) | 1.20(0.80,1.77) |  | -53.56(-70.45,-29.19) | 0.17(-0.41,0.76) |
| Honduras | 68.10(10.62,160.78) | 6.42(1.00,15.16) |  | 60.72(15.57,139.23) | 3.87(0.99,8.87) |  | -10.83(-51.98,117.32) | -1.27(-1.48,-1.05) |
| Hungary | 26.44(17.55,38.15) | 3.21(2.13,4.64) |  | 10.75(4.15,24.09) | 0.98(0.38,2.20) |  | -59.33(-85.45,-6.75) | -3.70(-3.78,-3.61) |
| Iceland | 6.85(5.76,8.00) | 1.11(0.93,1.29) |  | 1.26(0.88,1.77) | 0.28(0.19,0.39) |  | -81.67(-87.46,-73.43) | -4.09(-4.36,-3.81) |
| India | 0.07(0.05,0.08) | 0.31(0.24,0.39) |  | 0.04(0.03,0.06) | 0.19(0.13,0.27) |  | -36.01(-57.25,-1.68) | -1.40(-1.98,-0.81) |
| Indonesia | 1447.66(720.09,2748.16) | 1.25(0.62,2.36) |  | 537.91(348.25,798.82) | 0.48(0.31,0.72) |  | -62.84(-82.16,-14.32) | -3.26(-3.38,-3.14) |
| Iran (Islamic Republic of) | 609.66(210.96,1322.30) | 2.73(0.95,5.92) |  | 322.10(190.75,494.90) | 1.47(0.87,2.26) |  | -47.17(-69.97,24.61) | -1.88(-1.97,-1.79) |
| Iraq | 305.67(181.47,494.05) | 3.48(2.07,5.63) |  | 22.01(9.77,40.04) | 0.36(0.16,0.65) |  | -92.80(-97.29,-80.86) | -5.26(-5.98,-4.55) |
| Ireland | 52.98(29.16,92.47) | 1.69(0.93,2.94) |  | 40.08(19.25,69.37) | 0.93(0.45,1.62) |  | -24.34(-67.12,94.32) | -1.79(-2.04,-1.53) |
| Israel | 1.83(1.54,2.14) | 0.63(0.53,0.74) |  | 0.39(0.30,0.54) | 0.13(0.10,0.18) |  | -78.57(-84.36,-69.48) | -4.46(-4.75,-4.16) |
| Italy | 5.16(4.24,6.22) | 1.00(0.82,1.21) |  | 1.49(1.15,1.99) | 0.16(0.12,0.22) |  | -71.05(-79.68,-59.58) | -4.85(-5.20,-4.50) |
| Jamaica | 26.69(24.65,28.61) | 0.97(0.90,1.04) |  | 6.25(4.85,7.74) | 0.29(0.22,0.36) |  | -76.58(-81.49,-70.79) | -3.82(-4.03,-3.60) |
| Japan | 5.16(4.01,6.50) | 1.85(1.44,2.33) |  | 0.99(0.67,1.44) | 0.58(0.39,0.84) |  | -80.89(-87.02,-72.06) | -3.01(-3.35,-2.67) |
| Jordan | 41.15(39.00,43.27) | 0.62(0.59,0.65) |  | 9.57(8.30,10.93) | 0.21(0.18,0.24) |  | -76.75(-79.82,-73.51) | -3.42(-3.66,-3.17) |
| Kazakhstan | 5.54(3.17,8.25) | 0.92(0.53,1.37) |  | 3.97(1.81,7.00) | 0.36(0.16,0.64) |  | -28.40(-69.59,49.22) | -3.23(-3.47,-2.99) |
| Kenya | 29.68(21.87,36.96) | 1.58(1.16,1.96) |  | 7.98(5.87,10.52) | 0.41(0.30,0.54) |  | -73.11(-81.25,-62.40) | -3.34(-3.87,-2.81) |
| Kiribati | 38.35(19.19,65.03) | 0.89(0.45,1.51) |  | 28.57(12.83,66.91) | 0.48(0.22,1.12) |  | -25.49(-66.79,70.04) | -1.04(-1.47,-0.61) |
| Kuwait | 0.13(0.07,0.22) | 1.13(0.60,1.85) |  | 0.09(0.05,0.19) | 0.66(0.33,1.33) |  | -29.17(-67.27,87.06) | -1.71(-1.97,-1.46) |
| Kyrgyzstan | 2.08(1.57,2.65) | 1.02(0.77,1.30) |  | 0.77(0.52,1.09) | 0.29(0.20,0.41) |  | -62.84(-76.30,-41.77) | -3.46(-3.89,-3.02) |
| Lao People's Democratic Republic | 12.65(9.70,16.24) | 1.97(1.51,2.53) |  | 4.36(3.03,6.15) | 0.55(0.38,0.77) |  | -65.54(-77.82,-45.53) | -4.16(-4.56,-3.75) |
| Latvia | 28.73(4.60,71.57) | 4.04(0.65,10.07) |  | 14.94(7.58,25.02) | 1.80(0.91,3.02) |  | -48.00(-75.65,207.14) | -2.61(-2.75,-2.47) |
| Lebanon | 2.78(2.21,3.38) | 1.37(1.09,1.67) |  | 0.24(0.17,0.33) | 0.25(0.18,0.36) |  | -91.42(-94.35,-86.59) | -5.12(-5.47,-4.76) |
| Lesotho | 3.45(1.76,5.93) | 0.89(0.45,1.52) |  | 1.33(0.44,2.76) | 0.33(0.11,0.68) |  | -61.62(-88.76,17.35) | -3.59(-4.01,-3.17) |
| Liberia | 1.18(0.59,2.14) | 0.48(0.24,0.87) |  | 1.04(0.54,1.80) | 0.51(0.26,0.88) |  | -12.11(-52.19,73.62) | 0.95(0.43,1.47) |
| Libya | 7.19(2.59,15.33) | 1.55(0.56,3.30) |  | 5.84(1.55,11.98) | 0.76(0.20,1.56) |  | -18.70(-75.98,256.31) | -1.99(-2.69,-1.27) |
| Lithuania | 11.62(6.06,18.51) | 1.82(0.95,2.91) |  | 6.51(2.95,11.78) | 1.54(0.70,2.78) |  | -43.97(-75.72,4.64) | -0.12(-0.31,0.07) |
| Luxembourg | 4.46(3.67,5.46) | 1.54(1.27,1.89) |  | 0.39(0.27,0.54) | 0.29(0.20,0.41) |  | -91.30(-94.32,-86.76) | -4.92(-5.49,-4.34) |
| Madagascar | 0.17(0.14,0.20) | 0.74(0.60,0.88) |  | 0.06(0.04,0.08) | 0.17(0.12,0.23) |  | -66.68(-76.50,-53.41) | -4.91(-5.38,-4.44) |
| Malawi | 45.29(26.03,71.68) | 2.10(1.21,3.33) |  | 44.82(18.54,86.85) | 1.10(0.45,2.12) |  | -1.03(-61.82,125.75) | -1.64(-1.86,-1.41) |
| Malaysia | 26.94(14.20,40.68) | 1.42(0.75,2.14) |  | 14.14(3.84,35.14) | 0.52(0.14,1.29) |  | -47.52(-84.29,36.68) | -3.17(-3.42,-2.92) |
| Maldives | 27.03(12.78,46.94) | 1.13(0.54,1.97) |  | 11.26(6.30,17.46) | 0.46(0.26,0.71) |  | -58.35(-81.34,9.62) | -2.16(-2.78,-1.54) |
| Mali | 0.99(0.25,2.25) | 2.38(0.60,5.37) |  | 0.26(0.14,0.48) | 0.81(0.43,1.52) |  | -74.02(-91.74,39.37) | -3.32(-3.47,-3.17) |
| Malta | 27.15(9.77,45.77) | 1.57(0.57,2.65) |  | 30.81(7.76,74.93) | 0.67(0.17,1.64) |  | 13.47(-52.38,169.36) | -2.33(-2.51,-2.16) |
| Marshall Islands | 0.16(0.12,0.21) | 0.56(0.41,0.73) |  | 0.05(0.03,0.06) | 0.21(0.14,0.29) |  | -70.97(-80.34,-57.00) | -2.58(-2.86,-2.30) |
| Mauritania | 0.03(0.02,0.05) | 0.40(0.24,0.65) |  | 0.02(0.01,0.04) | 0.38(0.19,0.64) |  | -28.55(-62.21,33.05) | -0.38(-0.97,0.20) |
| Mauritius | 3.37(1.48,5.63) | 0.91(0.40,1.52) |  | 4.04(1.16,9.09) | 0.62(0.18,1.38) |  | 20.08(-58.15,155.50) | -1.21(-1.58,-0.84) |
| Mexico | 1.20(1.04,1.41) | 1.14(0.99,1.33) |  | 0.32(0.25,0.38) | 0.49(0.39,0.60) |  | -73.72(-80.37,-66.37) | -1.26(-3.23,0.74) |
| Micronesia (Federated States of) | 439.13(385.08,506.26) | 3.72(3.26,4.29) |  | 138.12(97.05,194.01) | 1.40(0.98,1.96) |  | -68.55(-77.85,-54.39) | -2.85(-3.29,-2.40) |
| Monaco | 0.11(0.06,0.18) | 0.70(0.37,1.14) |  | 0.03(0.01,0.05) | 0.30(0.15,0.55) |  | -74.02(-88.21,-34.87) | -2.62(-2.82,-2.42) |
| Mongolia | 0.02(0.01,0.04) | 2.09(0.97,3.72) |  | 0.02(0.01,0.03) | 1.20(0.55,2.09) |  | -20.77(-62.34,71.15) | -3.61(-4.45,-2.76) |
| Montenegro | 9.09(4.84,16.53) | 2.68(1.43,4.87) |  | 2.26(1.13,3.78) | 0.58(0.29,0.97) |  | -75.16(-90.46,-40.57) | -4.89(-5.25,-4.53) |
| Morocco | 0.56(0.33,0.88) | 1.07(0.63,1.67) |  | 0.07(0.03,0.12) | 0.19(0.09,0.33) |  | -87.39(-94.54,-73.78) | -4.07(-4.55,-3.59) |
| Mozambique | 19.63(9.02,39.16) | 0.55(0.25,1.10) |  | 6.23(2.28,13.12) | 0.19(0.07,0.40) |  | -68.27(-88.86,4.22) | -2.94(-3.17,-2.70) |
| Myanmar | 171.27(87.81,284.55) | 7.11(3.64,11.81) |  | 155.22(42.80,381.75) | 3.00(0.83,7.37) |  | -9.37(-74.64,165.63) | -2.46(-2.65,-2.28) |
| Namibia | 269.57(45.46,650.19) | 5.35(0.90,12.89) |  | 129.71(64.15,228.79) | 2.48(1.23,4.38) |  | -51.88(-77.66,150.02) | -2.63(-2.78,-2.47) |
| Nauru | 1.44(0.65,2.75) | 0.64(0.29,1.22) |  | 1.67(0.82,2.90) | 0.60(0.29,1.04) |  | 15.92(-48.13,198.39) | 0.79(0.08,1.50) |
| Nepal | 0.01(0.01,0.02) | 0.83(0.48,1.35) |  | 0.01(0.01,0.02) | 0.68(0.39,1.08) |  | -30.02(-60.55,19.68) | -0.75(-1.38,-0.11) |
| Netherlands | 59.52(16.77,139.13) | 1.81(0.51,4.22) |  | 15.79(8.25,25.27) | 0.51(0.27,0.81) |  | -73.48(-90.93,7.62) | -3.84(-4.01,-3.67) |
| New Zealand | 4.67(3.88,5.50) | 0.50(0.41,0.59) |  | 1.02(0.75,1.37) | 0.12(0.09,0.16) |  | -78.10(-84.38,-69.04) | -4.42(-4.68,-4.16) |
| Nicaragua | 2.02(1.78,2.31) | 0.72(0.64,0.82) |  | 0.75(0.62,0.91) | 0.24(0.20,0.29) |  | -62.74(-70.13,-52.92) | -2.90(-3.35,-2.45) |
| Niger | 31.89(20.93,45.72) | 4.81(3.15,6.89) |  | 6.44(3.32,11.41) | 0.99(0.51,1.75) |  | -79.80(-90.95,-56.32) | -4.69(-4.89,-4.49) |
| Nigeria | 32.64(13.09,68.91) | 1.93(0.78,4.08) |  | 51.48(13.47,98.83) | 1.01(0.26,1.94) |  | 57.75(-44.65,405.31) | -1.85(-2.06,-1.64) |
| Niue | 284.13(98.16,566.14) | 1.79(0.62,3.56) |  | 530.53(126.29,837.87) | 1.43(0.34,2.26) |  | 86.72(-5.63,347.80) | -0.46(-0.57,-0.35) |
| North Macedonia | 0.00(0.00,0.00) | 0.53(0.30,0.87) |  | 0.00(0.00,0.00) | 1.92(1.14,3.41) |  | 67.96(-0.01,196.55) | 0.86(-0.30,2.03) |
| Northern Mariana Islands | 3.18(1.98,4.73) | 1.86(1.16,2.78) |  | 0.22(0.09,0.41) | 0.22(0.09,0.41) |  | -93.17(-97.62,-83.75) | -4.79(-5.38,-4.19) |
| Norway | 0.01(0.01,0.02) | 0.25(0.14,0.40) |  | 0.00(0.00,0.01) | 0.13(0.07,0.20) |  | -64.40(-78.22,-42.77) | -1.65(-1.96,-1.34) |
| Oman | 1.10(0.99,1.21) | 0.40(0.36,0.44) |  | 0.39(0.32,0.48) | 0.14(0.11,0.17) |  | -64.66(-71.58,-55.33) | -2.92(-3.21,-2.62) |
| Pakistan | 1.00(0.39,1.96) | 0.31(0.12,0.60) |  | 0.34(0.14,0.60) | 0.08(0.03,0.14) |  | -65.66(-87.42,3.00) | -3.12(-3.96,-2.27) |
| Palau | 269.66(112.14,534.59) | 1.46(0.61,2.90) |  | 324.29(169.96,530.64) | 1.09(0.57,1.79) |  | 20.26(-28.89,138.77) | -0.20(-0.54,0.15) |
| Palestine | 0.00(0.00,0.01) | 0.18(0.01,0.90) |  | 0.00(0.00,0.00) | 0.10(0.01,0.47) |  | -65.44(-88.56,8.25) | -1.75(-2.00,-1.49) |
| Panama | 8.68(3.94,15.35) | 2.23(1.01,3.93) |  | 5.31(3.22,8.26) | 0.87(0.52,1.35) |  | -38.83(-71.65,47.25) | -2.54(-2.85,-2.23) |
| Papua New Guinea | 6.03(4.81,7.45) | 2.11(1.69,2.61) |  | 4.75(3.59,6.26) | 1.28(0.97,1.69) |  | -21.24(-44.42,11.87) | -1.45(-1.61,-1.29) |
| Paraguay | 7.14(2.45,14.77) | 1.10(0.38,2.28) |  | 14.02(5.77,27.43) | 0.92(0.38,1.80) |  | 96.29(24.20,264.34) | -0.50(-0.87,-0.14) |
| Peru | 14.74(10.07,20.97) | 2.39(1.63,3.40) |  | 9.10(5.17,16.03) | 1.40(0.80,2.47) |  | -38.24(-67.58,16.29) | -0.93(-1.32,-0.54) |
| Philippines | 106.23(67.50,178.91) | 3.63(2.31,6.11) |  | 37.39(15.17,66.14) | 1.13(0.46,2.00) |  | -64.81(-89.67,-20.55) | -3.06(-3.43,-2.69) |
| Poland | 256.54(131.65,454.73) | 2.77(1.42,4.92) |  | 173.67(109.96,239.35) | 1.55(0.98,2.13) |  | -32.30(-66.07,56.64) | -1.02(-1.32,-0.72) |
| Portugal | 34.16(24.36,43.08) | 1.17(0.83,1.48) |  | 5.74(4.48,7.16) | 0.30(0.24,0.38) |  | -83.21(-88.56,-73.55) | -4.82(-5.20,-4.44) |
| Puerto Rico | 7.32(6.16,8.66) | 1.26(1.07,1.50) |  | 1.20(0.90,1.62) | 0.28(0.21,0.38) |  | -83.54(-88.33,-77.17) | -4.74(-5.48,-4.00) |
| Qatar | 2.91(2.34,3.53) | 0.91(0.73,1.11) |  | 0.30(0.22,0.40) | 0.29(0.21,0.38) |  | -89.65(-92.87,-84.46) | -2.77(-3.11,-2.43) |
| Republic of Korea | 0.20(0.11,0.34) | 0.40(0.22,0.68) |  | 0.20(0.09,0.42) | 0.11(0.05,0.23) |  | -0.85(-60.25,116.02) | -3.93(-4.13,-3.74) |
| Republic of Moldova | 26.89(15.92,38.87) | 0.81(0.48,1.17) |  | 2.59(0.90,4.70) | 0.17(0.06,0.30) |  | -90.38(-96.42,-78.69) | -4.45(-4.68,-4.22) |
| Romania | 14.69(11.96,17.75) | 3.41(2.78,4.12) |  | 1.02(0.73,1.40) | 0.66(0.48,0.91) |  | -93.05(-95.25,-89.76) | -4.75(-5.23,-4.28) |
| Russian Federation | 36.21(30.24,43.52) | 2.05(1.71,2.46) |  | 3.54(2.74,4.61) | 0.38(0.29,0.49) |  | -90.23(-92.78,-86.44) | -5.12(-5.44,-4.80) |
| Rwanda | 200.50(186.13,213.59) | 1.72(1.60,1.84) |  | 33.28(28.45,38.53) | 0.44(0.37,0.51) |  | -83.40(-85.68,-81.06) | -5.29(-6.03,-4.54) |
| Saint Kitts and Nevis | 34.07(18.97,57.06) | 2.53(1.41,4.23) |  | 22.35(8.01,45.75) | 1.28(0.46,2.62) |  | -34.39(-79.69,88.52) | -2.39(-2.78,-1.99) |
| Saint Lucia | 0.09(0.07,0.10) | 1.82(1.47,2.23) |  | 0.02(0.02,0.03) | 0.79(0.54,1.13) |  | -71.64(-81.07,-57.69) | -1.79(-2.07,-1.51) |
| Saint Vincent and the Grenadines | 0.23(0.18,0.30) | 1.32(1.00,1.69) |  | 0.04(0.02,0.06) | 0.43(0.28,0.65) |  | -83.70(-90.16,-73.17) | -2.74(-2.98,-2.51) |
| Samoa | 0.16(0.12,0.21) | 1.24(0.95,1.66) |  | 0.03(0.02,0.05) | 0.44(0.31,0.63) |  | -79.84(-86.93,-70.02) | -2.96(-3.30,-2.63) |
| San Marino | 0.20(0.11,0.33) | 0.77(0.45,1.30) |  | 0.12(0.06,0.26) | 0.42(0.19,0.88) |  | -37.59(-70.13,46.18) | -1.86(-1.98,-1.73) |
| Sao Tome and Principe | 0.03(0.02,0.05) | 2.36(1.33,3.80) |  | 0.01(0.00,0.01) | 0.67(0.35,1.12) |  | -71.11(-86.45,-33.14) | -3.27(-3.48,-3.06) |
| Saudi Arabia | 0.19(0.09,0.32) | 0.91(0.46,1.56) |  | 0.05(0.01,0.14) | 0.20(0.04,0.56) |  | -73.65(-95.00,-18.40) | -4.71(-5.39,-4.02) |
| Senegal | 30.83(17.62,50.71) | 1.28(0.73,2.10) |  | 5.03(2.26,10.23) | 0.21(0.09,0.42) |  | -83.68(-93.61,-58.68) | -5.82(-5.94,-5.70) |
| Serbia | 22.25(9.62,38.83) | 1.52(0.66,2.65) |  | 16.24(4.28,39.13) | 0.71(0.19,1.72) |  | -27.00(-78.16,125.23) | -1.91(-2.27,-1.55) |
| Seychelles | 8.29(4.33,13.72) | 1.22(0.64,2.02) |  | 0.60(0.28,1.01) | 0.16(0.08,0.27) |  | -92.78(-97.13,-82.32) | -6.94(-7.63,-6.24) |
| Sierra Leone | 0.05(0.03,0.08) | 0.60(0.34,1.01) |  | 0.03(0.02,0.05) | 0.41(0.22,0.67) |  | -33.31(-66.22,24.40) | -0.69(-0.87,-0.50) |
| Singapore | 13.61(5.23,27.64) | 1.76(0.68,3.57) |  | 15.36(4.07,29.27) | 1.14(0.30,2.18) |  | 12.85(-56.43,251.31) | -1.12(-1.31,-0.92) |
| Slovakia | 1.94(1.55,2.38) | 0.95(0.76,1.17) |  | 0.57(0.44,0.71) | 0.20(0.15,0.25) |  | -70.79(-79.19,-59.77) | -4.51(-5.05,-3.96) |
| Slovenia | 2.96(1.98,4.12) | 0.72(0.48,1.01) |  | 0.84(0.45,1.42) | 0.29(0.16,0.49) |  | -71.68(-86.42,-46.45) | -2.64(-2.78,-2.50) |
| Solomon Islands | 0.83(0.67,1.00) | 0.68(0.55,0.81) |  | 0.17(0.12,0.24) | 0.17(0.12,0.24) |  | -80.08(-86.57,-70.85) | -4.81(-5.19,-4.43) |
| Somalia | 0.40(0.21,0.67) | 0.67(0.36,1.12) |  | 0.40(0.21,0.76) | 0.42(0.22,0.79) |  | 1.10(-47.14,138.09) | -1.43(-1.75,-1.09) |
| South Africa | 33.59(13.84,68.27) | 2.17(0.90,4.42) |  | 46.28(21.46,84.63) | 1.12(0.52,2.05) |  | 37.77(-34.43,215.88) | -1.40(-1.89,-0.92) |
| South Sudan | 35.45(15.87,68.70) | 0.73(0.33,1.41) |  | 22.05(14.56,31.00) | 0.44(0.29,0.62) |  | -37.80(-64.71,43.79) | -0.56(-1.45,0.33) |
| Spain | 28.53(13.42,54.92) | 2.80(1.32,5.40) |  | 35.99(17.77,67.38) | 2.30(1.14,4.31) |  | 26.13(-23.76,125.11) | 0.07(-0.55,0.69) |
| Sri Lanka | 18.33(15.83,21.30) | 0.88(0.76,1.02) |  | 4.64(3.57,6.01) | 0.25(0.19,0.33) |  | -74.68(-81.38,-65.91) | -4.10(-4.32,-3.87) |
| Sudan | 26.30(17.93,38.25) | 1.49(1.01,2.16) |  | 5.27(2.60,9.70) | 0.34(0.17,0.62) |  | -79.96(-90.56,-59.14) | -4.48(-5.02,-3.93) |
| Suriname | 156.42(33.13,382.04) | 4.49(0.95,10.98) |  | 103.43(42.92,198.15) | 1.83(0.76,3.51) |  | -33.88(-69.37,186.37) | -2.52(-2.77,-2.27) |
| Sweden | 0.61(0.30,0.86) | 1.38(0.69,1.96) |  | 0.38(0.22,0.59) | 0.85(0.49,1.32) |  | -37.28(-63.80,21.23) | -1.58(-1.74,-1.43) |
| Switzerland | 4.33(3.75,5.04) | 0.77(0.67,0.89) |  | 1.03(0.82,1.31) | 0.18(0.14,0.23) |  | -76.12(-82.02,-68.95) | -4.66(-5.46,-3.86) |
| Syrian Arab Republic | 2.21(1.82,2.65) | 0.55(0.46,0.67) |  | 0.87(0.62,1.21) | 0.20(0.14,0.27) |  | -60.57(-73.49,-41.64) | -3.71(-4.22,-3.19) |
| Taiwan (Province of China) | 46.56(21.89,83.24) | 2.16(1.02,3.86) |  | 6.57(3.23,11.69) | 0.65(0.32,1.16) |  | -85.89(-91.95,-74.66) | -3.35(-3.96,-2.74) |
| Tajikistan | 5.36(3.51,7.60) | 0.33(0.22,0.47) |  | 2.06(1.53,2.72) | 0.23(0.17,0.30) |  | -61.57(-76.90,-34.78) | -0.41(-0.91,0.09) |
| Thailand | 31.42(15.74,46.72) | 3.32(1.66,4.94) |  | 18.96(9.29,37.93) | 1.42(0.69,2.83) |  | -39.63(-71.57,22.73) | -2.73(-3.00,-2.45) |
| Timor-Leste | 64.81(37.02,106.76) | 1.25(0.71,2.05) |  | 17.45(9.95,26.23) | 0.62(0.35,0.93) |  | -73.08(-86.53,-49.55) | -2.05(-2.25,-1.86) |
| Togo | 5.96(1.04,14.10) | 4.26(0.75,10.07) |  | 3.80(1.86,6.78) | 2.05(1.01,3.67) |  | -36.34(-69.22,196.57) | -2.68(-3.01,-2.35) |
| Tokelau | 7.84(3.26,13.52) | 1.14(0.47,1.97) |  | 9.16(2.21,19.16) | 0.78(0.19,1.63) |  | 16.87(-56.46,190.55) | -0.94(-1.09,-0.79) |
| Tonga | 0.00(0.00,0.00) | 0.66(0.33,1.14) |  | 0.00(0.00,0.01) | 2.71(0.82,6.80) |  | 107.39(-39.31,466.01) | -0.35(-2.28,1.61) |
| Trinidad and Tobago | 0.05(0.03,0.09) | 0.33(0.19,0.58) |  | 0.04(0.02,0.06) | 0.25(0.14,0.45) |  | -30.22(-68.85,53.77) | -1.06(-1.40,-0.72) |
| Tunisia | 1.53(1.26,1.86) | 1.15(0.95,1.39) |  | 0.45(0.30,0.64) | 0.56(0.37,0.79) |  | -70.76(-81.29,-55.55) | -2.48(-2.96,-2.00) |
| Turkey | 16.20(8.35,26.94) | 1.52(0.78,2.52) |  | 3.15(1.15,6.15) | 0.35(0.13,0.69) |  | -80.57(-93.03,-49.73) | -4.39(-4.48,-4.29) |
| Turkmenistan | 245.46(122.60,426.10) | 3.53(1.76,6.13) |  | 38.41(22.46,59.29) | 0.69(0.40,1.07) |  | -84.35(-93.41,-61.89) | -5.16(-5.58,-4.75) |
| Tuvalu | 10.49(7.07,15.53) | 1.79(1.21,2.65) |  | 3.43(2.46,4.96) | 0.64(0.46,0.92) |  | -67.29(-78.24,-46.99) | -3.11(-3.43,-2.80) |
| Uganda | 0.01(0.00,0.03) | 0.93(0.28,1.92) |  | 0.00(0.00,0.01) | 0.33(0.19,0.50) |  | -70.28(-86.89,14.70) | -3.14(-3.28,-3.01) |
| Ukraine | 59.82(31.02,95.84) | 1.67(0.86,2.67) |  | 78.06(25.16,166.32) | 1.07(0.34,2.27) |  | 30.49(-49.46,189.18) | -1.02(-1.28,-0.75) |
| United Arab Emirates | 101.60(76.61,128.32) | 2.70(2.03,3.40) |  | 12.18(8.76,17.66) | 0.77(0.55,1.11) |  | -88.01(-91.98,-81.81) | -3.72(-4.11,-3.33) |
| United Kingdom | 1.79(0.96,3.26) | 0.79(0.42,1.43) |  | 0.90(0.37,1.65) | 0.21(0.09,0.38) |  | -49.58(-81.90,32.31) | -2.83(-3.33,-2.34) |
| United Republic of Tanzania | 24.58(22.80,26.70) | 0.64(0.59,0.70) |  | 6.80(5.97,7.71) | 0.19(0.16,0.21) |  | -72.32(-76.16,-68.19) | -3.30(-3.68,-2.93) |
| United States of America | 129.56(77.62,195.61) | 2.69(1.61,4.07) |  | 147.80(60.30,279.18) | 1.67(0.68,3.16) |  | 14.07(-55.94,163.00) | -0.84(-1.11,-0.56) |
| United States Virgin Islands | 122.22(118.01,126.34) | 0.62(0.60,0.64) |  | 45.78(40.37,51.88) | 0.25(0.22,0.28) |  | -62.55(-67.05,-57.77) | -2.37(-2.56,-2.19) |
| Uruguay | 0.08(0.04,0.12) | 0.69(0.38,1.06) |  | 0.02(0.01,0.03) | 0.39(0.14,0.80) |  | -79.85(-92.14,-53.30) | -1.38(-1.52,-1.23) |
| Uzbekistan | 2.60(2.17,3.09) | 0.95(0.79,1.13) |  | 0.64(0.46,0.90) | 0.33(0.23,0.46) |  | -75.49(-82.99,-64.31) | -3.31(-3.56,-3.05) |
| Vanuatu | 67.38(44.42,99.69) | 2.00(1.32,2.96) |  | 46.40(30.49,69.74) | 1.21(0.80,1.82) |  | -31.14(-60.36,15.58) | -1.42(-1.76,-1.09) |
| Venezuela (Bolivarian Republic of) | 0.12(0.07,0.19) | 0.44(0.24,0.71) |  | 0.13(0.07,0.21) | 0.31(0.17,0.50) |  | 7.59(-37.87,105.30) | -1.03(-1.62,-0.44) |
| Viet Nam | 47.46(41.33,53.69) | 1.88(1.63,2.12) |  | 28.58(15.97,43.39) | 1.31(0.73,1.99) |  | -39.77(-66.66,-7.83) | -0.76(-1.17,-0.35) |
| Yemen | 152.40(79.82,273.23) | 1.62(0.85,2.90) |  | 58.39(31.70,94.90) | 0.72(0.39,1.17) |  | -61.68(-81.79,-17.28) | -2.52(-2.68,-2.36) |
| Zambia | 82.98(22.88,202.87) | 2.99(0.82,7.31) |  | 66.76(28.10,119.80) | 1.42(0.60,2.55) |  | -19.56(-60.28,203.96) | -2.22(-2.38,-2.06) |
| Zimbabwe | 44.87(24.97,75.91) | 2.97(1.65,5.03) |  | 38.96(15.78,79.44) | 1.33(0.54,2.72) |  | -13.18(-69.90,140.99) | -2.18(-2.37,-1.99) |
